# Supplementary material for: Determinants of vaccine hesitancy in Switzerland: study protocol of a mixed-methods national research programme
Source: BMJ Open. 2019 Nov 2;9(11):e032218. doi: 10.1136/bmjopen-2019-032218 (PMC6830664; doi:10.1136/bmjopen-2019-032218)
Supplement: Supplementary data [file bmjopen-2019-032218supp001.pdf]

Appendix 1

This appendix presents the questions we developed based on preliminary findings from the qualitative phase of research by theme.

For both the childhood and the HPV vaccination questionnaires, we developed questions addressing four themes.

Theme 1: Communication about vaccination with medical providers

Q1

Have you ever discussed childhood vaccination with your child`s doctor?	1. yes<br>2. no<br>3. doesn't want to disclose<br>4. doesn't know<br>5. missing	Avez-vous déjà discuté de la vaccination pour enfants avec le médecin de votre enfant ?	1. oui<br>2. non<br>3. ne veut pas divulguer<br>4. ne sait pas<br>5. missing
Haben Sie je eine Impfung für Kinder mit dem Arzt/ der Ärztin Ihres Kindes besprochen?	1. ja<br>2. nein<br>3. möchte die Frage nicht beantworten<br>4. weiss nicht<br>5. keine Antwort	Le è già capitato di discutere della vaccinazione infantile con il medico suo/a figlio/a?	1. si<br>2. no<br>3. non vuole rivelare<br>4. non sa<br>5. mancante
Asked in:	✓ Childhood vaccinations		

Have you ever discussed HPV vaccination with your teen`s doctor?	1. yes<br>2. no<br>3. doesn't want to disclose<br>4. doesn't know<br>5. missing	Avez-vous déjà discuté de la vaccination HPV avec le médecin de votre enfant?	1. oui<br>2. non<br>3. ne veut pas divulguer<br>4. ne sait pas<br>5. missing
Haben Sie je eine HPV Impfung mit dem Arzt/ der Ärztin Ihres Kindes besprochen?	1. ja<br>2. nein<br>3. möchte die Frage nicht beantworten<br>4. weiss nicht<br>5. keine Antwort	Le è già capitato di discutere della vaccinazione contro l`HPV con il medico di suo/a figlio/a?	1. si<br>2. no<br>3. non vuole rivelare<br>4. non sa<br>5. mancante
Asked in:	✓ HPV parent		

If yes of Q1, ask Q2-5

Q2

How strongly does your child`s doctor recommend vaccinating your child with all the recommended vaccines?<br><br>READ OUT ANSWER OPTIONS	1. Supports all recommended vaccines<br>2. Supports most recommended vaccines<br>3. Supports some recommended vaccines<br>4. Doesn't support any recommended vaccines<br>5. doesn't want to disclose<br>6. doesn't know<br>7. missing	Comment est-ce que vous caractériseriez le niveau de soutien au médecin de votre enfant votre enfant pour les vaccins? Est-il/elle…<br><br>LIRE TOUTES LES REPONSES POSSIBLES	1. Soutient toutes les vaccins recommandés<br>2. Soutient la plupart des vaccins recommandés<br>3. Soutient quelques-uns des vaccins recommandés<br>4. Ne soutient aucun vaccin recommandé<br>5. ne veut pas divulguer<br>6. ne sait pas<br>7. missing
Wie würden Sie das Niveau der Unterstützung/ die Unterstützung für Impfungen vom Arzt/ von der Ärztin Ihres Kindes einschätzen? Ist er/sie...<br><br>ANTWORTMÖGLICHKEITEN VORLESEN	1. Unterstützt alle empfohlenen Impfungen<br>2. Unterstützt die meisten empfohlenen Impfungen<br>3. Unterstützt ein paar der empfohlenen Impfungen<br>4. Unterstützt gar keine Impfungen<br>5. möchte die Frage nicht beantworten<br>6. weiss nicht<br>7. keine Antwort	Come qualificherebbe il livello di sostegno del medico di suo/a figlio/a alla vaccinazione?<br><br>LEGGERE LE RISPOSTE POSSIBILI	1. Sostiene tutti i vaccini raccomandati<br>2. Sostiene la maggior parte dei vaccini raccomandati<br>3. Sostiene alcuni dei vaccini raccomandati<br>4. Non sostiene alcun vaccino raccomandato<br>5. non vuole rivelare<br>6. non sa<br>7. mancante

Asked in:	✓ Childhood vaccinations

How strongly does your teen`s doctor recommend vaccinating your	1. Supports all recommended vaccines	Comment est-ce que vous caractériseriez le niveau de soutien	1. Soutient toutes les vaccins recommandés

Deml MJ, *et al. BMJ Open* 2019; 9:e032218. doi: 10.1136/bmjopen-2019-032218

teenager with all the recommended vaccines?<br><br>READ OUT ANSWER OPTIONS	2. Supports most recommended vaccines<br>3. Supports some recommended vaccines<br>4. Doesn't support any recommended vaccines<br>5. doesn't want to disclose<br>6. doesn't know<br>7. missing	au médecin de votre enfant votre enfant pour les vaccins? Est-il/elle…<br><br>LIRE TOUTES LES REPONSES POSSIBLES	2. Soutient la plupart des vaccins recommandés<br>3. Soutient quelques-uns des vaccins recommandés<br>4. Ne soutient aucun vaccin recommandé<br>5. ne veut pas divulguer<br>6. ne sait pas<br>7. missing
Wie würden Sie das Niveau der Unterstützung/ die Unterstützung für Impfungen vom Arzt/ von der Ärztin Ihres Kindes einschätzen? Ist er/sie...<br><br>ANTWORTMÖGLICHKEITEN VORLESEN	1. Unterstützt alle empfohlenen Impfungen<br>2. Unterstützt die meisten empfohlenen Impfungen<br>3. Unterstützt ein paar der empfohlenen Impfungen<br>4. Unterstützt gar keine Impfungen<br>5. möchte die Frage nicht beantworten<br>6. weiss nicht<br>7. keine Antwort	Come qualificherebbe il livello di sostegno del medico di suo/a figlio/a alla vaccinazione?<br><br>LEGGERE LE RISPOSTE POSSIBILI	1. Sostiene tutti i vaccini raccomandati<br>2. Sostiene la maggior parte dei vaccini raccomandati<br>3. Sostiene alcuni dei vaccini raccomandati<br>4. Non sostiene alcun vaccino raccomandato<br>5. non vuole rivelare<br>6. non sa<br>7. mancante

Asked in:	✓ HPV parent

Q3

How important is following the recommended vaccination	1. Very important<br>2. Somewhat important<br>3. Not very important	Quelle importance a-t-il de suivre exactement le plan de	1. Très important<br>2. Relativement important<br>3. Peu important

Deml MJ, *et al. BMJ Open* 2019; 9:e032218. doi: 10.1136/bmjopen-2019-032218

schedule for your child`s doctor?<br><br>READ OUT ANSWER OPTIONS	4. Not important at all<br>5. doesn't want to disclose<br>6. doesn't know<br>7. missing	vaccination pour le médecin de votre enfant?<br><br>LIRE TOUTES LES REPONSES POSSIBLES	4. Pas du tout important<br>5. ne veut pas divulguer<br>6. ne sait pas<br>7. missing
Wie wichtig ist es für den Arzt/ die Ärztin Ihres Kindes, den Impfplan genau einzuhalten?<br><br>ANTWORTMÖGLICHKEITEN VORLESEN	1. Sehr wichtig<br>2. Eher wichtig<br>3. Eher unwichtig<br>4. Gar nicht wichtig<br>5. möchte die Frage nicht beantworten<br>6. weiss nicht<br>7. keine Antwort	Quanto ritiene sia importante per il medico di suo/a figlio/a di seguire esattamente il programma di vaccinazione?<br><br>LEGGERE LE RISPOSTE POSSIBILI	1. Molto importante<br>2. Abbastanza importante<br>3. Non molto importante<br>4. Per niente importante<br>5. non vuole rivelare<br>6. non sa<br>7. mancante

Asked in:	✓ Childhood vaccinations

How important is following the recommended vaccination schedule for your child`s doctor?<br><br>READ OUT ANSWER OPTIONS	1. Very important<br>2. Somewhat important<br>3. Not very important<br>4. Not important at all<br>5. doesn't want to disclose<br>6. doesn't know<br>7. missing	Quelle importance a-t-il de suivre exactement le plan de vaccination pour le médecin de votre enfant?<br><br>LIRE TOUTES LES REPONSES POSSIBLES	1. Très important<br>2. Relativement important<br>3. Peu important<br>4. Pas du tout important<br>5. ne veut pas divulguer<br>6. ne sait pas<br>7. missing
Wie wichtig ist es für den Arzt/ die Ärztin Ihres Kindes, den Impfplan genau einzuhalten?	1. Sehr wichtig<br>2. Eher wichtig<br>3. Eher unwichtig<br>4. Gar nicht wichtig<br>5. möchte die Frage nicht beantworten<br>6. weiss nicht<br>7. keine Antwort	Quanto ritiene sia importante per il medico di suo/a figlio/a di seguire esattamente il programma di vaccinazione?	1. Molto importante<br>2. Abbastanza importante<br>3. Non molto importante<br>4. Per niente importante

Deml MJ, *et al. BMJ Open* 2019; 9:e032218. doi: 10.1136/bmjopen-2019-032218

ANTWORTMÖGLICHKEITEN VORLESEN		LEGGERE LE RISPOSTE POSSIBILI	5. non vuole rivelare<br>6. non sa<br>7. mancante

Asked in:	✓ HPV parent

Q4

How much do you trust the information your child`s doctor gave you?<br><br>READ OUT ANSWER OPTIONS	1. Completely trust<br>2. Somewhat trust<br>3. Neither trust nor distrust<br>4. Somewhat distrust<br>5. Don't trust at all<br>6. doesn't want to disclose<br>7. doesn't know<br>8. missing	A quel point faites-vous confiance aux informations que au médecin de votre enfant vous a données?<br><br>LIRE TOUTES LES REPONSES POSSIBLES	1. Fait entièrement confiance<br>2. Fait un peu confiance<br>3. Ne fait ni confiance, ni ne fait pas confiance<br>4. Ne fait pas vraiment confiance<br>5. Ne fait pas du tout confiance<br>6. ne veut pas divulguer<br>7. ne sait pas<br>8. missing
Wie sehr vertrauen Sie der Information die der Arzt/ die Ärztin Ihres KindesIhnen gegeben hat?<br><br>ANTWORTMÖGLICHKEITEN VORLESEN	1. Ich habe sehr grosses Vertrauen<br>2. Ich habe einiges Vertrauen<br>3. Weder Vertrauen noch Misstrauen<br>4. Ich habe etwas Misstrauen<br>5. Ich habe gar kein Vertrauen<br>6. möchte die Frage nicht beantworten<br>7. weiss nicht<br>8. keine Antwort	Quanto si fida delle informazioni che il medico di suo/a figlio/a le ha dato?<br><br>LEGGERE LE RISPOSTE POSSIBILI	1. Si fida completamente<br>2. Si fida in parte<br>3. Né si fida né diffida<br>4. Diffida in parte<br>5. Diffida completamente<br>6. non vuole rivelare<br>7. non sa<br>8. mancante

Asked in:	✓ Childhood vaccinations

Deml MJ, *et al. BMJ Open* 2019; 9:e032218. doi: 10.1136/bmjopen-2019-032218

How much do you trust the information your teen`s doctor gave you?<br><br>READ OUT ANSWER OPTIONS	1. Completely trust<br>2. Somewhat trust<br>3. Neither trust nor distrust<br>4. Somewhat distrust<br>5. Don't trust at all<br>6. doesn't want to disclose<br>7. doesn't know<br>8. missing	A quel point faites-vous confiance aux informations que le médecin de votre enfant vous a données?<br><br>LIRE TOUTES LES REPONSES POSSIBLES	1. Fait entièrement confiance<br>2. Fait un peu confiance<br>3. Ne fait ni confiance, ni ne fait pas confiance<br>4. Ne fait pas vraiment confiance<br>5. Ne fait pas du tout confiance<br>6. ne veut pas divulguer<br>7. ne sait pas<br>8. missing
Wie sehr vertrauen Sie der Information die der Arzt/ die Ärztin Ihres Kindes Ihnen gegeben hat?<br><br>ANTWORTMÖGLICHKEITEN VORLESEN	1. Ich habe sehr grosses Vertrauen<br>2. Ich habe einiges Vertrauen<br>3. Weder Vertrauen noch Misstrauen<br>4. Ich habe etwas Misstrauen<br>5. Ich habe gar kein Vertrauen<br>6. möchte die Frage nicht beantworten<br>7. weiss nicht<br>8. keine Antwort	Quanto si fida delle informazioni che il medico di suo figlio/a le ha dato?<br><br>LEGGERE LE RISPOSTE POSSIBILI	1. Si fida completamente<br>2. Si fida in parte<br>3. Né si fida né diffida<br>4. Diffida in parte<br>5. Diffida completamente<br>6. non vuole rivelare<br>7. non sa<br>8. mancante

Asked in:	✓ HPV parent

Q5

How satisfied were you with your discussions about vaccines with your child`s doctor?	1. Not at all satisfied<br>2. Somewhat satisfied<br>3. Neither satisfied nor unsatisfied<br>4. Satisfied<br>5. Very satisfied<br>6. doesn't want to disclose<br>7. doesn't know	A quel point avez-vous été satisfait.e des discussions sur les vaccins avec le médecin de votre enfant?	1. Pas du tout satisfait.e<br>2. Un peu satisfait.e<br>3. Ni satisfait.e, ni insatisfait.e<br>4. Satisfait.e<br>5. Très satisfait.e<br>6. Ne veut pas divulguer

Deml MJ, *et al. BMJ Open* 2019; 9:e032218. doi: 10.1136/bmjopen-2019-032218

READ OUT ANSWER OPTIONS	8. missing	LIRE TOUTES LES REPONSES POSSIBLES	7. ne sait pas<br>8. missing
Wie zufrieden sind Sie mit den Diskussionen über Impfungen mit dem Arzt/ der Ärztin Ihres Kindes?<br><br>ANTWORTMÖGLICHKEITEN VORLESEN	1. überhaupt nicht zufrieden<br>2. einigermassen zufrieden<br>3. weder zufrieden noch unzufrieden<br>4. zufrieden<br>5. sehr zufrieden<br>6. möchte die Frage nicht beantworten<br>7. weiss nicht<br>8. keine Antwort	Quanto è soddisfatto/a delle discussioni che ha avuto sui vaccini con il medico di suo/a figlio/a?<br><br>LEGGERE LE RISPOSTE POSSIBILI	1. Per niente soddisfatto<br>2. In parte soddisfatto<br>3. Né soddisfatto né insoddisfatto<br>4. Soddisfatto<br>5. Molto soddisfatto<br>6. non vuole rivelare<br>7. non sa<br>8. mancante

Asked in:	✓ Childhood vaccinations

How satisfied were you with your discussions about vaccines with your teen`s doctor?<br><br>READ OUT ANSWER OPTIONS	9. Not at all satisfied<br>10. Somewhat satisfied<br>11. Neither satisfied nor unsatisfied<br>12. Satisfied<br>13. Very satisfied<br>14. doesn't want to disclose<br>15. doesn't know<br>16. missing	A quel point avez-vous été satisfait.e des discussions sur les vaccins avec le médecin de votre enfant?<br><br>LIRE TOUTES LES REPONSES POSSIBLES	9. Pas du tout satisfait.e<br>10. Un peu satisfait.e<br>11. Ni satisfait.e, ni insatisfait.e<br>12. Satisfait.e<br>13. Très satisfait.e<br>14. Ne veut pas divulguer<br>15. ne sait pas<br>16. missing
Wie zufrieden sind Sie mit den Diskussionen über Impfungen mit dem Arzt/ der Ärztin Ihres Kindes?<br><br>ANTWORTMÖGLICHKEITEN VORLESEN	9. überhaupt nicht zufrieden<br>10. einigermassen zufrieden<br>11. weder zufrieden noch unzufrieden<br>12. zufrieden<br>13. sehr zufrieden<br>14. möchte die Frage nicht beantworten<br>15. weiss nicht<br>16. keine Antwort	Quanto è soddisfatto/a delle discussioni che ha avuto sui vaccini con il medico di suo figlio/a?<br><br>LEGGERE LE RISPOSTE POSSIBILI	9. Per niente soddisfatto<br>10. In parte soddisfatto<br>11. Né soddisfatto né insoddisfatto<br>12. Soddisfatto<br>13. Molto soddisfatto<br>14. non vuole rivelare<br>15. non sa<br>16. mancante

Deml MJ, *et al. BMJ Open* 2019; 9:e032218. doi: 10.1136/bmjopen-2019-032218

Asked in:	✓ HPV parent

Q6

Please indicate how much you agree with the following statements:	Veuillez indiquer à quel point vous êtes d'accord ou en désaccord avec les énoncés suivants:
Bitte geben Sie an, wie sehr Sie den folgenden Aussagen zustimmen:	Per favore mi indichi quanto è d'accordo con le seguenti affermazioni:
Asked in:	✓ Childhood vaccinations<br>✓ HPV parent<br>✓ HPV adolescent

Q7

I am able to ask my child`s doctor questions about vaccination.<br><br>READ OUT ANSWER OPTIONS	1. Completely agree<br>2. Somewhat agree<br>3. Neither agree nor disagree<br>4. Somewhat disagree<br>5. Completely disagree<br>6. doesn't want to disclose<br>7. doesn't know<br>8. missing	Je suis capable de poser des questions sur la vaccination au médecin de mon enfant.<br><br>LIRE TOUTES LES REPONSES POSSIBLES	1. Tout à fait d'accord<br>2. Un peu d'accord<br>3. Ni en accord, ni en désaccord<br>4. Un peu en désacord<br>5. Pas du tout d'accord<br>6. ne veut pas divulguer<br>7. ne sait pas<br>8. missing
Ich kann dem Arzt/der Ärztin meines Kindes Fragen zu Impfungen stellen.<br><br>ANTWORTMÖGLI CHKEITEN VORLESEN	1. stimme stark zu<br>2. stimme einigermassen zu<br>3. weder noch<br>4. stimme eher nicht zu<br>5. stimme überhaupt nicht zu<br>6. möchte die Frage nicht beantworten<br>7. weiss nicht<br>8. keine Antwort	Sono capace di porre delle domande riguardanti le vaccinazioni al medico di mio/a figlio/a.<br><br>LEGGERE LE RISPOSTE POSSIBILI	1. Completam ente d'accordo<br>2. In parte d'accordo<br>3. Né d'accordo né in disaccordo<br>4. In parte in disaccordo<br>5. Completam ente in disaccordo<br>6. non vuole rivelare

			7. non sa<br>8. mancante
Asked in:	✓ Childhood vaccinations		

I am able to ask my teen`s doctor questions about vaccination.<br><br>READ OUT ANSWER OPTIONS	1. Completely agree<br>2. Somewhat agree<br>3. Neither agree nor disagree<br>4. Somewhat disagree<br>5. Completely disagree<br>6. doesn't want to disclose<br>7. doesn't know<br>8. missing	Je suis capable de poser des questions sur la vaccination au médecin de mon adolescent.<br><br>LIRE TOUTES LES REPONSES POSSIBLES	1. Tout à fait d'accord<br>2. Un peu d'accord<br>3. Ni en accord, ni en désaccord<br>4. Un peu en désacord<br>5. Pas du tout d'accord<br>6. ne veut pas divulguer<br>7. ne sait pas<br>8. missing
Ich kann dem Arzt/der Ärztin meines Kindes Fragen zu Impfungen stellen.<br><br>ANTWORTMÖGLICHKEITEN VORLESEN	1. stimme stark zu<br>2. stimme einigermassen zu<br>3. weder noch<br>4. stimme eher nicht zu<br>5. stimme überhaupt nicht zu<br>6. möchte die Frage nicht beantworten<br>7. weiss nicht<br>8. keine Antwort	Sono capace di porre delle domande riguardanti le vaccinazioni al medico di mio/a figlio/a.<br><br>LEGGERE LE RISPOSTE POSSIBILI	1. Completamente d'accordo<br>2. In parte d'accordo<br>3. Né d'accordo né in disaccordo<br>4. In parte in disaccordo<br>5. Completamente in disaccordo<br>6. non vuole rivelare<br>7. non sa<br>8. mancante
Asked in:	✓ HPV parent		

I am able to ask my doctor questions about vaccination.	1. Completely agree<br>2. Somewhat agree<br>3. Neither agree nor disagree<br>4. Somewhat disagree	Je suis capable de poser des questions sur la vaccination à mon médecin.	1. Tout à fait d'accord<br>2. Un peu d'accord<br>3. Ni en accord, ni en désaccord

Deml MJ, et al. BMJ Open 2019; 9:e032218. doi: 10.1136/bmjopen-2019-032218

READ OUT ANSWER OPTIONS	5. Completely disagree<br>6. doesn't want to disclose<br>7. doesn't know<br>8. missing	LIRE TOUTES LES REPONSES POSSIBLES	4. Un peu en désacord<br>5. Pas du tout d'accord<br>6. ne veut pas divulguer<br>7. ne sait pas<br>8. missing
Ich kann meinem Arzt/meiner Ärztin Fragen zu Impfungen stellen.<br><br>ANTWORTMÖGLICHKEITEN VORLESEN	1. stimme stark zu<br>2. stimme einigermassen zu<br>3. weder noch<br>4. stimme eher nicht zu<br>5. stimme überhaupt nicht zu<br>6. möchte die Frage nicht beantworten<br>7. weiss nicht<br>8. keine Antwort	Sono capace di porre delle domande riguardanti le vaccinazioni al medico di mio/a figlio/a.<br><br>LEGGERE LE RISPOSTE POSSIBILI	1. Completamente d'accordo<br>2. In parte d'accordo<br>3. Né d'accordo né in disaccordo<br>4. In parte in disaccordo<br>5. Completamente in disaccordo<br>6. non vuole rivelare<br>7. non sa<br>8. mancante
Asked in:	✓ HPV adolescent		

Q8

My child`s doctor takes the time needed to discuss my concerns about vaccination with me.<br><br>READ OUT ANSWER OPTIONS	1. Completely agree<br>2. Somewhat agree<br>3. Neither agree nor disagree<br>4. Somewhat disagree<br>5. Completely disagree<br>6. doesn't want to disclose<br>7. doesn't know<br>8. missing	Le médecin de mon enfant prend le temps nécessaire pour discuter de mes questions et inquiétudes sur la vaccnation.<br><br>LIRE TOUTES LES REPONSES POSSIBLES	1. Tout à fait d'accord<br>2. Un peu d'accord<br>3. Ni en accord, ni en désaccord<br>4. Un peu en désacord<br>5. Pas du tout d'accord<br>6. ne veut pas divulguer<br>7. ne sait pas<br>8. missing
Der Arzt/ die Ärztin meines Kindes nimmt sich die nötige Zeit um	1. stimme stark zu<br>2. stimme einigermassen zu	Il medico di mio/a figlio/a consacra il tempo necessario per discutere delle	1. Completamente d'accordo

Deml MJ, *et al. BMJ Open* 2019; 9:e032218. doi: 10.1136/bmjopen-2019-032218

meine Fragen und Sorgen bezüglich Impfungen mit mir zu besprechen.<br><br>ANTWORTMÖGLICHKEITEN VORLESEN	3. weder noch<br>4. stimme eher nicht zu<br>5. stimme überhaupt nicht zu<br>6. möchte die Frage nicht beantworten<br>7. weiss nicht<br>8. keine Antwort	mie preoccupazioni riguardanti le vaccinazioni.<br><br>LEGGERE LE RISPOSTE POSSIBILI	2. In parte d'accordo<br>3. Né d'accordo né in disaccordo<br>4. In parte in disaccordo<br>5. Completamente in disaccordo<br>6. non vuole rivelare<br>7. non sa<br>8. mancante
Asked in:	✓ Childhood vaccinations		

My teen`s doctor takes the time needed to discuss my concerns about vaccination with me.<br><br>READ OUT ANSWER OPTIONS	1. Completely agree<br>2. Somewhat agree<br>3. Neither agree nor disagree<br>4. Somewhat disagree<br>5. Completely disagree<br>6. doesn't want to disclose<br>7. doesn't know<br>8. missing	Le médecin de mon enfant prend le temps nécessaire pour discuter de mes questions et inquiétudes sur la vaccnation.<br><br>LIRE TOUTES LES REPONSES POSSIBLES	1. Tout à fait d'accord<br>2. Un peu d'accord<br>3. Ni en accord, ni en désaccord<br>4. Un peu en désacord<br>5. Pas du tout d'accord<br>6. ne veut pas divulguer<br>7. ne sait pas<br>8. missing
Der Arzt/ die Ärztin meines Kindes nimmt sich die nötige Zeit um meine Fragen und Sorgen bezüglich Impfungen mit mir zu besprechen.<br><br>ANTWORTMÖGLICHKEITEN VORLESEN	1. stimme stark zu<br>2. stimme einigermassen zu<br>3. weder noch<br>4. stimme eher nicht zu<br>5. stimme überhaupt nicht zu<br>6. möchte die Frage nicht beantworten<br>7. weiss nicht<br>8. keine Antwort	Il medico di mio/a figlio/a consacra il tempo necessario per discutere delle mie preoccupazioni riguardanti le vaccinazioni.<br><br>LEGGERE LE RISPOSTE POSSIBILI	1. Completamente d'accordo<br>2. In parte d'accordo<br>3. Né d'accordo né in disaccordo<br>4. In parte in disaccordo<br>5. Completamente in disaccordo<br>6. non vuole rivelare<br>7. non sa

Deml MJ, *et al. BMJ Open* 2019; 9:e032218. doi: 10.1136/bmjopen-2019-032218

			8. mancante
Asked in:	✓ HPV parent		

Q9

My child`s doctor takes my concerns about vaccination seriously.<br><br>READ OUT ANSWER OPTIONS	1. Completely agree<br>2. Somewhat agree<br>3. Neither agree nor disagree<br>4. Somewhat disagree<br>5. Completely disagree<br>6. doesn't want to disclose<br>7. doesn't know<br>8. missing	Le médecin de mon enfant prend mes inquiétudes sur la vaccination au sérieux.<br><br>LIRE TOUTES LES REPONSES POSSIBLES	1. Tout à fait d'accord<br>2. Un peu d'accord<br>3. Ni en accord, ni en désaccord<br>4. Un peu en désacord<br>5. Pas du tout d'accord<br>6. ne veut pas divulguer<br>7. ne sait pas<br>8. missing
Der Arzt/ die Ärztin meines Kindes nimmt meine Sorgen bezüglich Impfungen ernst.<br><br>ANTWORTMÖGLICHKEITEN VORLESEN	1. stimme stark zu<br>2. stimme einigermassen zu<br>3. weder noch<br>4. stimme eher nicht zu<br>5. stimme überhaupt nicht zu<br>6. möchte die Frage nicht beantworten<br>7. weiss nicht<br>8. keine Antwort	Il medico di mio/a figlio/a prende sul serio le mie preoccupazioni riguardanti le vaccinazioni.<br><br>LEGGERE LE RISPOSTE POSSIBILI	1. Completamente d'accordo<br>2. In parte d'accordo<br>3. Né d'accordo né in disaccordo<br>4. In parte in disaccordo<br>5. Completamente in disaccordo<br>6. non vuole rivelare<br>7. non sa<br>8. mancante
Asked in:	✓ Childhood vaccinations		

My teen`s doctor takes my concerns about vaccination seriously.	1. Completely agree<br>2. Somewhat agree<br>3. Neither agree nor disagree<br>4. Somewhat disagree	Le médecin de mon enfant prend mes inquiétudes sur la vaccination au sérieux.	1. Tout à fait d'accord<br>2. Un peu d'accord<br>3. Ni en accord, ni en désaccord

Deml MJ, *et al. BMJ Open* 2019; 9:e032218. doi: 10.1136/bmjopen-2019-032218

READ OUT ANSWER OPTIONS	5. Completely disagree<br>6. doesn't want to disclose<br>7. doesn't know<br>8. missing	LIRE TOUTES LES REPONSES POSSIBLES	4. Un peu en désacord<br>5. Pas du tout d'accord<br>6. ne veut pas divulguer<br>7. ne sait pas<br>8. missing
Der Arzt/ die Ärztin meines Kindes nimmt meine Sorgen bezüglich Impfungen ernst.<br><br>ANTWORTMÖGLICHKEITEN VORLESEN	1. stimme stark zu<br>2. stimme einigermassen zu<br>3. weder noch<br>4. stimme eher nicht zu<br>5. stimme überhaupt nicht zu<br>6. möchte die Frage nicht beantworten<br>7. weiss nicht<br>8. keine Antwort	Il medico di mio/a figlio/a prende sul serio le mie preoccupazioni riguardanti le vaccinazioni.<br><br>LEGGERE LE RISPOSTE POSSIBILI	1. Completamente d'accordo<br>2. In parte d'accordo<br>3. Né d'accordo né in disaccordo<br>4. In parte in disaccordo<br>5. Completamente in disaccordo<br>6. non vuole rivelare<br>7. non sa<br>8. mancante
Asked in:	✓ HPV parent		

Q10

My child`s doctor`s views on vaccination are similar to my own.<br><br>READ OUT ANSWER OPTIONS	1. Completely agree<br>2. Somewhat agree<br>3. Neither agree nor disagree<br>4. Somewhat disagree<br>5. Completely disagree<br>6. doesn't want to disclose<br>7. doesn't know<br>8. missing	Les points de vue du médecin de mon enfant au sujet de la vaccination sont similaires aux miens.<br><br>LIRE TOUTES LES REPONSES POSSIBLES	1. Tout à fait d'accord<br>2. Un peu d'accord<br>3. Ni en accord, ni en désaccord<br>4. Un peu en désacord<br>5. Pas du tout d'accord<br>6. ne veut pas divulguer<br>7. ne sait pas<br>8. missing
Die Ansichten des Arztes/ der Ärztin meines Kindes	1. stimme stark zu<br>2. stimme einigermassen zu	Il punto di vista del medico di mio/a figlio/a sulla	1. Completamente d'accordo

Deml MJ, *et al. BMJ Open* 2019; 9:e032218. doi: 10.1136/bmjopen-2019-032218

entsprechen meinen eigenen.<br><br>ANTWORTMÖGLICHKEITEN VORLESEN	3. weder noch<br>4. stimme eher nicht zu<br>5. stimme überhaupt nicht zu<br>6. möchte die Frage nicht beantworten<br>7. weiss nicht<br>8. keine Antwort	vaccinazione è simile al mio.<br><br>LEGGERE LE RISPOSTE POSSIBILI	2. In parte d'accordo<br>3. Né d'accordo né in disaccordo<br>4. In parte in disaccordo<br>5. Completamente in disaccordo<br>6. non vuole rivelare<br>7. non sa<br>8. mancante
Asked in:	✓ Childhood vaccinations		

My teen`s doctor`s views on vaccination are similar to my own.<br><br>READ OUT ANSWER OPTIONS	1. Completely agree<br>2. Somewhat agree<br>3. Neither agree nor disagree<br>4. Somewhat disagree<br>5. Completely disagree<br>6. doesn't want to disclose<br>7. doesn't know<br>8. missing	Les points de vue du médecin de mon enfant au sujet de la vaccination sont similaires aux miens.<br><br>LIRE TOUTES LES REPONSES POSSIBLES	1. Tout à fait d'accord<br>2. Un peu d'accord<br>3. Ni en accord, ni en désaccord<br>4. Un peu en désacord<br>5. Pas du tout d'accord<br>6. ne veut pas divulguer<br>7. ne sait pas<br>8. missing
Die Ansichten des Arztes/ der Ärztin meines Kindes entsprechen meinen eigenen.<br><br>ANTWORTMÖGLICHKEITEN VORLESEN	1. stimme stark zu<br>2. stimme einigermassen zu<br>3. weder noch<br>4. stimme eher nicht zu<br>5. stimme überhaupt nicht zu<br>6. möchte die Frage nicht beantworten<br>7. weiss nicht<br>8. keine Antwort	Il punto di vista del medico di mio/a figlio/a sulla vaccinazione è simile al mio.<br><br>LEGGERE LE RISPOSTE POSSIBILI	1. Completamente d'accordo<br>2. In parte d'accordo<br>3. Né d'accordo né in disaccordo<br>4. In parte in disaccordo<br>5. Completamente in disaccordo<br>6. non vuole rivelare

Deml MJ, *et al. BMJ Open* 2019; 9:e032218. doi: 10.1136/bmjopen-2019-032218

			7. non sa<br>8. mancante
Asked in:	✓ HPV parent		

Q11

Have you discussed vaccination for your child with any other doctor?	1. Yes<br>2. No<br>3. doesn't want to disclose<br>4. doesn't know<br>5. missing	Avez-vous déjà discuté de la vaccination pour votre enfant avec d`autres médecins?	1. Oui<br>2. Non<br>3. ne veut pas divulguer<br>4. ne sait pas<br>5. missing
Haben Sie je die Impfungen Impfung für Ihr Kind mit einem anderen Arzt/ einer anderen Ärztin besprochen?	1. ja<br>2. nein<br>3. möchte die Frage nicht beantworten<br>4. weiss nicht<br>5. keine Antwort	Le è mai capitato di discutere della vaccinazione infantile per suo/a figlio/a con un altro medico?	1. Si<br>2. No<br>3. non vuole rivelare<br>4. non sa<br>5. mancante
Asked in:	✓ Childhood vaccinations		

Have you discussed HPV vaccination for your teen with any other doctor?	1. Yes<br>2. No<br>3. doesn't want to disclose<br>4. doesn't know<br>5. missing	Avez-vous déjà discuté de la vaccination HPV pour votre enfant avec d`autres médecins?	1. Oui<br>2. Non<br>3. ne veut pas divulguer<br>4. ne sait pas<br>5. missing
Haben Sie je die die HPV Impfung Impfung für Ihr Kind mit einem anderen Arzt/ einer anderen Ärztin besprochen?	1. ja<br>2. nein<br>3. möchte die Frage nicht beantworten<br>4. weiss nicht<br>5. keine Antwort	Le è mai capitato di discutere della vaccinazione contro l`HPV per suo/a figlio/a con un altro medico?	1. Si<br>2. No<br>3. non vuole rivelare<br>4. non sa<br>5. mancante
Asked in:	✓ HPV parent		

If Q11 = 1, ask Q12-14

Q12

What led you to consult another doctor?<br><br>READ OUT ANSWER OPTIONS	1. Second opinion<br>2. moved<br>3. former provider stopped working<br>4. disagreement with provider<br>5. Other:_______<br>6. doesn't want to disclose<br>7. doesn't know<br>8. missing	Pourquoi est-ce que vous avez consulté un autre médecin?<br><br>LIRE TOUTES LES REPONSES POSSIBLES	1. Deuxième opinion<br>2. a changé de résidence<br>3. l'ancien professionnel de la santé a arrêté de travailler<br>4. Différence d'opinion ou désaccord avec le professionnel de la santé<br>5. Autre:<br>6. ne veut pas divulguer<br>7. ne sait pas<br>8. missing
Was hat Sie dazu bewogen, einen anderen Arzt/ eine andere Ärztin aufzusuchen ?<br><br>ANTWORTMÖGLICHKEITEN VORLESEN	1. Zweitmeinung<br>2. Umgezogen<br>3. Früherer Arzt/Ärztin praktiziert nicht mehr<br>4. Uneinigkeit mit Arzt/Ärztin<br>5. Anderes:_____<br>6. möchte die Frage nicht beantworten<br>7. weiss nicht<br>8. keine Antwort	Che cosa l'ha portata a consultare un altro medico?<br><br>LEGGERE LE RISPOSTE POSSIBILI	1. Secondo parere<br>2. spostato<br>3. Il precedente medico/operatore della salute ha smesso di lavorare<br>4. Disaccordo con il medico/operatore della salute<br>5. Altro:_____<br>6. non vuole rivelare<br>7. non sa<br>8. mancante
Asked in:	✓ Childhood vaccinations<br>✓ HPV parent		

Q13

How satisfied were you with your	1. Not at all satisfied	A quel point étiez-vous satisfait.e de	1. Pas du tout satisfait.e

discussions about vaccines with that doctor?<br><br>READ OUT ANSWER OPTIONS	2. Somewhat satisfied<br>3. Neither satisfied nor unsatisfied<br>4. Satisfied<br>5. Very satisfied<br>6. doesn't want to disclose<br>7. doesn't know<br>8. missing	vos discussions sur les vaccins avec ce médecin?<br><br>LIRE TOUTES LES REPONSES POSSIBLES	2. Un peu satisfait.e<br>3. Ni satisfait.e, ni insatisfait.e<br>4. Satisfait.e<br>5. Très satisfait.e<br>6. Ne veut pas divulguer<br>7. ne sait pas<br>8. missing
Wie zufrieden waren Sie mit der Besprechung über Impfungen mit diesem Arzt/ dieser Ärztin?<br><br>ANTWORTMÖGLICHKEITEN VORLESEN	1. überhaupt nicht zufrieden<br>2. einigermassen zufrieden<br>3. weder zufrieden noch unzufrieden<br>4. zufrieden<br>5. sehr zufrieden<br>6. möchte die Frage nicht beantworten<br>7. weiss nicht<br>8. keine Antwort	Quanto è soddisfatto/a delle discussioni che ha avuto sul vaccino sulla vaccinazione infantile con quest`altro medico?<br><br>LEGGERE LE RISPOSTE POSSIBILI	1. überhaupt nicht zufrieden<br>2. einigermassen zufrieden<br>3. weder zufrieden noch unzufrieden<br>4. zufrieden<br>5. sehr zufrieden<br>6. möchte die Frage nicht beantworten<br>7. weiss nicht<br>8. keine Antwort
Asked in:		✓ Childhood vaccinations	

How satisfied were you with your discussions about HPV vaccines with that doctor?<br><br>READ OUT ANSWER OPTIONS	1. Not at all satisfied<br>2. Somewhat satisfied<br>3. Neither satisfied nor unsatisfied<br>4. Satisfied<br>5. Very satisfied<br>6. doesn't want to disclose<br>7. doesn't know<br>8. missing	A quel point étiez-vous satisfait.e de vos discussions sur les vaccins HPV avec ce médecin?<br><br>LIRE TOUTES LES REPONSES POSSIBLES	1. Pas du tout satisfait.e<br>2. Un peu satisfait.e<br>3. Ni satisfait.e, ni insatisfait.e<br>4. Satisfait.e<br>5. Très satisfait.e<br>6. Ne veut pas divulguer<br>7. ne sait pas<br>8. missing
Wie zufrieden waren Sie mit der	1. überhaupt nicht zufrieden	Quanto è soddisfatto/a delle	1. überhaupt nicht zufrieden

Deml MJ, *et al. BMJ Open* 2019; 9:e032218. doi: 10.1136/bmjopen-2019-032218

Besprechung über HPV Impfungen mit diesem Arzt/ dieser Ärztin?<br><br>ANTWORTMÖGLICHKEITEN VORLESEN	2. einigermassen zufrieden<br>3. weder zufrieden noch unzufrieden<br>4. zufrieden<br>5. sehr zufrieden<br>6. möchte die Frage nicht beantworten<br>7. weiss nicht<br>8. keine Antwort	discussioni che ha avuto sul vaccino sul vaccino contro l`HPV con quest`altro medico?<br><br>LEGGERE LE RISPOSTE POSSIBILI	2. einigermassen zufrieden<br>3. weder zufrieden noch unzufrieden<br>4. zufrieden<br>5. sehr zufrieden<br>6. möchte die Frage nicht beantworten<br>7. weiss nicht<br>8. keine Antwort

Asked in:	✓ HPV parent

Q14

How much do you trust the information that doctor gave you about vaccines?<br><br>READ OUT ANSWER OPTIONS	1. Completely trust<br>2. Somewhat trust<br>3. Neither trust nor distrust<br>4. Somewhat distrust<br>5. Don't trust at all<br>6. doesn't want to disclose<br>7. doesn't know<br>8. missing	A quel point faites-vous confiance aux informations au sujet des vaccins que ce médecin vous a données?<br><br>LIRE TOUTES LES REPONSES POSSIBLES	1. Fait entièrement confiance<br>2. Fait un peu confiance<br>3. Ne fait ni confiance, ni ne fait pas confiance<br>4. Ne fait pas vraiment confiance<br>5. ne fait pas du tout confiance<br>6. ne veut pas divulguer<br>7. ne sait pas<br>8. missing
Wie sehr haben Sie der Information zu Impfungenvertraut, die dieser Arzt/ diese Ärztin Ihnen gegeben hat?	1. Ich habe grosses Vertrauen<br>2. Ich habe etwas Vertrauen<br>3. Weder Vertrauen noch Misstrauen<br>4. Ich habe etwas Misstrauen	Quanto si fida delle informazioni che questo medico le ha dato?<br><br>LEGGERE LE RISPOSTE POSSIBILI	1. Si fida completamente<br>2. Si fida in parte<br>3. Né si fida né diffida<br>4. Diffida in parte

Deml MJ, *et al. BMJ Open* 2019; 9:e032218. doi: 10.1136/bmjopen-2019-032218

ANTWORTMÖGLICHKEITEN VORLESEN	5. Ich habe gar kein Vertrauen<br>6. möchte die Frage nicht beantworten<br>7. weiss nicht<br>8. keine Antwort		5. Diffida completamente<br>6. non vuole rivelare<br>7. non sa<br>8. mancante
Asked in:	✓ Childhood vaccinations<br>✓ HPV parent		

Theme 2: Information sources consulted in vaccination decision-making

Q1

What are your most trusted information sources on vaccination?<br><br>MULTIPLE OPTIONS, DO NOT READ OUT LOUD	1. no information/no source<br>2. Family<br>3. my child's doctor<br>4. other health professional<br>5. Friends and acquaintances<br>6. Public health authorities<br>7. TV<br>8. Internet searches<br>9. Social media (such as Facebook, Instagram and Twitter)<br>10. Print media (such as books, magazines and newspapers)<br>11. Other:__________<br>12. doesn't want to disclose<br>13. doesn't know<br>14. missing	Quelles sont vos sources d'informations les plus fiables sur la vaccination?<br><br>PLUSIEURS OPTIONS, NE PAS LIRE TOUTES LES REPONSES POSSIBLES	1. aucune information/ aucune source<br>2. famille<br>3. le médecin de mon enfant<br>4. un autre profesionnel de la santé<br>5. Des ami.e.s ou connaissances<br>6. Les autorités de la santé publique (p. ex. L'Office fédéral de la santé publique)<br>7. La télévision<br>8. Des recherches sur Internet<br>9. Les réseaux sociaux (tels que Facebook, Instagram et Twitter)<br>10. la presse écrite (p.

			ex. livres, magazines et journaux)<br>11. Autre:__________<br>12. ne veut pas divulguer<br>13. ne sait pas<br>14. missing
Was sind Ihre zuverlässigsten Informationsquellen zur Impfung?<br><br>VERSCHIEDENE OPTIONEN- NICHT LAUT VORLESEN	1. Keine Informationsquelle<br>2. Familie<br>3. Arzt/Ärztin meines Kindes<br>4. Andere Gesundheitsfachperson<br>5. Freunde und Bekannte<br>6. Gesundheitsbehörden<br>7. TV<br>8. Internet-Recherchen<br>9. Soziale Medien (wie z.B. Facebook, Instagram, Twitter)<br>10. Print Medien (wie z.B. Zeitungen, Broschüren)<br>11. Anderes:__________<br>12. möchte die Frage nicht beantworten<br>13. weiss nicht<br>14. keine Antwort	Quali sono le fonti più affidabili di informazioni sulla vaccinazione?<br><br>NON LEGGERE LE RISPOSTE POSSIBILI	1. nessuna informazione/ nessuna fonte<br>2. Famiglia<br>3. il medico di mio figlio<br>4. altro operatore della salute<br>5. Amici e conoscenti<br>6. Autorità della salute pubblica<br>7. TV<br>8. Ricerche internet<br>9. Social media (come Facebook, Instagram e Twitter)<br>10. Media stampati (come libri, riviste o giornali)<br>11. Altro:__________<br>12. non vuole rivelare<br>13. non sa<br>14. mancante

Asked in:	✓ Childhood vaccinations<br>✓ HPV parent<br>✓ HPV adolescent

If Q1 = 7, ask Q2

Deml MJ, *et al. BMJ Open* 2019; 9:e032218. doi: 10.1136/bmjopen-2019-032218

Q2

Which TV programs?<br><br>DO NOT READ OUT UNLESS NECESSARY		Quels programmes télévisés?<br><br>DEMANDEZ A HAUTE VOIX UNIQUEMENT SI NECESSAIRE	
Welche Fernsehprogramm e?<br><br>NICHT LAUT VORLESEN, WENN SIE NICHT ERFORDERLICH SIND		Quali programmi televisivi?<br><br>NON LEGGERE A MENO CHE NECESSARIO	

Asked in:	✓ Childhood vaccination<br>✓ HPV parent<br>✓ HPV adolescent

If Q1 = 8, ask Q3

Q3

Which websites?<br><br>DO NOT READ OUT UNLESS NECESSARY		Quels sites Web?<br><br>DEMANDEZ A HAUTE VOIX UNIQUEMENT SI NECESSAIRE	
Welches Websites?<br><br>NICHT LAUT VORLESEN, WENN SIE NICHT ERFORDERLICH SIND		Quali siti web?<br><br>NON LEGGERE A MENO CHE NECESSARIO	

Asked in:	✓ Childhood vaccination<br>✓ HPV parent<br>✓ HPV adolescent

If Q1 = 9, ask Q4

Q4

What social media?<br><br>DO NOT READ OUT UNLESS NECESSARY		Quels reseaux sociaux?<br><br>DEMANDEZ A HAUTE VOIX UNIQUEMENT SI NECESSAIRE	
Welche Sozial Medien?<br><br>NICHT LAUT VORLESEN, WENN SIE NICHT ERFORDERLICH SIND		Quali Social Media?<br><br>NON LEGGERE A MENO CHE NECESSARIO	

Asked in:	✓ Childhood vaccination<br>✓ HPV parent<br>✓ HPV adolescent

If Q1 = 10, ask Q5

Q5

What print media?<br><br>DO NOT READ OUT UNLESS NECESSARY		Quelles presses écrites?<br><br>DEMANDEZ A HAUTE VOIX UNIQUEMENT SI NECESSAIRE	
Welche Print Medien?		Quali media stampati?	

Deml MJ, *et al. BMJ Open* 2019; 9:e032218. doi: 10.1136/bmjopen-2019-032218

NICHT LAUT VORLESEN, WENN SIE NICHT ERFORDERLICH SIND		NON LEGGERE A MENO CHE NECESSARIO	
Asked in:	✓ Childhood vaccination<br>✓ HPV parent<br>✓ HPV adolescent		

If Q8 = 11, ask Q6

Q6

What other sources?<br><br>DO NOT READ OUT UNLESS NECESSARY		Quelles autres sources?<br><br>DEMANDEZ A HAUTE VOIX UNIQUEMENT SI NECESSAIRE	
Welche anderen Quellen?<br><br>NICHT LAUT VORLESEN, WENN SIE NICHT ERFORDERLICH SIND		Quali altre fonti?<br><br>NON LEGGERE A MENO CHE NECESSARIO	
Asked in:	✓ Childhood vaccination<br>✓ HPV parent<br>✓ HPV adolescent		

Q7

Did you apply the information you received when making decisions about vaccination (for your child)?	1. Yes<br>2. No<br>3. doesn't want to disclose<br>4. doesn't know<br>5. missing	Avez-vous considéré ces informations en prenant votre décision de vaccination (pour votre enfant?)	1. Oui<br>2. Non<br>3. ne veut pas divulguer<br>4. ne sait pas<br>5. missing

Deml MJ, et al. BMJ Open 2019; 9:e032218. doi: 10.1136/bmjopen-2019-032218

Hatten die erhaltenen Informationen Einfluss auf die Entscheidungen, welche sie über die Impfung (Ihres Kindes) gefällt haben?	1. ja<br>2. nein<br>3. möchte die Frage nicht beantworten<br>4. weiss nicht<br>5. keine Antwort	Ha considerato le informazioni che ha ricevuto quando si è trattato di decidere se vaccinare (suo/a figlio/a)?	1. Si<br>2. No<br>3. non vuole rivelare<br>4. non sa<br>5. mancante
Asked in:		✓ Childhood vaccinations<br>✓ HPV parent<br>✓ HPV adolescent	

Theme 3: Perceptions about risk and control concerning possible exposure to vaccine-preventable diseases

Q1

Please indicate how much you agree with the following statements:	Veuillez indiquer à quel point vous êtes d'accord ou en désaccord avec les énoncés suivants:
Bitte geben Sie an wie sehr Sie den folgenden Aussagen zustimmen:	Per favore indichi quanto è d'accordo con le seguenti affermazioni:
Asked in:	✓ Childhood vaccinations

Q2

I can always prevent my child from being infected with vaccine-preventable diseases by other means than vaccination.<br><br>READ OUT ANSWER OPTIONS	1. strongly agree<br>2. agree<br>3. neither agree nor disagree<br>4. disagree<br>5. Strongly disagree<br>6. doesn't want to disclose<br>7. doesn't know<br>8. missing	Je peux toujours prévenir que mon enfant soit infecté par des maladies pouvant être prévenues par un vaccin par autres moyens que de la vaccination.<br><br>LIRE TOUTES LES REPONSES POSSIBLES	1. Tout à fait d'accord<br>2. D'accord<br>3. Ni en accord, ni en désaccord<br>4. pas d'accord<br>5. Pas du tout d'accord<br>6. ne veut pas divulguer<br>7. ne sait pas<br>8. missing
Ich kann jederzeit verhindern, dass mein Kind sich mit	1. stimme stark zu<br>2. stimme zu<br>3. weder noch	Posso sempre prevenire che mio/a figlio/a si	1. Fortemente d'accordo<br>2. d'accordo

Deml MJ, *et al. BMJ Open* 2019; 9:e032218. doi: 10.1136/bmjopen-2019-032218

Krankheiten ansteckt, vor denen Impfungen schützen würden.<br><br>ANTWORTMÖGLICHKEITEN VORLESEN	4. stimme nicht zu<br>5. stimme gar nicht zu<br>6. möchte die Frage nicht beantworten<br>7. weiss nicht<br>8. keine Antwort	infetti con malattie prevenibili tramite vaccino.<br><br>LEGGERE LE RISPOSTE POSSIBILI	3. né d'accordo né in disaccordo<br>4. in disaccordo<br>5. Fortemente in disaccordo<br>6. non vuole rivelare<br>7. non sa<br>8. mancante
Asked in:		✓ Childhood vaccinations	

Q3

Vaccine-preventable diseases can be easily cured in Switzerland.<br><br>READ OUT ANSWER OPTIONS	1. strongly agree<br>2. agree<br>3. neither agree nor disagree<br>4. disagree<br>5. Strongly disagree<br>6. doesn't want to disclose<br>7. doesn't know<br>8. missing	En Suisse, on peut facilement guérir d'une maladie pouvant être prévenue par un vaccin.<br><br>LIRE TOUTES LES REPONSES POSSIBLES	1. Tout à fait d'accord<br>2. D'accord<br>3. Ni en accord, ni en désaccord<br>4. pas d'accord<br>5. Pas du tout d'accord<br>6. ne veut pas divulguer<br>7. ne sait pas<br>8. missing
Krankheiten, vor denen Impfungen schützen würden, können in der Schweiz einfach geheilt werden.<br><br>ANTWORTMÖGLICHKEITEN VORLESEN	1. stimme stark zu<br>2. stimme zu<br>3. weder noch<br>4. stimme nicht zu<br>5. stimme gar nicht zu<br>6. möchte die Frage nicht beantworten<br>7. weiss nicht<br>8. keine Antwort	In Svizzera, le malattie prevenibili tramite vaccino possono essere facilemente curate.<br><br>LEGGERE LE RISPOSTE POSSIBILI	1. Fortemente d'accordo<br>2. d'accordo<br>3. né d'accordo né in disaccordo<br>4. in disaccordo<br>5. Fortemente in disaccordo<br>6. non vuole rivelare<br>7. non sa<br>8. mancante
Asked in:		✓ Childhood vaccinations	

Q4

Vaccines can cause serious	1. strongly agree<br>2. agree	Les vaccins peuvent causer des problèmes	1. Tout à fait d'accord<br>2. D'accord

Deml MJ, *et al. BMJ Open* 2019; 9:e032218. doi: 10.1136/bmjopen-2019-032218

long-term harm to health.<br><br>READ OUT ANSWER OPTIONS	3. neither agree nor disagree<br>4. disagree<br>5. Strongly disagree<br>6. doesn't want to disclose<br>7. doesn't know<br>8. missing	sévères de santé à long-terme.<br><br>LIRE TOUTES LES REPONSES POSSIBLES	3. Ni en accord, ni en désaccord<br>4. pas d'accord<br>5. Pas du tout d'accord<br>6. ne veut pas divulguer<br>7. ne sait pas<br>8. missing
Impfungen können ernsthafte Langzeitschäden auslösen.<br><br>ANTWORTMÖGLICHKEITEN VORLESEN	1. stimme stark zu<br>2. stimme zu<br>3. weder noch<br>4. stimme nicht zu<br>5. stimme gar nicht zu<br>6. möchte die Frage nicht beantworten<br>7. weiss nicht<br>8. keine Antwort	I vaccini possono avere conseguenze gravi a lungo termine sulla salute.<br><br>LEGGERE LE RISPOSTE POSSIBILI	1. Fortemente d'accordo<br>2. d'accordo<br>3. né d'accordo né in disaccordo<br>4. in disaccordo<br>5. Fortemente in disaccordo<br>6. non vuole rivelare<br>7. non sa<br>8. mancante

Asked in:	✓ Childhood vaccinations

Q5

Vaccination is unnatural, so it is best to vaccinate as little as possible.<br><br>READ OUT ANSWER OPTIONS	1. strongly agree<br>2. agree<br>3. neither agree nor disagree<br>4. disagree<br>5. Strongly disagree<br>6. doesn't want to disclose<br>7. doesn't know<br>8. missing	La vaccination n'est pas naturelle, donc, il est préférable de vacciner le moins possible.<br><br>LIRE TOUTES LES REPONSES POSSIBLES	1. Tout à fait d'accord<br>2. D'accord<br>3. Ni en accord, ni en désaccord<br>4. pas d'accord<br>5. Pas du tout d'accord<br>6. ne veut pas divulguer<br>7. ne sait pas<br>8. missing
Impfen ist unnatürlich, deshalb impfe ich so wenig wie möglich.	1. stimme stark zu<br>2. stimme zu<br>3. weder noch<br>4. stimme nicht zu<br>5. stimme gar nicht zu<br>6. möchte die Frage nicht beantworten	La vaccinazione è innaturale, dunque è meglio vaccinare il meno possibile.	1. Fortemente d'accordo<br>2. d'accordo<br>3. né d'accordo né in disaccordo<br>4. in disaccordo<br>5. Fortemente in disaccordo

Deml MJ, *et al. BMJ Open* 2019; 9:e032218. doi: 10.1136/bmjopen-2019-032218

ANTWORTMÖGLICHKEITEN VORLESEN	7. weiss nicht<br>8. keine Antwort	LEGGERE LE RISPOSTE POSSIBILI	6. non vuole rivelare<br>7. non sa<br>8. mancante
Asked in:		✓ Childhood vaccinations	

Q6

How likely do you think it is that your child will be exposed to vaccine-preventable diseases in your home?<br><br>READ OUT ANSWER OPTIONS	1. Very likely<br>2. Somewhat likely<br>3. Not sure<br>4. Somewhat unlikely<br>5. Very unlikely<br>6. doesn't want to disclose<br>7. doesn't know<br>8. missing	Selon vous, quelle est la probabilité que votre enfant soit exposé aux maladies pouvant être prévenues par un vaccin au sein de votre ménage?<br><br>LIRE TOUTES LES REPONSES POSSIBLES	1. Très probable<br>2. Un peu probable<br>3. Pas sûr.e<br>4. Un peu improbable<br>5. Très improbable<br>6. ne veut pas divulguer<br>7. ne sait pas<br>8. missing
Für wie wahrscheinlich halten Sie es, dass Ihr Kind bei Ihnen zuhause Krankheiten ausgesetzt sein wird, vor denen Impfungen schützen würden ?<br><br>ANTWORTMÖGLICHKEITEN VORLESEN	1. sehr wahrscheinlich<br>2. eher wahrscheinlich<br>3. unsicher<br>4. eher unwahrscheinlich<br>5. sehr unwahrscheinlich<br>6. möchte die Frage nicht beantworten<br>7. weiss nicht<br>8. keine Antwort	Quanto pensa sia probabile che suo/a figlio/a sia esposto/a a malattie prevenibili tramite vaccino in casa sua?<br><br>LEGGERE LE RISPOSTE POSSIBILI	1. Molto probabile<br>2. Parzialmente probabile<br>3. Non sicuro<br>4. Parzialmente improbabile<br>5. Molto improbabile<br>6. non vuole rivelare<br>7. non vuole rivelare<br>8. mancante
Asked in:		✓ Childhood vaccinations	

Q7

How likely do you think it is that your child will be exposed to	1. Very likely<br>2. Somewhat likely<br>3. Not sure	Selon vous, quelle est la probabilité que votre enfant soit exposé aux	1. Très probable<br>2. Un peu probable<br>3. Pas sûr.e

Deml MJ, *et al. BMJ Open* 2019; 9:e032218. doi: 10.1136/bmjopen-2019-032218

vaccine-preventable diseases in your community?<br><br>READ OUT ANSWER OPTIONS	4. Somewhat unlikely<br>5. Very unlikely<br>6. doesn't want to disclose<br>7. doesn't know<br>8. missing	maladies pouvant être prévenues par un vaccin au sein de votre communauté?<br><br>LIRE TOUTES LES REPONSES POSSIBLES	4. Un peu improbable<br>5. Très improbable<br>6. ne veut pas divulguer<br>7. ne sait pas<br>8. missing
Für wie wahrscheinlich halten Sie es dass Ihr Kind in Ihrem sozialen Umfeld Krankheiten ausgesetzt sein wird, vor denen Impfungen schützen würden ?<br><br>ANTWORTMÖGLICHKEITEN VORLESEN	1. sehr wahrscheinlich<br>2. eher wahrscheinlich<br>3. unsicher<br>4. eher unwahrscheinlich<br>5. sehr unwahrscheinlich<br>6. möchte die Frage nicht beantworten<br>7. weiss nicht<br>8. keine Antwort	Quanto pensa sia probabile che suo/a figlio/a sia esposto/a a malattie prevenibili tramite vaccino nella sua comunità?<br><br>LEGGERE LE RISPOSTE POSSIBILI	1. Molto probabile<br>2. Parzialmente probabile<br>3. Non sicuro<br>4. Parzialmente improbabile<br>5. Molto improbabile<br>6. non vuole rivelare<br>7. non vuole rivelare<br>8. mancante

Asked in:	✓ Childhood vaccinations

Q8

About how many of your family members with children do you think have vaccinated their children?<br><br>READ OUT ANSWER OPTIONS	1. Almost all<br>2. about three-quarters<br>3. about half<br>4. about a quarter<br>5. almost none<br>6. doesn't want to disclose<br>7. doesn't know<br>8. missing	D'après vous, à peu près combien des membres de votre famille avec des enfants ont fait vacciner leurs enfants?<br><br>LIRE TOUTES LES REPONSES POSSIBLES	1. Presque tous<br>2. Environ trois quarts<br>3. Environ la moitié<br>4. Environ un quart<br>5. Presqu'aucun<br>6. ne veut pas divulguer<br>7. ne sait pas<br>8. missing
Was denken Sie, Wie viele Ihrer Familienmitglieder	1. fast alle<br>2. etwa drei Viertel	Quanti membri della sua famiglia con figli pensa	1. Quasi tutto<br>2. circa tre quarti<br>3. circa la metà

Deml MJ, *et al*. *BMJ Open* 2019; 9:e032218. doi: 10.1136/bmjopen-2019-032218

mit Kindern haben ihre Kinder geimpft?<br><br>ANTWORTMÖGLICHKEITEN VORLESEN	3. etwa die Hälfte<br>4. etwa ein Viertel<br>5. fast niemand<br>6. möchte die Frage nicht beantworten<br>7. weiss nicht<br>8. keine Antwort	abbiano vaccinato i loro figli?<br><br>LEGGERE LE RISPOSTE POSSIBILI	4. circa un quarto<br>5. quasi nessuno<br>6. non vuole rivelare<br>7. non sa<br>8. mancante
Asked in:		✓ Childhood vaccinations	

Q9

About how many of your friends with children do you think have vaccinated their children?<br><br>READ OUT ANSWER OPTIONS	1. Almost all<br>2. about three-quarters<br>3. about half<br>4. about a quarter<br>5. almost none<br>6. doesn't want to disclose<br>7. doesn't know<br>8. missing	D'après vous, à peu près combien de vos amis ont fait vacciner leurs enfants?<br><br>LIRE TOUTES LES REPONSES POSSIBLES	1. Presque tous<br>2. Environ trois quarts<br>3. Environ la moitié<br>4. Environ un quart<br>5. Presqu'aucun<br>6. ne veut pas divulguer<br>7. ne sait pas<br>8. missing
Was denken Sie, Wie viele Ihrer Freunde mit Kindern haben Ihre Kinder geimpft?<br><br>ANTWORTMÖGLICHKEITEN VORLESEN	1. fast alle<br>2. etwa drei Viertel<br>3. etwa die Hälfte<br>4. etwa ein Viertel<br>5. fast niemand<br>6. möchte die Frage nicht beantworten<br>7. weiss nicht<br>8. keine Antwort	Quanti dei suoi amici con figli pensa abbiano vaccinato i loro figli?<br><br>LEGGERE LE RISPOSTE POSSIBILI	1. Quasi tutto<br>2. circa tre quarti<br>3. circa la metà<br>4. circa un quarto<br>5. quasi nessuno<br>6. non vuole rivelare<br>7. non sa<br>8. mancante
Asked in:		✓ Childhood vaccinations	

Q10

About how many of the children in your community do you think are vaccinated?	1. Almost all<br>2. about three-quarters<br>3. about half<br>4. about a quarter<br>5. almost none	D'après vous, a peu près combien des enfants au sein de votre	1. Presque tous<br>2. Environ trois quarts<br>3. Environ la moitié<br>4. Environ un quart

Deml MJ, *et al. BMJ Open* 2019; 9:e032218. doi: 10.1136/bmjopen-2019-032218

READ OUT ANSWER OPTIONS	6. doesn't want to disclose<br>7. doesn't know<br>8. missing	communauté sont vaccinés?<br><br>LIRE TOUTES LES REPONSES POSSIBLES	5. Presqu'aucun<br>6. ne veut pas divulguer<br>7. ne sait pas<br>8. missing
Was denken Sie, Wie viele Kinder in Ihrem sozialen Umfeld sind geimpft?<br><br>ANTWORTMÖGLICHKEITEN VORLESEN	1. fast alle<br>2. etwa drei Viertel<br>3. etwa die Hälfte<br>4. etwa ein Viertel<br>5. fast niemand<br>6. möchte die Frage nicht beantworten<br>7. weiss nicht<br>8. keine Antwort	Quanti bambini della sua comunità pensa siano vaccinati?<br><br>LEGGERE LE RISPOSTE POSSIBILI	1. Quasi tutto<br>2. circa tre quarti<br>3. circa la metà<br>4. circa un quarto<br>5. quasi nessuno<br>6. non vuole rivelare<br>7. non sa<br>8. mancante
Asked in:	✓ Childhood vaccinations		

Theme 3: HPV specific questions

Q1

Now I want to ask you some questions about the HPV vaccine.	J'aimerais maintenant vous poser quelques questions sur le vaccin HPV.
Jetzt würde ich Ihnen gerne einige Fragen zur HPV Impfung stellen.	Ora vorrei porle alcune domande riguardo il vaccino contro l'HPV.
Asked in:	✓ HPV parent<br>✓ HPV adolescent

Q2

Have you heard of the HPV vaccine?	1. yes<br>2. no<br>3. doesn't want to disclose<br>4. doesn't know<br>5. missing	Avez-vous déjà entendu parler du vaccin HPV?	1. oui<br>2. non<br>3. ne veut pas divulguer<br>4. ne sait pas<br>5. missing
Haben Sie schon von der HPV Impfung gehört?	1. ja<br>2. nein<br>3. möchte die Frage nicht beantworten<br>4. weiss nicht<br>5. keine Antwort	Ha già sentito parlare del vaccino contro l'HPV?	1. si<br>2. no<br>3. non vuole rivelare<br>4. non sa<br>5. mancante

Deml MJ, *et al. BMJ Open* 2019; 9:e032218. doi: 10.1136/bmjopen-2019-032218

Asked in:	✓ HPV parent<br>✓ HPV adolescent

If Q2 = 1, then ask Q3

Q3

What is it for?<br><br>PROBE:<br>ANYTHING ELSE?		Pour quelles raisons ce vaccin est-il administré?<br><br>PROBER: D'autres choses?	
Wozu ist sie gut?<br><br>VERSUCHE:<br>NOCH ZU ETWAS ANDEREM		A cosa serve?<br><br>RILANCIO:<br>QUALCOS'ALTRO?	

Asked in:	✓ HPV parent<br>✓ HPV adolescent

If Q2 = 2, then ask Q4

Q4

Have you heard of the cervical cancer vaccine?	1. yes<br>2. no<br>3. doesn't want to disclose<br>4. doesn't know<br>5. missing	Avez-vous déjà entendu parler du vaccin contre les cancers du col de l'utérus?	1. oui<br>2. non<br>3. ne veut pas divulguer<br>4. ne sait pas<br>5. missing
Haben Sie schon von der Impfung gegen Gebärmutterhalskrebs gehört?	1. ja<br>2. nein<br>3. möchte die Frage nicht beantworten<br>4. weiss nicht<br>5. keine Antwort	Ha già sentito parlare del vaccino contro il cancro della cervice uterina?	1. si<br>2. no<br>3. non vuole rivelare<br>4. non sa<br>5. mancante

Asked in:	✓ HPV parent<br>✓ HPV adolescent

If Q2 or Q4 = 1, ask Q5-7

Deml MJ, *et al. BMJ Open* 2019; 9:e032218. doi: 10.1136/bmjopen-2019-032218

Q5

Has your teen received HPV vaccine?	1. Yes<br>2. no<br>3. doesn't want to disclose<br>4. doesn't know<br>5. missing	Votre enfant, a-t-il.elle reçu le vaccin HPV?	1. oui<br>2. non<br>3. ne veut pas divulguer<br>4. ne sait pas<br>5. missing
Hat Ihr Kind als Jugendliche/r die HPV Impfung erhalten?	1. ja<br>2. nein<br>3. möchte die Frage nicht beantworten<br>4. weiss nicht<br>5. keine Antwort	Suo/a figlio/a ha ricevuto il vaccino contro l`HPV?	1. si<br>2. no<br>3. non vuole rivelare<br>4. non sa<br>5. mancante

Asked in:	✓ HPV parent

Have you received HPV vaccine?	1. yes<br>2. no<br>3. doesn't want to disclose<br>4. doesn't know<br>5. missing	Avez-vous reçu le vaccin HPV?	1. oui<br>2. non<br>3. ne veut pas divulguer<br>4. ne sait pas<br>5. missing
Haben Sie die HPV Impfung erhalten?	1. ja<br>2. nein<br>3. möchte die Frage nicht beantworten<br>4. weiss nicht<br>5. keine Antwort	Ha ricevuto il vaccino contro l`HPV?	1. si<br>2. no<br>3. non vuole rivelare<br>4. non sa<br>5. mancante

Asked in:	✓ HPV adolescent

If Q5 = 1, ask Q6

Q6

Where did your teen receive HPV vaccine?		Où est-ce que votre enfant a reçu le vaccin HPV?	
Wo hat Ihr Kind als Jugendliche/r die HPV Impfung bekommmen?		Dove ha ricevuto suo/a figlio/a il vaccino contro l`HPV?	

Asked in:	✓ HPV parent

Deml MJ, *et al. BMJ Open* 2019; 9:e032218. doi: 10.1136/bmjopen-2019-032218

Where did you receive HPV vaccine?		Où est-ce que vous avez reçu le vaccin HPV?	
Wo haben Sie die HPV Impfung bekommmen?		Dove ha ricevuto 0 il vaccino contro l`HPV?	
Asked in:	✓ HPV adolescent		

Q7

Where have you heard about HPV vaccination?		Comment avez-vous entendu parler du vaccin HPV?	
Wo haben Sie von der HPV Impfung gehört?		Dove ha sentito parlare della vaccinazione HPV?	
Asked in:	✓ HPV parent<br>✓ HPV adolescent		

Q8

Was the HPV vaccine recommended by your teen`s school?	1. yes<br>2. no<br>3. doesn't want to disclose<br>4. doesn't know<br>5. missing	Est-ce que l'école de votre enfant a recommandé le vaccin HPV?	1. oui<br>2. non<br>3. ne veut pas divulguer<br>4. ne sait pas<br>5. missing
Wurde die HPV Impfung in der Schule Ihres Kindes empfohlen?	1. ja<br>2. nein<br>3. möchte die Frage nicht beantworten<br>4. weiss nicht<br>5. keine Antwort	Il vaccino contro l'HPV per suo/a figlio/a le è stato raccomandato dalla scuola?	1. si<br>2. no<br>3. non vuole rivelare<br>4. non sa<br>5. mancante
Asked in:	✓ HPV parent		

Was the HPV vaccine recommended by your school?	1. yes<br>2. no<br>3. doesn't want to disclose<br>4. doesn't know<br>5. missing	Est-ce que votre école a recommandé le vaccin HPV?	1. oui<br>2. non<br>3. ne veut pas divulguer<br>4. ne sait pas<br>5. missing

Deml MJ, *et al. BMJ Open* 2019; 9:e032218. doi: 10.1136/bmjopen-2019-032218

Wurde die HPV Impfung in Ihrer Schule empfohlen?	1. ja<br>2. nein<br>3. möchte die Frage nicht beantworten<br>4. weiss nicht<br>5. keine Antwort	Il vaccino contro l'HPV le è stato raccomandato dalla scuola?	1. si<br>2. no<br>3. non vuole rivelare<br>4. non sa<br>5. mancante

Asked in:	✓ HPV adolescent

Q9

How do you feel about offering the HPV vaccine at school?	1. Very supportive<br>2. Somewhat supportive<br>3. Not sure<br>4. Somewhat unsupportive<br>5. Very unsupportive<br>6. doesn't want to disclose<br>7. doesn't know<br>8. missing	Quel est votre niveau de soutien pour l'offert de la vaccination HPV à l'école?	1. Très favorable<br>2. Un peu favorable<br>3. Pas sûr.e<br>4. Un peu défavorable<br>5. Très défavorable<br>6. ne veut pas divulguer<br>7. ne sait pas<br>8. missing
Wie finden sie es, dass die HPV Impfung über die Schule angeboten wird ?	1. sehr unterstützens wert<br>2. unterstützens wert<br>3. nicht sicher<br>4. nicht sehr unterstützens wert<br>5. nicht unterstützens wert<br>6. möchte die Frage nicht beantworten<br>7. weiss nicht<br>8. keine Antwort	Cosa ne pensa del fatto che il vaccino contro l'HPV venga proposto nelle scuole?	1. fortemente d'accordo<br>2. d'accordo<br>3. non sicuro/a<br>4. in disaccordo<br>5. fortemente in disaccordo<br>6. non vuole rivelare<br>7. non vuole rivelare<br>8. mancante

Asked in:	✓ HPV parent<br>✓ HPV adolescent

Q10

Who did you consult with when deciding		Qui est-ce que vous avez consulté en	

Deml MJ, *et al. BMJ Open* 2019; 9:e032218. doi: 10.1136/bmjopen-2019-032218

whether or not to get the HPV vaccine for your teen?		décidant si vous alliez vacciner Votre enfant a-t-il.elle avec le vaccin HPV?	
Wen haben Sie konsultiert um zu entscheiden, ob Sie für Ihr Kind eine HPV Impfung machen lassen sollten?		Con chi si è consultato/a quando si è trattato di decidere se somministrare il vaccino contro l`HPV a suo/a figlio/a?	

Asked in:	✓ HPV parent

Who did you consult with when deciding whether or not to get the HPV vaccine?		Qui est-ce que vous avez consulté en décidant si vous alliez vous vacciner avec le vaccin HPV?	
Wen haben Sie konsultiert um zu entscheiden, ob Sie für haben Sie eine HPV Impfung machen lassen sollten?		Con chi si è consultato/a quando si è trattato di decidere se ricevere il vaccino contro l`HPV?	

Asked in:	✓ HPV adolescent

Theme 4: Parent-Provider Relationship

Q1

Do you agree or disagree with the following statements:	Veuillez indiquer à quel point vous êtes d'accord ou en désaccord avec les énoncés suivants:
Stimmen Sie den folgenden Aussagen zu?	È d'accordo o in disaccordo con le seguenti affermazioni:

Asked in:	✓ Childhood vaccinations

Deml MJ, *et al. BMJ Open* 2019; 9:e032218. doi: 10.1136/bmjopen-2019-032218

Q2

It is my responsibility as a parent to actively research health decisions for my child.<br><br>READ OUT ANSWER OPTIONS	1. strongly agree<br>2. agree<br>3. neither agree nor disagree<br>4. disagree<br>5. Strongly disagree<br>6. doesn't want to disclose<br>7. doesn't know<br>8. missing	En tant que parent, c'est ma responsabilité de me renseigner sur les décisions de santé pour mon enfant de manière active.<br><br>LIRE TOUTES LES REPONSES POSSIBLES	1. Tout à fait d'accord<br>2. D'accord<br>3. Ni en accord, ni en désaccord<br>4. pas d'accord<br>5. Pas du tout d'accord<br>6. ne veut pas divulguer<br>7. ne sait pas<br>8. missing
Es ist meine Verantwortung als Elternteil mich aktiv über gesundheitliche Entscheidungen für mein Kind zu informieren.<br><br>ANTWORTMÖGLICHKEITEN VORLESEN	1. stimme stark zu<br>2. stimme zu<br>3. weder noch<br>4. stimme nicht zu<br>5. stimme gar nicht zu<br>6. möchte die Frage nicht beantworten<br>7. weiss nicht<br>8. keine Antwort	È mia responsabilità di genitore informarmi attivamente sulle decisioni che riguardano la salute di mio/a figlio/a.<br><br>LEGGERE LE RISPOSTE POSSIBILI	1. Fortemente d'accordo<br>2. d'accordo<br>3. né d'accordo né in disaccordo<br>4. in disaccordo<br>5. Fortemente in disaccordo<br>6. non vuole rivelare<br>7. non sa<br>8. mancante
Asked in:	✓ Childhood vaccinations		

Q3

I took an active role in choosing my child's doctor.<br><br>READ OUT ANSWER OPTIONS	1. strongly agree<br>2. agree<br>3. neither agree nor disagree<br>4. disagree<br>5. Strongly disagree<br>6. doesn't want to disclose<br>7. doesn't know<br>8. missing	J'ai joué un rôle actif en choisissant le médecin de mon enfant.<br><br>LIRE TOUTES LES REPONSES POSSIBLES	1. Tout à fait d'accord<br>2. D'accord<br>3. Ni en accord, ni en désaccord<br>4. pas d'accord<br>5. Pas du tout d'accord<br>6. ne veut pas divulguer<br>7. ne sait pas<br>8. missing

Ich habe eine aktive Rolle dabei gespielt, den Arzt/die Ärztin für mein Kind auszusuchen.<br><br>ANTWORTMÖGLICHKEITEN VORLESEN	1. stimme stark zu<br>2. stimme zu<br>3. weder noch<br>4. stimme nicht zu<br>5. stimme gar nicht zu<br>6. möchte die Frage nicht beantworten<br>7. weiss nicht<br>8. keine Antwort	Ho assunto un ruolo attivo quando si è trattato di scegliere il medico di mio/a figlio/a<br><br>LEGGERE LE RISPOSTE POSSIBILI	1. Fortemente d'accordo<br>2. d'accordo<br>3. né d'accordo né in disaccordo<br>4. in disaccordo<br>5. Fortemente in disaccordo<br>6. non vuole rivelare<br>7. non sa<br>8. mancante
Asked in:		✓ Childhood vaccinations	

Q4

I chose a doctor for my child who shares my views on health.<br><br>READ OUT ANSWER OPTIONS	1. strongly agree<br>2. agree<br>3. neither agree nor disagree<br>4. disagree<br>5. Strongly disagree<br>6. doesn't want to disclose<br>7. doesn't know<br>8. missing	J'ai choisi un médecin qui partage mes perspectives sur la santé.<br><br>LIRE TOUTES LES REPONSES POSSIBLES	1. Tout à fait d'accord<br>2. D'accord<br>3. Ni en accord, ni en désaccord<br>4. pas d'accord<br>5. Pas du tout d'accord<br>6. ne veut pas divulguer<br>7. ne sait pas<br>8. missing
Ich habe einen Arzt/ eine Ärztin ausgesucht, die meine Ansichten zum Thema Gesundheit teilt.<br><br>ANTWORTMÖGLICHKEITEN VORLESEN	1. stimme stark zu<br>2. stimme zu<br>3. weder noch<br>4. stimme nicht zu<br>5. stimme gar nicht zu<br>6. möchte die Frage nicht beantworten<br>7. weiss nicht<br>8. keine Antwort	Ho scelto un medico per mio/a figlio/a che condivide le mie stesse opinioni sulla salute<br><br>LEGGERE LE RISPOSTE POSSIBILI	1. Fortemente d'accordo<br>2. d'accordo<br>3. né d'accordo né in disaccordo<br>4. in disaccordo<br>5. Fortemente in disaccordo<br>6. non vuole rivelare<br>7. non sa<br>8. mancante
Asked in:		✓ Childhood vaccinations	

Q5

If I disagree or am uncertain about the advice of a nurse or a doctor, I am comfortable saying so.<br><br>READ OUT ANSWER OPTIONS	1. strongly agree<br>2. agree<br>3. neither agree nor disagree<br>4. disagree<br>5. Strongly disagree<br>6. doesn't want to disclose<br>7. doesn't know<br>8. missing	Si je ne suis pas d'accord ou ne suis pas sûr.e concernant les conseils d'une infirmière ou d'un médecin, je suis suffisamment à l'aise pour le dire.<br><br>LIRE TOUTES LES REPONSES POSSIBLES	1. Tout à fait d'accord<br>2. D'accord<br>3. Ni en accord, ni en désaccord<br>4. pas d'accord<br>5. Pas du tout d'accord<br>6. ne veut pas divulguer<br>7. ne sait pas<br>8. missing
Wenn ich mit dem Rat eines Arztes oder einer Krankenschwester nicht einverstanden bin, oder diesbezüglich unsicher bin, traue ich mich das zu sagen.<br><br>ANTWORTMÖGLICHKEITEN VORLESEN	1. stimme stark zu<br>2. stimme zu<br>3. weder noch<br>4. stimme nicht zu<br>5. stimme gar nicht zu<br>6. möchte die Frage nicht beantworten<br>7. weiss nicht<br>8. keine Antwort	Se non sono d'accordo o se ho dei dubbi riguardo ai consigli di un infermiere/a o di un medico, non mi pongo problemi a dirlo.<br><br>LEGGERE LE RISPOSTE POSSIBILI	1. Fortemente d'accordo<br>2. d'accordo<br>3. né d'accordo né in disaccordo<br>4. in disaccordo<br>5. Fortemente in disaccordo<br>6. non vuole rivelare<br>7. non sa<br>8. mancante
Asked in:		✓ Childhood vaccinations	

Q6

How many of the recommended well-child visits from birth until now has your child completed?<br><br>READ OUT ANSWER OPTIONS	1. All recommended visits<br>2. Some but maybe not all recommended visits<br>3. None of them<br>4. doesn't want to disclose<br>5. doesn't know<br>6. missing	Dès sa naissance et jusqu'à présent, à combien de contrôles de santé recommandés routines votre enfant est-il allé?	1. Tous les contrôles recommandés<br>2. Quelques-uns mais pas tous les contrôles recommandés<br>3. Aucun des contrôles

Deml MJ, *et al. BMJ Open* 2019; 9:e032218. doi: 10.1136/bmjopen-2019-032218

		LIRE TOUTES LES REPONSES POSSIBLES	recommandés<br>4. Ne veut pas divulguer<br>5. ne sait pas<br>6. missing
Wie viele der empfohlenen Vorsorgeuntersuchungen hat Ihr Kind von Geburt an bis jetzt erhalten?<br><br>ANTWORTMÖGLICHKEITEN VORLESEN	1. alle empfohlenen Vorsorgeuntersuchungen<br>2. Einige aber vielleicht nicht alle empfohlenen Vorsorgeuntersuchungen<br>3. Keine der empfohlenen Vorsorgeuntersuchungen<br>4. möchte die Frage nicht beantworten<br>5. weiss nicht<br>6. keine Antwort	Quante delle visite mediche periodiche raccomandate per suo/a figlio/a ha effettuato dalla nascita fino ad oggi?<br><br>LEGGERE LE RISPOSTE POSSIBILI	1. Tutte le visite raccomandate<br>2. Alcune ma forse non tutte le visite raccomandate<br>3. Nessuna di queste<br>4. non vuole rivelare<br>5. non sa<br>6. mancante

Asked in:	✓ Childhood vaccinations

Q7

Did you take your child to the doctor for a child development checkup between the ages of 11 and 14?	1. Yes<br>2. No<br>3. doesn't want to disclose<br>4. doesn't know<br>5. missing	Avez-vous amené votre enfant chez le médecin pour un contrôle de santé routine entre ses 11 et 14 ans?<br><br>IL S'AGIT ICI D'UN CONTROLE NORMAL ET NON PAS UNE VISITE D'URGENCE OU POUR UNE MALADIE	1. Oui<br>2. Non<br>3. ne veut pas divulguer<br>4. ne sait pas<br>5. missing

Deml MJ, *et al. BMJ Open* 2019; 9:e032218. doi: 10.1136/bmjopen-2019-032218

Haben Sie Ihr Kind im Alter zwischen 11 und 14 für eine Vorsorgeuntersuchung zum Arzt gebracht?	1. ja<br>2. nein<br>3. möchte die Frage nicht beantworten<br>4. weiss nicht<br>5. keine Antwort	Ha portato suo/a figlio/a ad effettuare una visita di controllo medico tra gli 11 e i 14 anni?	1. Si<br>2. No<br>3. non vuole rivelare<br>4. non sa<br>5. mancante

Asked in:	✓ HPV parent

Q8

When [name of target child] was an infant (0-2 years old), which of the following childcare options did you use? Please indicate all that apply.<br><br>READ OUT ANSWER OPTIONS	1. I (or my partner) stayed home with him/her.<br>2. Other family cared for him/her.<br>3. A nanny cared for him/her in my home.<br>4. He/she attended a small, home-based day care.<br>5. He/she attended private day care.<br>6. He/she attended public day care.<br>7. other<br>8. doesn't want to disclose<br>9. doesn't know<br>10. missing	Quand [name of target child] était un bébé (entre 0 et 2 ans), auxquels des services de garde d'enfance suivants avez-vous eu recours?<br><br>LIRE TOUTES LES REPONSES POSSIBLES	1. Soit moi, soit mon/ma partenaire l'a gardé à la maison.<br>2. Un autre membre de la famille l'a gardé.<br>3. Une nounou ou une nourrice l'a gardé chez nous.<br>4. Il/elle allait chez une maman de jour<br>5. Il/elle allait en garderie privée.<br>6. Il/elle allait en garderie publique<br>7. Autre<br>8. ne veut pas divulguer<br>9. ne sait pas<br>10. missing
Als [name of target child] ein Kleinkind war (0-2 Jahre alt), wurde es regelmässig von jemand anderem betreut ?	1. Ich blieb zuhause /mein(e) Partner(in) blieb zuhause<br>2. Jemand anderes aus der Familie schaute zu ihr/ihm	Quando [name of target child] era un neonato (da 0 a due anni d'età), quali delle seguenti opzioni di cura infantile ha utilizzato?	1. Io (o il mio partner) siamo stati a casa con lui/lei<br>2. Altra famiglia si è occupata di lui/lei<br>3. Una tata si è occupata di

Deml MJ, *et al. BMJ Open* 2019; 9:e032218. doi: 10.1136/bmjopen-2019-032218

ANTWORTMÖGLICHKEITEN VORLESEN	3. Eine Nanny schaute zu ihm/ihr<br>4. Er/sie besuchte eine Tagesmutter<br>5. Er/sie war in der privaten Kinderkrippe/Spielgruppe<br>6. Er/sie war in der öffentlichen Kinderkrippe/Spielgruppe<br>7. anderes<br>8. möchte die Frage nicht beantworten<br>9. weiss nicht<br>10. keine Antwort	LEGGERE LE RISPOSTE POSSIBILI	lui/lei a casa mia<br>4. Ha frequentato una famiglia diurna<br>5. Lui/lei ha frequentato l'asilo nido privato<br>6. Lui/lei ha frequentato l'asilo nido pubblico<br>7. altro<br>8. non vuole rivelare<br>9. non sa<br>10. mancante
Asked in:	✓ Childhood vaccinations		

If Q8 = 4, 5 or 6, ask Q9

Q9

And how old was he/she when he/she started day care?		Et quel âge avait-il/elle quand il/elle est entré.e en garderie? [i.e. entré.e en collectivité en dehors de la maison]	
Und wie alt war er/sie als er/sie in die Krippe/Spielgruppe kam?		Che età aveva quando ha incominciato l'asilo?	
Asked in:	✓ Childhood vaccinations		

Theme 6: Provider questions

Q1

For the purposes of our study, we are interested in providers who practice conventional medicine, also referred to as allopathic or biomedical medicine, and providers who practice complementary and/or alternative medicine.		Pour cette étude, nous nous intéressons aux médecins et aux praticiens qui pratiquent la médecine conventionnelle, également appelée la médecine allopathique ou biomédicale, et aux médecins et praticiens qui pratiquent la médecine complémentaire et/ou alternative.	
Im Rahmen dieser Studie sind wir sowohl an Ärztinnen und Ärzten interessiert, die Schulmedizin praktizieren, wie auch an Praktikern und Ärztinnen und Ärzten, die Komplementär- oder Alternativmedizin ausüben.		Per gli obbiettivi del nostro studio, siamo interessati ai professionisti che praticano medicina convenzionale, anche riferita alla medicina allopatica e biomedicina, e professionisti che praticano medicina complementare e/o alternativa.	

Asked in:	✓ Provider

Q2

In order to obtain more background about you and your practices, could you please tell me if you are a licensed medical doctor in Switzerland?	1. Yes<br>2. No<br>3. missing	Pour que nous puissions avoir plus d'informations sur vous et vos pratiques, pourriez-vous m'indiquer si vous êtes un médecin autorisé à pratiquer la	1. Oui<br>2. Non<br>3. pas de réponse

Deml MJ, *et al. BMJ Open* 2019; 9:e032218. doi: 10.1136/bmjopen-2019-032218

		médecine en Suisse?	
Um etwas mehr über Sie und Ihre Praxis zu erfahren, möchten wir wissen, ob Sie ein in der Schweiz anerkannter Arzt/eine anerkannte Ärztin sind?	1. Ja<br>2. Nein<br>3. keine Antwort	Potrebbe per cortesia dirmi se è un medico con licenza Svizzera?	1. Sì<br>2. No<br>3. mancante

Asked in:	✓ Provider

Q3

Have you undertaken any additional specialist training in any discipline of complementary and/or alternative medicine?	1. Yes<br>2. No<br>3. missing	Avez-vous effectué une formation supplémentaire pour obtenir une spécialisation dans une discipline de la médecine complémentaire et/ou alternative ?	1. Oui<br>2. Non<br>3. pas de réponse
Haben Sie zusätzliche spezielle Weiterbildungen in einem komplementärmedizinischen oder alternativen Bereich gemacht?	1. Ja<br>2. Nein<br>3. keine Antwort	Ha intrapreso una formazione supplementare in una qualsiasi disciplina di medicina complementare e/o alternativa?	1. Sì<br>2. No<br>3. mancante

Asked in:	✓ Provider

If Q3 = 1, ask Q4

Q4

Which ones?	1. Anthroposophic medicine	Lesquelles?	1. la médecine anthroposophique

Deml MJ, *et al. BMJ Open* 2019; 9:e032218. doi: 10.1136/bmjopen-2019-032218

READ ITEM BY ITEM, CHECK IF YES FOR EACH.	2. Traditional Chinese Medicine / Acupuncture<br>3. Homeopathic medicine<br>4. Phytotherapy (i.e. plant-based/herbal remedies)<br>5. other(s)<br>6. no answer	LIRE CHAQUE REPONSE INDIVIDUELLE, COCHER SI OUI	2. l'acupuncture et pharmacothérapie chinoise<br>3. l'homéopathie<br>4. la phytothérapie<br>5. d'autres(s)<br>6. pas de réponse
Welche?<br><br>ANTWORTMÖGLICHKEITEN VORLESEN, ANKREUZEN FALLS JA	1. Anthroposophische Medizin<br>2. Traditionelle Chinesische Medizin / Akupunktur<br>3. Homöopathie<br>4. Phytotherapie (pflanzliche Arzneien)<br>5. Anderes:<br>6. Keine Antwort	Quali?<br><br>LEGGERE LE RISPOSTE POSSIBILI	1. Medicina antroposofica<br>2. Medicina tradizionale cinese / agopuntura<br>3. Medicina omeopatica<br>4. Fitoterapia (ovvero medicina a base di piante / erbe)<br>5. Altro/i<br>6. mancante

Asked in:	✓ Provider

If Q4 = 5, ask Q5

Q5

Please specify:		Précisez s.v.p	
Bitte präzisieren:		Per favore specifichi:	

Asked in:	✓ Provider

If Q2=2, ask Q6

Q6

Ok. Thank you. Do you provide any complementary	1. Yes<br>2. No	D'accord. Merci pour vos	1. Oui<br>2. Non

Deml MJ, *et al. BMJ Open* 2019; 9:e032218. doi: 10.1136/bmjopen-2019-032218

or alternative medicines to your patients?.	3. missing	réponses. Est-ce que vous employez de la médecine complémentaire et/ou alternative avec vos patients ?	3. pas de réponse
Vielen Dank. Bieten Sie ihren Patienten/Patientinnen irgendwelche komplementärmedizinischen oder alternativen Therapien an?	1. Ja<br>2. Nein<br>3. keine Antwort	Ok. Thank you. Do you provide any complementary or alternative medicines to your patients?	1. Sì<br>2. No<br>3. mancante

Asked in:	✓ Provider

If Q6 = 1, ask Q7

Q7

Which ones?<br><br>READ ITEM BY ITEM, CHECK IF YES FOR EACH.	1. Anthroposophic medicine<br>2. Traditional Chinese Medicine / Acupuncture<br>3. Homeopathic medicine<br>4. Phytotherapy (i.e. plant-based/herbal remedies)<br>5. other(s)<br>6. no answer	Lesquelles?<br><br>LIRE CHAQUE REPONSE INDIVIDUELLE, COCHER SI OUI	1. la médecine anthroposophique<br>2. l'acupuncture et pharmacothérapie chinoise<br>3. l'homéopathie<br>4. la phytothérapie<br>5. d'autres(s)<br>6. pas de réponse
Welche?<br><br>ANTWORTMÖGLICHKEITEN VORLESEN, ANKREUZEN FALLS JA	1. Anthroposophische Medizin<br>2. Traditionelle Chinesische Medizin / Akupunktur<br>3. Homöopathie	Quali?<br><br>LEGGERE LE RISPOSTE POSSIBILI	1. Medicina antroposofica<br>2. Medicina tradizionale cinese / agopuntura<br>3. Medicina omeopatica

Deml MJ, *et al. BMJ Open* 2019; 9:e032218. doi: 10.1136/bmjopen-2019-032218

	4. Phytotherapie (pflanzliche Arzneien)<br>5. Anderes:<br>6. Keine Antwort		4. Fitoterapia (ovvero medicina a base di piante / erbe)<br>5. Altro/i<br>6. mancante
Asked in:	✓ Provider		

If Q7 = 5, ask Q8

Q8

Please specify:		Précisez s.v.p	
Bitte präzisieren:		Per favore specifichi:	
Asked in:	✓ Provider		

If Q1 = 2, ask Q9

Q9

What type of complementary and/or alternative therapies do you provide to your patients?<br><br>READ ITEM BY ITEM, CHECK IF YES FOR EACH.	1. Anthroposophic medicine<br>2. Traditional Chinese Medicine / Acupuncture<br>3. Homeopathic medicine<br>4. Phytotherapy (i.e. plant-based/herbal remedies)<br>5. other(s)<br>6. no answer	Quel type de thérapies complémentaires et/ou alternatives fournissez-vous à vos patients ?<br><br>LIRE CHAQUE REPONSE INDIVIDUELLE, COCHER SI OUI	1. la médecine anthroposophique<br>2. l'acupuncture et pharmacothérapie chinoise<br>3. l'homéopathie<br>4. la phytothérapie<br>5. d'autres(s)<br>6. pas de réponse
Welche komplementären und/oder alternativen Therapien bieten Sie ihren Patienten und Patientinnen an?	1. Anthroposophische Medizin<br>2. Traditionelle Chinesische Medizin / Akupunktur<br>3. Homöopathie	Che tipo di terapie complementari e/o alternative	1. Medicina antroposofica<br>2. Medicina tradizionale cinese / agopuntura

Deml MJ, *et al. BMJ Open* 2019; 9:e032218. doi: 10.1136/bmjopen-2019-032218

ANTWORTMÖGLICHKE ITEN VORLESEN, ANKREUZEN FALLS JA	4. Phytotherapie (pflanzliche Arzneien)<br>5. Anderes:<br>6. Keine Antwort	offre ai suoi pazienti?<br><br>LEGGERE LE RISPOSTE POSSIBILI	3. Medicina omeopatica<br>4. Fitoterapia (ovvero medicina a base di piante / erbe)<br>5. Altro/i<br>6. mancante

Asked in:	✓ Provider

If Q9 = 5, ask Q10

Q10

Please specify:		Précisez s.v.p	
Bitte präzisieren:		Per favore specifichi:	

Asked in:	✓ Provider

Deml MJ, *et al. BMJ Open* 2019; 9:e032218. doi: 10.1136/bmjopen-2019-032218
