# Supplementary material for: Determinants of vaccine hesitancy in Switzerland: study protocol of a mixed-methods national research programme
Source: BMJ Open. 2019 Nov 2;9(11):e032218. doi: 10.1136/bmjopen-2019-032218 (PMC6830664; doi:10.1136/bmjopen-2019-032218)
Supplement: Author's manuscript [file bmjopen-2019-032218.draft_revisions.pdf]

BMJ Open

BMJ Open is committed to open peer review. As part of this commitment we make the peer review history of every article we publish publicly available.

When an article is published we post the peer reviewers' comments and the authors' responses online. We also post the versions of the paper that were used during peer review. These are the versions that the peer review comments apply to.

The versions of the paper that follow are the versions that were submitted during the peer review process. They are not the versions of record or the final published versions. They should not be cited or distributed as the published version of this manuscript.

BMJ Open is an open access journal and the full, final, typeset and author-corrected version of record of the manuscript is available on our site with no access controls, subscription charges or pay-per-view fees (http://bmjopen.bmj.com).

If you have any questions on BMJ Open's open peer review process please email info.bmjopen@bmj.com

BMJ Open

Determinants of Vaccine Hesitancy in Switzerland - Study Protocol of a Mixed Methods National Research Program

Journal:	*BMJ Open*
Manuscript ID	bmjopen-2019-032218
Article Type:	Protocol
Date Submitted by the Author:	11-Jun-2019

Complete List of Authors:	Deml, Michael; Swiss TPH, Dept. Epidemiology & Public Health (EPH); University of Basel<br>Jafflin, Kristen; Swiss Tropical and Public Health Institute; University of Basel<br>Merten, Sonja; Swiss Tropical and Public Health Institute; University of Basel<br>Huber, Benedikt; HFR Fribourg Cantonal Hospital, Department of Pediatrics<br>Buhl, Andrea; Swiss Tropical and Public Health Institute; University of Basel<br>Frau, Eleonora ; University of Basel; University Department of Medicine, Kantonsspital Baselland, University of Basel<br>Mettraux, Valérie; University of Basel; University Department of Medicine, Kantonsspital Baselland, University of Basel<br>Sonderegger, Joanna; University of Basel; University Department of Medicine, Kantonsspital Baselland, University of Basel<br>Kliem, Paulina; University of Basel; University Department of Medicine, Kantonsspital Baselland, University of Basel<br>Cattalani, Rachele; University of Basel; University Department of Medicine, Kantonsspital Baselland, University of Basel<br>Krüerke, Daniel; Klinik Arlesheim AG<br>Pfeiffer, Constanze; Swiss Tropical and Public Health Institute; University of Basel<br>Burton-Jeangros, Claudine; Institute of Sociological Research, University of Geneva<br>Tarr, Philip ; University of Basel; University Department of Medicine, Kantonsspital Baselland, University of Basel

Keywords:	Vaccine hesitancy, Under-immunization, Childhood vaccinations, HPV vaccination, Complementary and alternative medicine (CAM), Patient-provider communication

SCHOLARONE™
Manuscripts

Title: Determinants of Vaccine Hesitancy in Switzerland - Study Protocol of a Mixed Methods National Research Program

Authors:
Michael Deml [a,b*]
michael.deml@swisstph.ch
Kristen Jafflin [a,b*]
kristen.jafflin@swisstph.ch
Sonja Merten [a,b*]
sonja.merten@swisstph.ch
Benedikt Huber [d]
Benedikt.Huber@h-fr.ch
Andrea Buhl [a,b]
andrea.buhl@unibas.ch
Eleonora Frau [b,c]
eleonora.frau@stud.unibas.ch
Valérie Mettraux [b,c]
valerie.mettraux@stud.unibas.ch
Joanna Sonderegger [b,c]
joanna.sonderegger@stud.unibas.ch
Paulina Kliem [b,c]
paulina.kliem@stud.unibas.ch
Rachele Cattalani [b,c]
rachele.cattalani@stud.unibas.ch
Daniel Krüerke [e]
daniel.krueerke@klinik-arlesheim.ch
Constanze Pfeiffer [a,b]
constanze.pfeiffer@unibas.ch
Claudine Burton-Jeangros [f]
claudine.jeangros@unige.ch
Philip E. Tarr [b,c]
philip.tarr@unibas.ch

[a]Swiss Tropical and Public Health Institute (Swiss TPH); Socinstrasse 57; 4051 Basel, Switzerland
[b]University of Basel; Petersplatz 1; 4051 Basel, Switzerland
[c]University Department of Medicine, Kantonsspital Baselland, University of Basel; 4101 Bruderholz, Switzerland
[d]Department of Pediatrics, HFR Fribourg – Hôpital cantonal; Chemin des Pensionnats 2-6; 1708 Fribourg, Switzerland
[e]Resarch Department, Klinik Arlesheim; Pfeffingerweg 1, 4144 Arlesheim, Switzerland
[f]Institute of Sociological Research, University of Geneva; Boulevard du Pont-d'Arve 40; 1204 Geneva, Switzerland

** these authors contributed equally to the manuscript*

Corresponding author: Prof. Dr. med. Philip Tarr, University Department of Medicine, Kantonsspital Baselland, University of Basel; CH-4101 Bruderholz, Switzerland, Philip.tarr@unibas.ch, Phone +41 61 436 2212

For peer review only - http://bmjopen.bmj.com/site/about/guidelines.xhtml

Abstract

Introduction: Vaccine hesitancy is a complex public health issue referring to concerns about the safety, efficacy or need for vaccination. Relatively little is known about vaccine hesitancy in Switzerland. This ongoing study (2017-2021) focuses on biomedical and complementary and alternative medicine (CAM) providers and their patients since healthcare professionals play important roles in vaccination decision-making. This national research program seeks to assess the socio-cultural determinants of vaccine hesitancy regarding childhood and human papilloma virus (HPV) vaccines in Switzerland. We aim to provide a detailed characterization of vaccine hesitancy, including CAM and biomedical perspectives, patient-provider interactions, and sociocultural factors, to establish mediating effects of vaccine hesitancy on under-immunization, and to design an intervention to improve vaccination communication and counseling among physicians, parents, and adolescents.

Methods and analysis: Our transdisciplinary team employs a sequential exploratory mixed-methods study design. We have established a network of more than 120 medical providers across Switzerland, including more than 40 CAM practitioners. For the qualitative component, we conduct interviews with parents, youth, biomedical and CAM providers and observations of vaccination consultations and school vaccination information sessions. For the quantitative component, a sample of 1,350 parents of young children and 722 young adults (15-26 years) and their medical providers respond to questionnaires. We measure vaccine hesitancy with the Parent Attitudes about Child Vaccine 15-item survey and review vaccination certificates to assess vaccination status. We administer additional questions based on findings from qualitative research, addressing communication with medical providers, vaccine

For peer review only - http://bmjopen.bmj.com/site/about/guidelines.xhtml

information sources, and perceptions of risk control *vis-à-vis* vaccine preventable diseases. The questionnaires capture sociodemographics, political views, religion and spirituality, and moral foundations.

Ethics and dissemination: The study was approved by the local ethics committee. The results will be published in peer-reviewed journals and disseminated to healthcare professionals, researchers, and the public via conferences and public presentations.

Article Summary

Strengths and limitations of this study:

- A novelty of this research includes its large sample of CAM and biomedical providers, with consideration given to their vaccination perspectives and interactions with patients.
- There is a high demand for CAM in Switzerland, and previous studies have shown an association between CAM use and vaccine hesitancy.
- The mixed methods approach and transdisciplinary nature of this study provide further insights into the relationships between CAM and biomedical providers and vaccine hesitancy and uptake.
- Although the methodological choice to recruit patients via medical providers is advantageous because it allows for the examination of effects of provider characteristics on patient vaccination beliefs and behaviors, it limits our results because populations that do not regularly see medical providers are under-represented in our data.

Key words

- Vaccine hesitancy
- Under-immunization
- Childhood vaccinations
- HPV vaccination

For peer review only - http://bmjopen.bmj.com/site/about/guidelines.xhtml

- Complementary and alternative medicine (CAM)
- Mixed methods research
- Patient-provider communication
- Vaccination information sources

Introduction

Vaccines are ranked among the greatest public health achievements(1). Extensive evidence documents the efficacy, minimal side-effects, and cost-effectiveness of vaccines recommended by numerous national immunization programs, including that of the Swiss Federal Office of Public Health (FOPH)(2, 3). Despite public health successes of vaccines, a large number of people in Western countries express concerns about vaccine safety, efficacy or need, a behavior now referred to as *vaccine hesitancy*(4-6).

Vaccine hesitancy has gained increasing attention from the global public health community. In August 2015, the World Health Organization (WHO) referred to vaccine hesitancy as a "growing challenge for immunization programs" (7-9), responding to a 2014 report(10) from the WHO Strategic Advisory Group of Experts (SAGE) on immunization. In early 2019, WHO listed vaccine hesitancy among the top 10 threats to global health, citing a global need to address rising rates of measles and to eliminate cervical cancer by ramping up efforts to increase coverage with the human papillomavirus (HPV) vaccine(11).

While the importance of vaccine hesitancy is widely acknowledged, its determinants are incompletely understood and merit further investigation(4). Researchers having conducted a systematic review on determinants of vaccine hesitancy from a global perspective found that there was "no universal algorithm" (p. 2155) and recommended context-specific research(4). Other researchers have argued in favor of cross-disciplinary research, stating, "Vaccine hesitancy and

For peer review only - http://bmjopen.bmj.com/site/about/guidelines.xhtml

acceptance are complex, multi-faceted issues. Our understanding of them must be complex and multifaceted as well” (p. 279)(12).

When vaccine hesitancy leads to *under-immunization,* which we define as delaying or omitting some or all officially recommended vaccines, this provides an example of the underuse of a medical intervention considered to be safe and effective. Under-immunization is a public health concern because it can reduce population protection, also referred to as *herd immunity*, towards certain potentially dangerous infectious agents and increases the risk of disease outbreaks.

Studies suggest that personal, social(12, 13) and local cultural(6) networks are important determinants of vaccination decisions, in particular among those who under-vaccinate. Furthermore, people’s sources of information on health and vaccination-related information can play important roles in vaccination choices, with recent research pointing to the importance of ‘information overload,’ ‘misinformation,’ and higher levels of indecision among parents, particularly given the wide availability of (mis)information on the Internet(14-18).

Additionally, parent and provider perspectives that differ from official public health recommendations need to be considered in order to understand vaccine hesitancy(12). These may include intuitive rather than analytical cognitive styles, reluctance to consider the evidence suggesting vaccine safety/efficacy, and lowered willingness to trust information delivered by public health authorities, as shown in an adherence to complementary and alternative medicine (CAM)-oriented health values and preference of CAM over biomedicine(19). Studies also suggest that under-immunization and usage of CAM are determined by similar factors, such as alternative concepts of body, immunity, risk perception, anti-authoritarian orientation, and distrust of biomedical establishment and the pharmaceutical industry(6, 19).

For peer review only

Some of these associations may in part be due to confounding factors, such as higher income and education and distrust of medical systems(20). Other sociomedical trends may also play a role in heightened patient participation in decision-making, such as postmodern medicine(21), characterized by risk culture(22), healthism(23), and patients activating more agency in healthcare decisions(24). Finally, research findings suggest limitations to the efficacy of the evidence-based strategies that have generally employed by health authorities over the past decades to increase vaccination rates(19).

A key determinant of patient vaccination decision-making seems to be the attitudes, behavior, and knowledge of their medical provider(25-27). Important factors include the amount of time providers spend discussing vaccinations, their communication styles, and the information available to providers(28-31). As an example of medical providers' influence on vaccination decisions, one study in the US found that primary care providers with many parents who had vaccine exemptions for their children were more likely to have concerns about vaccine safety and were less likely to perceive individual and community benefits of vaccinations than primary care providers treating fewer children with vaccine exemptions(25). A qualitative study into vaccination decision-making in the US highlighted the importance of trust between healthcare professionals and parents (32). Finally, the way that providers broach vaccination during consultations can have a determinative role in vaccination uptake, with one study recommending further investigation of participatory (i.e. "What do you think about doing shots today?") vs. presumptive (i.e. "Today, we are going to do some shots.") approaches during patient-provider interactions(26).

For peer review only - http://bmjopen.bmj.com/site/about/guidelines.xhtml

The Swiss context

In Switzerland, childhood vaccinations are usually given by private practitioners, predominantly pediatricians and general internists. There is no national mandate for vaccination in non-epidemic settings in Switzerland, and no national vaccination registry. Despite this, vaccination coverage in Switzerland is high overall(33). The FOPH makes vaccination recommendations and distinguishes between basic[i] and complementary vaccinations(34). Both basic and complementary vaccines are reimbursed by basic mandatory health insurance when the official vaccination schedule is respected.

Given the absence of a national vaccination registry, the FOPH measures Swiss vaccination coverage at regular intervals(35). Vaccination programs and their implementation vary between the 26 cantons(34). For example, children from the French and Italian-speaking cantons have on average higher rates of measles vaccination coverage than in German-speaking cantons(36).

Cantonal coverage levels for HPV vaccination range from 79% (Valais) to 19% (Appenzell Innerhoden) for 2 doses of HPV vaccine. HPV coverage estimates only include 16-year-old girls, and estimates for boys are not yet available(37). The most common reasons for females not being vaccinated against HPV, according to a 2014 survey, included being too old, lack of information, fear of side effects, being against vaccination in general or against HPV vaccination only, HPV vaccination felt to be unnecessary, and simple logistical issues(38), with 7% and 6% of women not vaccinated against HPV reporting that the physician or friends/family recommended against HPV vaccine, respectively. Further research has shown that living in cantons with school-based vaccination increases uptake of HPV vaccination (39, 40). However, spatial variation modeling has shown that variables such as political

For peer review only

For peer review only - http://bmjopen.bmj.com/site/about/guidelines.xhtml

opinion, religion, and community opinion might be additional determinative factors in the presence of school-based vaccination programs(40). This suggests further research is needed to better understand vaccine hesitancy and school-based vaccination programs in Switzerland.

Vaccine hesitancy and under-immunization seem to be specific to certain population subgroups(34, 41, 42). For example, in Switzerland, measles cases, small epidemics(43), and under-immunization cluster around children attending anthroposophic (i.e. Rudolf Steiner, Waldorf) schools and around certain providers of CAM(41). One cross-sectional survey (N=1007) was administered for pediatric patients in an urban pediatric emergency department in German-speaking Switzerland. Researchers found that those who did not fully accept basic vaccinations were more frequently CAM-users than non-users, which researchers point to as reflecting parent wishes rather than physicians' recommendations(44). This relationship merits further investigation and discussion.

Studies in Switzerland show high rates of CAM use and favorable opinions, with 25% of Swiss survey respondents aged 15 and older of the 2007 Swiss Health Survey stating that they had used CAM in the previous year (45, 46). Other researchers assessed surveys conducted among the general population, doctors, hospitalized patients, and obstetric institutions and found that approximately 50% of the Swiss population had used CAM and that 85% of the population would like the cost of CAM to be covered by basic health insurance (47). CAM providers in Switzerland are often physicians trained in conventional medicine who have then obtained additional CAM training(48), and basic mandatory health insurance reimburses four CAM services when provided by licensed physicians: (1)

For peer review only

anthroposophic medicine, (2) Traditional Chinese Medicine (TCM) and acupuncture, (3) homeopathy, and (4) phytotherapy(49).

Rationale for the research project

The scientific literature points to existing research gaps, such as a lack of detailed information on vaccine hesitancy, patient-provider communication in Switzerland, vaccination information sources, and relations to the high prevalence of CAM use in Switzerland. This highlights the interest in an exploratory mixed method methods study investigating the vaccination perspectives and practices of patients and both CAM and biomedical providers. Our research focuses specifically on childhood vaccinations recommended in Switzerland (50) and on the vaccination against HPV, which is included in the Swiss FOPH recommendations for all adolescents 11-14 years of age. However, vaccination against HPV is recommended as a routine vaccination for females and as an complementary vaccination for males(50).

Objectives and aims

1) to explore vaccine hesitancy and provide a detailed characterization of its health system, patient-provider interaction, health communication, information sources, decision-making process, demographic, geographical and sociocultural correlates in Switzerland
2) to assess the socio-cultural and health system determinants of vaccine hesitancy and under-immunization with childhood and HPV vaccines in Switzerland. This objective additionally aims at establishing the mediating effect of vaccine hesitancy on under-immunization.

For peer review only - http://bmjopen.bmj.com/site/about/guidelines.xhtml

3) to use the knowledge gained as the necessary background to design and submit a pilot intervention and to design tailored interventions to address vaccine hesitancy in Switzerland

Methods and design

Our research takes place in the setting of an ongoing Swiss national research program (NRP 74). The study protocol underwent successful peer review and is entirely funded by the Swiss National Science Foundation for 4 years (2017-2021). For further information, refer to: http://www.nfp74.ch/en/projects/out-patient-care/project-tarr. It represents a transdisciplinary[ii] collaboration that views vaccine hesitancy as a complex, multi-faceted phenomenon. The study team is composed of researchers and medical practitioners with a varied range of backgrounds and training. An infectious disease specialist and internist leads the core study team, which includes sociologists, anthropologists, and public health specialists who collaborate closely with a steering committee composed of clinicians trained in biomedicine and CAM, a researcher in anthroposophic medicine, public health experts, and policy makers.

The project applies mixed methods through the use of a *sequential exploratory design* in order to study vaccine hesitancy across Switzerland, meaning that we first begin with qualitative methods in order to inform the tools utilized for the data collection through quantitative methods (52). We recruit participants from the 3 major language regions of Switzerland (German-, French-, and Italian-speaking) in order to examine vaccination decision-making throughout the country.

The study was approved by the local ethics committee (Ethikkommission Nordwest- und Zentralschweiz, EKNZ; project ID number 2017-00725), and ethics

For peer review only

approval covers all study participants, including adolescent and adult patients, as well as biomedical and CAM providers. All study participants receive information about the study and provide written informed consent.

For the study's qualitative component, we conduct semi-structured in-depth interviews with parents, youth, and biomedical physicians and CAM providers. For this purpose, a network of participating providers was established via the FOPH's Swiss Sentinella Surveillance Network and through direct recruitment by our research team, sharing recruitment documents (i.e. invitation letters and study flyers), making telephone calls, and employing snowball sampling. We also observe vaccination consultations with parents and providers and HPV information events in schools. For the study's quantitative component, we recruit participants from the practices of the network of CAM and biomedical providers across Switzerland and perform quantitative telephone interviews with parents of children 0 to 11 years of age and youth aged 15 to 26 years. We also interview the participating providers. The collection of information about vaccine hesitancy from providers and their patients allows for a comparison of their perceptions of vaccinations.

Some providers participate in both the qualitative and quantitative research components, while most participate only in the quantitative phase. Importantly, our team had already established trusting relationships with a number of CAM physicians by the time of grant submission. These contacts have been crucial in expanding our network of CAM providers in Switzerland for the quantitative study phase.

Qualitative methods

For peer review only - http://bmjopen.bmj.com/site/about/guidelines.xhtml

The qualitative methods of this research allow us to gain deeper insight into parent and youth vaccination decision-making and patient-provider interactions about vaccination in general practice settings. The qualitative methods additionally further our understanding about public health gaps in vaccination implementation programs in Switzerland by paying attention to how participants discuss the Swiss health system in relation to vaccination. Finally, the qualitative methods will inform the design of the quantitative study component tools.

The transdisciplinary research team drafted interview guides and medical consultation observation guides to be used as qualitative study tools. The interview guides were written based on relevant vaccine hesitancy literature and guide researchers through semi-structured interviews with participants through the use of open-ended questions. Semi-structured interviews involve researchers asking the same questions systematically to all participants but allow the option for researchers to probe further by asking additional questions about themes emerging during the interview. The medical consultation observation guides allow researchers to systematically focus upon items of interest highlighted in vaccine hesitancy literature on patient-provider interactions (28-31). Both the interview guides and the observation guides were piloted for coherence and clarity.

An important concept in qualitative research to determine sample sizes is the concept of saturation, which indicates that similar results are consistently collected during data collection and adequately address the research questions at hand. When saturation has been attained in qualitative research, researchers can reasonably expect similar results if the research process were to continue(53, 54). We expect to reach data saturation with the number of qualitative face-to-face interviews and

For peer review only

For peer review only - http://bmjopen.bmj.com/site/about/guidelines.xhtml

observations mentioned below, but the qualitative nature of this approach allows us flexibility in adjusting the amount of data to be collected.

Qualitative interviews with biomedical and CAM providers

We conduct in-depth semi-structured qualitative interviews with at least 15 CAM providers and at least 15 biomedical physicians. Interview questions are organized by theme, including: (1) contextual information about the providers (i.e. type of training, type of medicine practiced, years of clinical experience, and descriptions of the types of patients they treat), (2) questions about interactions with their patients and typical vaccination consultations (i.e. amount of time spent discussing vaccination, communication strategies, and perceptions about communication training), and (3) views and perspectives on medicine, immunity, and the body (i.e. information sources on vaccinations, perceptions about their advantages and disadvantages, "natural acquired immunity" vs. vaccine acquired immunity, public health benefits compared to individual choice considerations, and vaccination rates and public health approaches in Switzerland). The interviews are audio recorded and transcribed verbatim.

Ethnographic observations of vaccination consultations

We observe a total of 30 vaccination consultations (15 with CAM providers, 15 with biomedical providers) with 10 different providers (5 CAM, 5 biomedical). We ask providers to allow us to observe consultations during which they discuss vaccination with parents for the first time. Researchers take ethnographic field notes about the observation in field journals in order to document what happens during the interactions. They then fill out a medical consultation observation guide, which

For peer review only - http://bmjopen.bmj.com/site/about/guidelines.xhtml

prompts them to record information about patients and providers, the reason for the consultation, who initiated the vaccination discussion, if vaccinations were discussed in a participatory or presumptive manner, questions asked by patients, if and how patients expressed hesitancy towards vaccination, researchers' perceptions of the patients' ability to understand the vaccination discussion, the vaccination decision, the amount of time spent discussing vaccination, information source materials, and the researchers' perceptions of the mood, emotions, and communication between parents and providers. After observing the medical consultations, researchers use ethnographic field notes and observation guide as the basis to write observations into a descriptive, narrative format of each observed consultation.

Qualitative interviews with parents

We conduct in-depth qualitative interviews with 30 vaccine hesitant parents and 10 parents who vaccinate according to the FOPH vaccination schedule. We purposefully sample more vaccine hesitant parents, particularly those who consult CAM providers for their children, since an important study assumption is that vaccine hesitancy, under-immunization by parents and their usage of CAM are co-determined. We ask parents questions thematically about: (1) family composition and parental roles (i.e. work, childcare, child healthcare decisions); (2) children's health, healthcare, and lifestyle (i.e. types of healthcare professionals parents consult for their children, health beliefs and practices, CAM use, preventive practices, etc.); (3) immunization status of the children with the support of vaccination booklets during the interview; (4) vaccination perspectives (views on individualized vaccination calendars and views on the public health benefits or consequences of vaccination) and (5) vaccination experiences (the vaccination

For peer review only - http://bmjopen.bmj.com/site/about/guidelines.xhtml

decision-making process, vaccination discussions with healthcare professionals and others, and perceptions of social pressure to vaccinate or not vaccinate).

Qualitative research focus on HPV vaccination: Youth and parents

There is limited published data on adolescent female views on HPV vaccine(38, 55) and no data on adolescent male views on HPV vaccine in Switzerland. Therefore, we will additionally conduct qualitative in-depth interviews with approximately 40 youth (approximately 25 males and 15 females) recruited through the HPV quantitative research discussed below. We purposively sample more male youth since there is no literature on male perspectives on HPV in Switzerland, and HPV vaccination recommendations are more recent for males than females. If youth provide their consent, the parents of 15 youth will be interviewed separately and in family focus group discussions (FGD) together with the youth. Themes of the interviews and FGD include: (1) questions relating to family composition and parental roles; (2) youth health, healthcare, and lifestyle (i.e. types of healthcare, professionals young adults/adolescents consult, health beliefs and practices, CAM use, preventive practices, etc.); (3) HPV immunization status; (4) HPV perspectives; (5) HPV vaccination experiences and the vaccination decision-making process. This last theme focuses particularly on discussions with parents, friends, acquaintances, significant others, potential sexual partners, information sources, schools' roles in providing vaccination information, and how or if the youth ever felt any social pressure to vaccinate or not vaccinate against HPV.

Qualitative research focus on HPV vaccination: Schools and public health officials

For peer review only - http://bmjopen.bmj.com/site/about/guidelines.xhtml

We observe 10 school information activities about HPV vaccines in 3-4 cantons. We choose cantons based on convenience in terms of access and ethical clearance to conduct ethnographic observations for research purposes. Researchers attend school-based events during which the HPV vaccination is presented to youth. In classrooms, our researchers will explain to students and instructors that we are there to observe and take notes on the content of the discussions as part of a study that examines how this type of information is communicated in schools. No identifying information will be documented or included in any dissemination of research results. Observation notes will be documented in field journals and subsequently written into a descriptive narrative format.

We purposively sample an estimated 2-3 public health officials from the FOPH, 2-3 cantonal authorities, and approximately 5 school physicians for expert interviews (n=10-15) in order to gain further information about how recommendations are made, discussed, planned, and implemented from a public health perspective. Interviews focus on perceptions of the implementation of vaccination programs from public health authority perspectives and are informed by specific questions from our qualitative inquiries.

Vaccination information sources

In order to better understand how information sources affect parent, youth and provider decision-making processes, we document and analyze sources of information that they discuss during the qualitative and quantitative interviews and medical consultation observations using both thematic analysis(56) and critical discourse analysis(57-59). Since the Internet's role in vaccine-related decision

For peer review only - http://bmjopen.bmj.com/site/about/guidelines.xhtml

making is well documented in the literature (14, 15, 60-62), we are particularly interested in parents' and youth's use of and interactions with Internet sources.

Qualitative data analysis

The primary research questions guiding the conduct of the qualitative research are: 1) how do parents and youth make vaccination decisions?; 2) how do medical providers, both CAM and biomedical, consider what vaccination recommendations to make to their patients?; 3) how do medical providers discuss vaccination with parents and youth; and 4) how are vaccination implementation programs envisaged from a public health perspective?

Since we work with diverse sources of qualitative data, we use the Framework Method(63) and grounded theory, particularly *constructivist grounded theory* as outlined by Charmaz (64), as guiding principles for our analysis. This allows our research team to structure an analytical framework throughout qualitative data analysis with the use of sensitizing concepts and to code data into relevant research themes while still being open to including categories and themes that emerge through qualitative data collection and analysis. Sensitizing concepts are used in qualitative research in order to guide researchers to selectively focus on emerging themes and categories relevant to the research questions(65); for this research, sensitizing concepts include patient-provider interactions, provider and patient interaction with information sources, health beliefs and practices, and interactions with and perceptions of the Swiss health system. With the focus on patient-provider interactions in vaccination decision-making, the choice to research these questions by interviewing parents, youth and medical providers, and observing vaccination consultations will allow us to triangulate our qualitative data in order to account for

For peer review only

the "multiple realities" of medical consultations(66). Finally, in order to fully appreciate the complexity of how people interact with health information in order to make vaccination choices, we will conduct both thematic analysis(56) and critical discourse analysis(57-59) of any sources of information mentioned as being important or determinative during vaccination discussions. A first manuscript containing initial results of the qualitative research phase has been submitted for publication (67).

Quantitative methods

The quantitative phase of our study seeks to explore and quantify the determinants of vaccine hesitancy and under-immunization with childhood and HPV vaccines in Switzerland. We measure vaccine hesitancy using the 15-item PACV (Parent Attitudes about Childhood Vaccines) survey instrument, a validated measure of vaccine hesitancy and under-immunization(68). We measure under-immunization through examination of participants' vaccination records.

The Questionnaires

As childhood and HPV vaccination involve different populations and are generally viewed differently by patients, we will survey two separate populations: parents of young children and youth (and their parents, if possible). In addition, we survey providers to gain a better understanding of views on vaccination in our diverse network of participating providers and the relationship between provider and patient views on vaccination.

To fully explore vaccine hesitancy and under-immunization in these diverse populations, we use 4 separate questionnaires: (1) a childhood vaccination

For peer review only - http://bmjopen.bmj.com/site/about/guidelines.xhtml

questionnaire to be administered to parents of young children, (2) a HPV vaccination survey for youth, (3) an HPV vaccination survey for parents, and (4) a questionnaire for providers.

All questionnaires include versions of the PACV adapted to the specific population surveyed. Parent and youth surveys also include questions about basic socio-demographic factors, including household composition, work and educational background, income, migration status and language knowledge. We have also drawn on results from the qualitative phase of our study to develop further questions around several themes, including (1) communication about vaccination with medical providers, (2) information sources consulted in vaccination decision-making, and (3) perceptions about risk and control concerning possible exposure to vaccine-preventable diseases. The HPV vaccination surveys also include questions specific to this vaccine and its administration in Switzerland, including questions on knowledge of HPV vaccine, sources of HPV vaccination, and perceptions of school-based vaccination programs. In addition, we ask both parents and youth a series of questions about health, including questions about health status, CAM usage, and medical care usage. The provider questionnaire also asks for details about providers' practices, particularly with regards to their practice of CAM. Finally, all questionnaires include questions about religion, spirituality, politics and moral foundations, based on the finding in a recent US study(69) that moral foundations are associated with vaccine hesitancy.

All questionnaires were first drafted in English before being translated into French, German and Italian. Key variables, including the PACV and other questions related to vaccination were translated using the forward and backward method. Two independent translators first translated into the target language (German, French or

For peer review only

Italian) and then reconciled any disparities in their translations. Next, two different, independent translators who were blinded to the original questions translated it back into English. The new versions were then pre-tested in all languages to assess understandability and clarity.

Power analysis

Childhood vaccination survey. A conservative estimate is a proportion of 15% vaccine-hesitant parents in the practices of the participating biomedical and CAM providers combined (NB: 7% un-vaccinated and 12% children with delayed vaccination were recorded in the 2012 FOPH survey) (70). The sample size calculation aims at identifying the number of participants needed to detect a significant association of specific individual risk factors with vaccine hesitancy, which is here considered as a binary outcome. We further assume that factors potentially associated with vaccine hesitancy are prevalent in 20% of the population and that the odds ratio between vaccine hesitancy and a relevant risk factor is at least 2. In this case, to detect a statistically significant association with such a risk factor at the level of 0.05 with a power of 0.8, 675 participants are needed. Due to the recruitment in selected provider practices, a certain clustering of parent's characteristics is to be expected for parents recruited in the same practice, in contrast to a simple random sample. Therefore, a design effect of 2 is assumed, leading to a total sample of 1,350 parents to be interviewed. This would equally allow detecting a 10% lower proportion of vaccination among parents who are vaccine-hesitant compared to a vaccination rate of 90% in the remaining population.

HPV vaccination survey. To investigate vaccine hesitancy and associations with under-immunization in the case of HPV vaccination, a separate sample of

For peer review only - http://bmjopen.bmj.com/site/about/guidelines.xhtml

participants is required. Similar to the first case we also assume 15% vaccine hesitant participants, and we further anticipate a 30% lower rate of vaccination among vaccine hesitant persons than in the entire population. With a given prevalence of HPV vaccination of 53%, this would mean that among vaccine hesitant persons only 37% would be vaccinated. With power=0.8 and alpha=0.05 a sample of 593 participants would be needed. To account for clustering, assuming a design effect of 2, the final sample would include 1,186 participants. However, given the relative novelty of HPV vaccines and the fact that it has been recommended in boys/adolescents only for the past 3 years, it is likely that the vaccine hesitancy rate is lower (e.g. 30%). With power=0.8 and alpha=0.05 a sample of 361 participants would be needed. To account for clustering, assuming a design effect of 2, the final sample would include 722 participants.

Based on this analysis, our study populations will include 1,350 parents of children aged 0 to 11 for the childhood vaccination study and 722 young people aged 15 to 21 years (for young women) or 15 to 26 years (for young men) for the HPV vaccination study.

Recruitment and survey populations

We recruit our participants through the offices of the network of CAM and biomedical providers. As Figure 1 shows, our provider network includes more than 120 CAM and biomedical providers working in all three language-regions in Switzerland and in almost every canton. Each dot represents a practice participating in the study: green dots represent CAM practitioners and blue dots biomedical providers. To ensure confidentiality, we place dots in the correct canton but do not

For peer review only

547 place them at the exact provider location. Group practices are represented by a
548 single dot.

549 Providers are CAM or biomedical providers who treat children and/or youth
550 and regularly deal with questions regarding vaccinations and vaccine decisions.
551 They include pediatricians, general practitioners, gynecologists, CAM physicians,
552 and CAM providers without formal medical training working independently or in
553 group practices.

554 We interview parents and young people by telephone. Each telephone
555 interview will last approximately 30-35 minutes. Parents will be recruited from urban
556 and rural medical practices in French, German and Italian speaking areas of
557 Switzerland. In addition, we interview each participating provider in order to examine
558 the relationships between provider perspectives, patient perspectives, and vaccine
559 hesitancy. For the HPV portion of our study, we will interview one of the participating
560 youth's parents if the youth allow us to do so.

561 To be eligible to participate in the study, participants fulfill the following
562 criteria. For the childhood vaccination sub-study, parents are 18 years old or older
563 with a child aged 0 to 11. For the quantitative HPV portion of our study, young
564 women are between the ages of 15 and 21 (born between July 1, 1997 and July 1,
565 2003) and young men are between the ages of 15 and 26 (born between July 1,
566 1992 and July 1, 2003). These differences are due to the timing of HPV vaccine
567 recommendations for girls and boys. HPV vaccine reimbursement for girls 11-14
568 (target age) and 15-19 (catch-up age) started January 1, 2008. HPV vaccination
569 reimbursement for males began on July 1, 2016 in Switzerland. Given the newness
570 of this recommendation, we enroll youth males born between July 1, 1992 and July
571 1, 2003 in order to gather data on all males aged 15 to 26 who have been eligible for

For peer review only

the HPV vaccine through Swiss vaccine programs. All study participants (parents, youth, and providers) must speak one of the main Swiss national languages (French, German or Italian) or English.

We recruit all parents and youth through providers in order to be able to link the data from the parents with their provider's data for a comparison by type of provider. We use a quota sampling technique as CAM providers are likely to see fewer patients in a given time-frame. Our goal is to recruit a maximum of 20 parents of young children and a maximum of 15 youth per provider based on a rigorous sampling approach in a defined period of time. Recruitment targets for group practices are no more than three times the target for individual providers. Thus, targets in dual practices are 40 parents and 30 youth, and for practices with 3 or more providers, targets are 60 parents and 45 youth. For every practice, the total number of eligible patients in a specific time period will be established, which allows post-hoc weighting to the known total of eligible patients in this time-period.

Given the diversity of providers and differences in our study sub-populations, we employ a variety of recruitment techniques. In large practices or practices with high patient-volume, interviewers[iii] will recruit participants directly in provider's waiting rooms. For in-office recruitment, interviewers spend 2-5 days in providers' waiting rooms, with the goal of recruiting a maximum of 20 parents and/or a maximum of 15 youth for interviews. Interviewers contact all eligible patients to present the study and ask whether they are interested in participating. For those who agree, interviewers obtain informed consent and a copy of the vaccination certificate (if possible) in waiting rooms and then arrange to conduct a telephone interview at a later time. In large group practices, interviewers have the option to work as a team to manage particularly high patient volumes.

For peer review only - http://bmjopen.bmj.com/site/about/guidelines.xhtml

In smaller practices or practices with lower patient volumes for the targeted groups, we ask providers to recruit participants retrospectively by reviewing their patient log from the last 2-6 months and contacting eligible patients to see if they would be interested in participating in the study. Providers transmit a detailed record of attempted contacts and take note of refusals and interested parties. We then contact those who indicate an interest by phone to present the study, obtain informed consent and a copy of the vaccination certificate (if possible) and arrange for a telephone interview. Interviewers select potential participants randomly from the lists provided until they have the required quota of participants in each group.

Interviewers determine the most appropriate recruitment technique during a first visit with the provider. As some providers may have high volumes of one population and low volumes of another (e.g., pediatricians may see many young children but few youths 15 of age or over), we may employ different recruitment methods for the different sub-samples.

Additionally, we conduct a quantitative telephone or face-to-face survey with all participating providers during the quantitative component of the study, in order to quantitatively assess if providers' vaccine perspectives are associated with those of their patients.

Quantitative data analysis

Our primary quantitative research questions are;

1) What are the determinants of vaccine hesitancy for childhood and HPV vaccinations in Switzerland?

2) What is the relationship between vaccine hesitancy and under-immunization?

For peer review only

We measure vaccine hesitancy based on the PACV score. The score will first be validated for Switzerland using Mokken scale analysis to confirm the unidimensionality of the scale. Individuals with a PACV score of ≥50 are considered vaccine hesitant, and individuals with a PACV score <50 are not considered vaccine hesitant (68). We will use multivariate logistic regression to assess the influence of different variables on vaccine hesitancy. Explanatory variables of interest include: age, sex, postal code, household composition, place of birth, immigration status, household income, employment status and work hours, highest achieved level of education, political affiliations, religion, type of school the participants' child/children attend (i.e. public or private school), type of provider the youth consult and parents consult for themselves and their children (i.e. CAM, biomedical), satisfaction with consultations, sources of information about vaccination consulted, decision making process, participants level of trust towards different institutions concerning vaccination issues, CAM usage of parents, their children, and youth, views on parents' role in vaccine decisions, views of importance of health, views of risks of vaccine-preventable diseases, and moral foundations.

For peer review only

Discussion

By using a sequential exploratory mixed methods study design(52), this study will provide rich and multi-faceted data on vaccination decision-making and vaccine hesitancy in Switzerland. A particular strength of this study is its focus on perspectives of both medical providers and their patients. Studies have shown that providers remain a trusted source of information for parents and youth facing vaccine decisions, so a greater understanding of provider perspectives is important(25-32). Another major strength of this study is its inclusion of the perspectives of both CAM

For peer review only - http://bmjopen.bmj.com/site/about/guidelines.xhtml

and biomedical providers. While a growing literature explores the association between vaccine-hesitancy and CAM usage (6, 12, 19, 20), few studies include the perspective of CAM providers and none that we know of has the extent of cooperation with CAM providers found here.

Qualitative interviews provide key insights into parents, youths and CAM and biomedical providers' perspectives on vaccination. In addition, interviews with CAM and biomedical providers shed light onto their views on vaccination, vaccine hesitant patients, and how they interact with this population in their practices. Observations of vaccine consultations in offices provide another opportunity to get a sense of the variety of approaches providers employ when broaching the subject of vaccination. The combination of qualitative interviews with numerous stakeholders in vaccine decision-making and planning, ethnographic observations of vaccination consultations and school information sessions about the HPV vaccination, and qualitative analysis of vaccination information sources allow for qualitative triangulation into the multifaceted phenomenon of vaccine hesitancy in Switzerland. Such a triangulation of qualitative data through multiple qualitative methods allows us to better understand the 'multiple realities' of medical consultations (66), in addition to allowing us to explore how various actors interact with and perceive the Swiss health system in regards to vaccination.

Although data are not strictly representative of Switzerland, data from the quantitative survey will give us a sense of the overall prevalence of vaccine hesitancy in Switzerland and how this varies between language regions, cantons, and urban, suburban, and rural settings. More importantly, it allows us to explore the association of vaccine hesitancy with other factors, like CAM-usage, religion and spirituality, moral foundations, and political orientation. In addition, by pairing data

For peer review only

For peer review only - http://bmjopen.bmj.com/site/about/guidelines.xhtml

from providers and their patients, we can see whether there is an association between providers' and patients' views on vaccines. The quantitative data will also give insight into the major sources of information parents and youth draw on when making vaccination decisions. Finally, the mixed methods study design allowing for the qualitative component to inform the quantitative component also allows us to triangulate both the qualitative and quantitative data gathered throughout the duration of the study so that results from both components can be compared.

Limitations

There are several limitations to the various parts of our studies. A major limitation, which we also argue should be seen as a study strength, comes from our focus on recruiting participants through providers. This technique has major advantages when seeking to examine effects of provider characteristics on patient beliefs and behaviors, particularly for a topic such as vaccine hesitancy where trust between patients and providers has been shown to be a major determinant(32). However, it also indicates that populations that do not regularly see medical providers are under-represented in our data. While universal health insurance requirements attempt to ensure that most people living in Switzerland have access to health care, certain populations are still under-represented, including recent migrants and undocumented populations. Recruiting through providers poses further questions of selection bias, as people who see doctors more often are more likely to participate in our study. This bias means that, for example, parents of infants were more apt to be recruited than parents of older children, as infants see the doctor more regularly. Selection bias problems are particularly relevant to youth populations, as most youth are healthy and thus among the least likely to regularly

For peer review only

seek medical care. As such, youth in our study may differ in important ways from youth in Switzerland overall. In our view, the advantages of being able to link provider and patient data outweighed these limitations.

For qualitative interviewing, interviewer effects may affect the type and quality of information we received; interviews and observations are conducted by a sociologist, a medical doctor trained in qualitative research methods, a senior medical student with qualitative research training, and a medical anthropologist. That being said, having a variety of researchers with different backgrounds involved in the data collection and analytical process adds to the richness of the analysis through clarification of concepts during in-depth research team discussions. Another limitation arises from the way we observed vaccine discussions. Observers watch the discussion in person and take notes, but do not audio or video record discussions. The absence of such recordings limits our ability to rigorously analyze the conversations, while the physical presence of observers in the consultation may have had an effect on discussions. Furthermore, given that the data are collected in both French-speaking and German-speaking regions of Switzerland, we have a multilingual corpus which will necessitate in-depth discussions between study members during qualitative data analysis.

Furthermore, as noted above, the quantitative study will not be based on a representative sample but will instead allow comparing results between different types of providers and their patients, and between different socio-cultural regions of Switzerland. As in the qualitative component, interviewer effects may affect the type and quality of information we received. We will include data quality controls into data analysis in order to check for interviewer effects. Additionally, linguistic barriers,

For peer review only

For peer review only - http://bmjopen.bmj.com/site/about/guidelines.xhtml

particularly with recent migrants to Switzerland, may prove challenging for participants to provide accurate responses to our interview questions.

Scientific relevance

Vaccine hesitancy is a complex public health issue in high-income countries, yet there is no agreed upon measure of vaccine hesitancy. Multilingual, validated instruments for measuring vaccine hesitancy are rare, making comparative study difficult. This study develops and test translations of the 15-item PACV in three new languages and provides the valuable assessments of vaccine hesitancy in French-, German- and Italian-speaking contexts. Furthermore, our study design includes vaccination certificates in order to statistically examine the relationship between vaccine hesitancy and under-immunization.

Results from the study will also make a contribution to the literature on the relationship between CAM-usage and vaccine hesitancy. The combination of rich qualitative data including both biomedical and CAM perspectives and provider-linked data from patients who see CAM-practitioners will allow a more in-depth examination of this relationship than found in the literature to date.

A combination of the data collected through the quantitative and qualitative methods and their analysis will form the necessary background for the design, submission, and implementation of an appropriate intervention randomized control trial intervention designed at improving vaccine communication and counseling among physicians, parents, and adolescents in Switzerland.

Patient and public involvement

Study participants, both patients and medical providers, are involved throughout the duration of the data collection. Study tools are piloted and tested with the research

For peer review only - http://bmjopen.bmj.com/site/about/guidelines.xhtml

team requesting patient and provider input during this process. Participants are allowed the opportunity to indicate any additional comments throughout data collection so that we may take their priorities, experiences, and preferences into account. Patients (parents and adolescents) and providers assist us in additional recruitment efforts as described in the methods section. All participants were informed of the amount of time their participation would entail prior to data collection. We systematically request participants' wishes to receive the results of the study by including an item on the consent form allowing them to provide us with their contact information. We will send relevant published results to study participants and invite them to public forums where we discuss our study results.

Ethics and dissemination

The study is conducted in compliance with this study protocol, the Swiss Federal Act on Research Involving Human Beings (Human research Act, HRA), and the Declaration of Helsinki. The study was approved by the local ethics committee (Ethikkommission Nordwest- und Zentralschweiz, EKNZ; project ID number 2017-00725); ethics approval covers all study participants, including adolescent and adult patients, as well as biomedical and CAM providers. All study participants receive information about the study and provide written informed consent. In accordance with the Human Research Act, adolescents (defined as individuals 14 years of age or older) who are capable of judgement are able to provide informed consent in writing and do not need to provide informed consent in writing from a legal representative "if the research project entails more than minimal risks and burdens" (HRA, Art. 23, Par. 1). The EKNZ has approved of this study as entailing less than minimal risks and burdens and thus does not require informed consent in writing from a legal representative for adolescents. Informed consent from minor

For peer review only

For peer review only - http://bmjopen.bmj.com/site/about/guidelines.xhtml

participants (all aged 15 years and older for this study) is obtained directly from the participants and not their legal representatives.

Study results will be published in peer-reviewed academic journals and disseminated to healthcare professionals, researchers, and the public via academic conferences and public presentations. The datasets used and/or analyzed during the current study are available from the corresponding author on reasonable request.

References

1. Centers for Disease C, Prevention. Ten great public health achievements-- United States, 1900-1999. MMWR Morb Mortal Wkly Rep. 1999;48(12):241-3.
2. Maglione MA, Das L, Raaen L, Smith A, Chari R, Newberry S, et al. Safety of Vaccines Used for Routine Immunization of US Children: A Systematic Review. Pediatrics. 2014.
3. Thompson KM, Lewis Odahowski C. Systematic Review of Health Economic Analyses of Measles and Rubella Immunization Interventions. Risk Analysis. 2014;36(7):1297-314. doi: 10.111/risa.12331.
4. Larson HJ, Jarrett C, Eckersberger E, Smith DMD, Paterson P. Understanding vaccine hesitancy around vaccines and vaccination from a global perspective: A systematic review of published literature, 2007–2012. Vaccine. 2014;32(19):2150-9.
5. Omer SB, Salmon DA, Orenstein WA, deHart MP, Halsey N. Vaccine Refusal, Mandatory Immunization, and the Risks of Vaccine-Preventable Diseases. N Engl J Med. 2009;360(19):1981-8.
6. Peretti-Watel P, Larson HJ, Ward JK, Schulz WS, Verger P. Vaccine Hesitancy: Clarifying a Theoretical Framework for an Ambiguous Notion. PLoS Currents. 2015;7:ecurrents.outbreaks.6844c80ff9f5b273f34c91f71b7fc289.
7. Eskola J, Duclos P, Schuster M, MacDonald NE. How to deal with vaccine hesitancy? Vaccine. 2015;33(34):4215-7.
8. Larson HJ, Jarrett C, Schulz WS, Chaudhuri M, Zhou Y, Dube E, et al. Measuring vaccine hesitancy: The development of a survey tool. Vaccine. 2015;33(34):4165-75.
9. World Health O. WHO | Vaccine hesitancy: A growing challenge for immunization programmes. WHO. 2015.
10. WHO. Report of the SAGE Working Group on Vaccine Hesitancy. 2014.
11. WHO. Ten threats to global health in 2019 2019 [Available from: https://www.who.int/emergencies/ten-threats-to-global-health-in-2019.
12. Poland CM, Brunson EK. The need for a multi-disciplinary perspective on vaccine hesitancy and acceptance. Vaccine. 2015;33(2):277-9.
13. Brunson EK. The Impact of Social Networks on Parents' Vaccination Decisions. Pediatrics. 2013;131(5):e1397-404.

For peer review only

For peer review only - http://bmjopen.bmj.com/site/about/guidelines.xhtml

14. Betsch C, Sachse K. Dr. Jekyll or Mr. Hyde? (How) the Internet influences vaccination decisions: Recent evidence and tentative guidelines for online vaccine communication. Vaccine. 2012;30(25):3723-6.

15. Kata A. A Postmodern Pandora's Box: Anti-Vaccination Misinformation on the Internet. Vaccine. 2010;28(7):1709-16.

16. Yaqub O, Castle-Clarke S, Sevdalis N, Chataway J. Attitudes to vaccination: A critical review. Social Science & Medicine. 2014;112:1-11.

17. Wang E, Baras Y, Buttenheim AM. “Everybody just wants to do what's best for their child”: Understanding how pro-vaccine parents can support a culture of vaccine hesitancy. Vaccine. 2015;33(48):6703-9.

18. Sobo EJ, Huhn A, Sannwald A, Thurman L. Information Curation among Vaccine Cautious Parents: Web 2.0, Pinterest Thinking, and Pediatric Vaccination Choice. Medical Anthropology. 2016;35(6):529-46.

19. Browne M, Thomson P, Rockloff MJ, Pennycook G. Going against the Herd: Psychological and Cultural Factors Underlying the ‘Vaccination Confidence Gap’. PLOS ONE. 2015;10(9):e0132562.

20. Wardle J, Frawley J, Steel A, Sullivan E. Complementary medicine and childhood immunisation: A critical review. Vaccine. 2016;34(38):4484-500.

21. Gray JM. Postmodern medicine. 1999;354(9189):1550-3.

22. Beck U, Ritter M. Risk society: towards a new modernity. London: Sage Publications; 1992.

23. Greenhalgh T, Wessely S. ‘Health for me’: a sociocultural analysis of healthism in the middle classes. British Medical Bulletin. 2004;69(1):197-213.

24. Armstrong D. Actors, patients and agency: a recent history. Sociology of Health & Illness. 2014;36(2):163-74.

25. Salmon DA, Pan WKY, Omer SB, Navar AM, Orenstein W, Marcuse EK, et al. Vaccine knowledge and practices of primary care providers of exempt vs. vaccinated children. Human Vaccines. 2008;4(4):286-91.

26. Opel DJ, Heritage J, Taylor JA, Mangione-Smith R, Salas HS, DeVere V, et al. The Architecture of Provider-Parent Vaccine Discussions at Health Supervision Visits. Pediatrics. 2013;132(6):1037.

27. Verger P, Fressard L, Collange F, Gautier A, Jestin C, Launay O, et al. Vaccine Hesitancy Among General Practitioners and Its Determinants During Controversies: A National Cross-sectional Survey in France. EBioMedicine. 2015;2(8):891-7.

28. Kimmel SR, Burns IT, Zimmerman RK. Addressing immunization barriers, benefits, and risks. 2003;52(1 Suppl):S47.

29. Bryant KA, Wesley GC, Wood JA, Hines C, Marshall GS. Use of standardized patients to examine physicians’ communication strategies when addressing vaccine refusal: A pilot study. Vaccine. 2009;27(27):3616-9.

30. Opel DJ, Robinson JD, Heritage J, Korfiatis C, Taylor JA, Mangione-Smith R. Characterizing providers’ immunization communication practices during health supervision visits with vaccine-hesitant parents: A pilot study. Vaccine. 2012;30(7):1269-75.

31. Paterson P, Meurice F, Stanberry LR, Glismann S, Rosenthal SL, Larson HJ. Vaccine hesitancy and healthcare providers. Vaccine. 2016;34(52):6700-6.

32. Benin AL, Wisler-Scher DJ, Colson E, Shapiro ED, Holmboe ES. Qualitative analysis of mothers' decision-making about vaccines for infants: the importance of trust. American Academy of Pediatrics. 2006;117(5):1532-41.

For peer review only - http://bmjopen.bmj.com/site/about/guidelines.xhtml

33. FOPH. Durchimpfung von 2-, 8- und 16-Jährigen in der Schweiz, 2011 bis 2013. 2015(28):538-43.
34. Masserey Spicher V. The Federal Vaccination Commission in Switzerland: An officially appointed independent commission ensuring evidence-based recommendations and transparent procedures. Vaccine. 2010;28:A48-A53.
35. Bundesamt für Gesundheit BO. Durchimpfung von 2-, 8- und 16-Jährigen in der Schweiz, 2011 bis 2013. BAG Bulletin; 2015.
36. Lang P, Zimmermann H, Piller U, Steffen R, Hatz C. The Swiss National Vaccination Coverage Survey, 2005-2007. Public Health Reports (1974-). 2011;126:97-108.
37. FOPH. Couverture vaccinale des enfants âgés de 2, 8 et 16 ans en Suisse, 2014–2016. FOPH Bulletin: FOPH; 2018. Contract No.: 24.
38. Bundesamt für Gesundheit BO. Die HPV-Impfung in der Schweiz: Resultate einer nationalen Befragung im Jahr 2014. BAG Bulletin. 2015(23):445-52.
39. Riesen M, Garcia V, Low N, Althaus CL. Modeling the consequences of regional heterogeneity in human papillomavirus (HPV) vaccination uptake on transmission in Switzerland. bioRxiv. 2017:125518.
40. Riesen M, Konstantinoudis G, Lang P, Low N, Hatz C, Maeusezahl M, et al. Exploring variation in human papillomavirus vaccination uptake in Switzerland: a multilevel spatial analysis of a national vaccination coverage survey. BMJ Open. 2018;8(5):e021006.
41. Richard JL, Masserey Spicher V. Large measles epidemic in Switzerland from 2006 to 2009: consequences for the elimination of measles in Europe. Euro Surveillance. 2009;14(50):pii-19443.
42. Altpeter E, Wymann MN, Richard J-L, Mäusezahl-Feuz M. Marked increase in measles vaccination coverage among young adults in Switzerland: a campaign or cohort effect? International Journal of Public Health. 2018;63(5):589-99.
43. Bundesamt für Gesundheit BO. Neue Welle der Masernepidemie Anfang 2009: Beschreibung und Massnahmen. BAG Bulletin. 2009(27):484-91.
44. Zuzak TJ. Attitudes towards vaccination: users of complementary and alternative medicine versus non-users. Swiss Med Wkly. 2008;138.
45. Klein SD, Torchetti L, Frei-Erb M, Wolf U. Usage of Complementary Medicine in Switzerland: Results of the Swiss Health Survey 2012 and Development Since 2007. PLOS ONE. 2015;10(10):e0141985.
46. Simões-Wüst AP, Rist L, Dettling M. Self-Reported Health Characteristics and Medication Consumption by CAM Users and Nonusers: A Swiss Cross-Sectional Survey. Journal of Alternative and Complementary Medicine. 2014;20(1):40-7.
47. Wolf U, Maxion-Bergemann S, Bornhöft G, Matthiessen P, Wolf M. Use of Complementary Medicine in Switzerland. Complementary Medicine Research. 2006;13(suppl 2)(s02):4-6.
48. Hart J. Complementary and Alternative Medicine Use and Initiatives in Europe. Alternative and Complementary Therapies. 2017;24(1):32-4.
49. FOPH. Médecines complémentaires pratiquées par des médecins 2018 [Available from: https://www.bag.admin.ch/bag/fr/home/themen/versicherungen/krankenversicherung/krankenversicherung-leistungen-tarife/Aerztliche-Leistungen-in-der-Krankenversicherung/Aerztliche-Komplementaermedizin.html.

For peer review only

For peer review only - http://bmjopen.bmj.com/site/about/guidelines.xhtml

50. FOPH. Plan de vaccination suisse 2019 [Available from: https://www.bag.admin.ch/bag/fr/home/gesund-leben/gesundheitsfoerderung-und-praevention/impfungen-prophylaxe/schweizerischer-impfplan.html.

51. Rosenfield PL. The potential of transdisciplinary research for sustaining and extending linkages between the health and social sciences. Social Science & Medicine. 1992;35(11):1343-57.

52. Creswell JW, Plano Clark VL. Designing and Conducting Mixed Methods Research. 2 ed. Los Angeles
London
New Delhi
Singapore
Washington D.C.: SAGE; 2011. 443 p.

53. Morse JM. The Significance of Saturation. Qualitative Health Research. 1995;5(2):147-9.

54. Nelson J. Using conceptual depth criteria: addressing the challenge of reaching saturation in qualitative research. Qualitative Research. 2017;17(5):554-70.

55. Y B-D, C A, A B, D M, B L, Z T, et al. Human Papillomavirus (HPV) Vaccine in Switzerland: The reasons why some young women do not get vaccinated. Joint Annual Meeting of the Swiss Society of Paediatrics, Swiss Society of Paediatric Surgery and Swiss Society of Child and Adolescent Psychiatry and Psychotherapy; May 24-25, 2018; Lausanne, Switzerland2018.

56. Braun V, Clarke V. Using thematic analysis in psychology. Qualitative Research in Psychology. 2006;3(2):77-101.

57. Fairclough N. Critical Discourse Analysis: The Critical Study of Language. London: Longman; 1995 1995. 304 p.

58. Van Dijk TA. Principles of critical discourse analysis. 1993;4(2):249-83.

59. van Dijk TA. Critical Discourse Analysis. The Handbook of Discourse Analysis: Blackwell Publishers Ltd; 2008. p. 349-71.

60. Stahl JP, Cohen R, Denis F, Gaudelus J, Martinot A, Lery T, et al. The impact of the web and social networks on vaccination. New challenges and opportunities offered to fight against vaccine hesitancy. Médecine et Maladies Infectieuses. 2016;46(3):117-22.

61. Witteman HO, Zikmund-Fisher BJ. The defining characteristics of Web 2.0 and their potential influence in the online vaccination debate. Vaccine. 2012;30(25):3734-40.

62. Betsch C, Brewer NT, Brocard P, Davies P, Gaissmaier W, Haase N, et al. Opportunities and challenges of Web 2.0 for vaccination decisions. Vaccine. 2012;30(25):3727-33.

63. Gale N, Heath G, Cameron E, Rashid S, Redwood S. Using the framework method for the analysis of qualitative data in multi-disciplinary health research. BMC Medical Research Methodology. 2013;13(1):117.

64. Charmaz K. Constructing Grounded Theory: A Practical Guide through Qualitative Analysis. 1st ed. London, Thousand Oaks, New Delhi: SAGE Publications; 2006. 208 p.

65. Bowen GA. Grounded Theory and Sensitizing Concepts. International Journal of Qualitative Methods. 2006;5(3):12-23.

66. Barry CA. Multiple Realities in a Study of Medical Consultations. Qualitative Health Research. 2002;12(8):1093-111.

67. Deml M, Notter J, Kliem P, Buhl A, Huber BM, Pfeiffer C, et al. “We treat humans, not herds!”: A qualitative study of complementary and alternative medicine (CAM) providers’

For peer review only - http://bmjopen.bmj.com/site/about/guidelines.xhtml

individualized approaches to vaccination in Switzerland. Social Science & Medicine. Under Review.

68. Opel DJ, Taylor JA, Zhou C, Catz S, Myaing M, Mangione-Smith R. The relationship between parent attitudes about childhood vaccines survey scores and future child immunization status: A validation study. JAMA Pediatrics. 2013;167(11):1065-71.

69. Amin AB, Bednarczyk RA, Ray CE, Melchiori KJ, Graham J, Huntsinger JR, et al. Association of moral values with vaccine hesitancy. Nature Human Behaviour. 2017;1(12):873-80.

70. Bundesamt für Gesundheit BO. Masernimpfung in der Schweiz im 2012: Resultate einer nationalen Bevölkerungsbefragung zum Thema Masern. BAG Bulletin. 2013(17):278-83.

Figure Legends

Figure 1: Network of participating providers

Author Statement

Authors' contributions

MD co-drafted the manuscript and focused on the qualitative components. KJ co-drafted the manuscript and focused on the quantitative components. SM initiated the drafting of the manuscript and provided valuable feedback during its writing. BH assisted in establishing a network of complementary and alternative medical (CAM) practitioners and provided expertise in both pediatrics and CAM in the Swiss context. AB participated in developing the methods for the qualitative research specific to HPV. EF, VM, JS, PK, and RC coordinated quantitative data collection efforts and were instrumental in the preparation of the study's quantitative tools and data management. DK participated regularly in study advisory board meetings and provided insight into CAM perspectives and feedback about the study design. CP took a lead role in establishing the study's qualitative methodologies. CBJ provided regular study supervision and inputs about the qualitative results. PET is the principal investigator, directed the funding request, and supervised the conduct of

For peer review only

the study in its entirety. He provided infectious disease and general medical expertise, oversaw study conception, design, data collection, analysis, and interpretation. All authors read, contributed to, and approved the final manuscript.

Funding statement

The study is funded in its entirety via the Swiss National Science Foundation, in the setting of National Research Program (NRP) 74. No funding was obtained from vaccine manufacturers or the Federal Office of Public Health.

Competing interests statement

Our research team attests that there is no conflict of interest and that our study will be conducted independently of outside influences in terms of specific intellectual, financial, and proprietary agendas.

Data Statement

The datasets used and/or analyzed during the current study are available from the corresponding author on reasonable request.

Abbreviations

CAM: Complementary and alternative medicine; FGD: Focus group discussion; EKNZ: Ethikkommission Nordwest- und Zentralschweiz; FOPH: Swiss Federal Office of Public Health; HPV: Human papillomavirus; Human Research Act: HRA; PACV: Parent Attitudes about Child Vaccine; SAGE: Strategic Advisory Group of Experts; TCM: Traditional Chinese Medicine; WHO: World Health Organization

For peer review only

Acknowledgments

The authors would like to thank Christoph Berger (University Children's Hospital Zurich), Andreas Zeller (University Institute of Primary Care Medicine Basel) and Mirjam Mäusezahl (Epidemiology Section, Federal Office of Public Health, Bern) for their important contributions to protocol development and for many helpful discussions. Some of the study's methods and preliminary findings have previously been presented at the conferences listed below:

- Notter J., Deml M, Huber B, Krüerke D, Jafflin K, Zeller A, Mäusezahl M, Berger C, Merten S, Burton-Jeangros C, Pfeiffer C, Tarr PE. Poster presentation at SSI (Swiss Society for Infectious Diseases) Joint Annual Meeting in Interlaken, 14 September 2018; "Complementary and alternative medical (CAM) providers' patient-centered, individualized approaches to vaccination in Switzerland"
- Tarr P. Oral presentation at Global challenges in vaccine acceptance science and programs, 24 - 26 September 2018 - Les Pensières Center for Global Health, Veyrier du Lac, France; "Approaches of complementary and biomedical providers to vaccinations in Switzerland"
- Deml M. Oral presentation at Swiss Public Health Conference in Neuchâtel, 8 November 2018; "Vaccination Policy, Vaccine Uptake, and Vaccine Hesitancy in Switzerland"

Word count: 9732

For peer review only

---

[i] *Basic* vaccines are defined as "essential to individual and public health, and offer a level of protection that is indispensable to people's well-being (e.g., diphtheria, tetanus, pertussis, polio, MMR, HBV, HPV)." (p. A51) *Complementary vaccines* are defined as being able to "maximize individual protection and are meant for individuals seeking protection from well-defined risks (e.g., conjugate pneumococcal vaccine and conjugate meningococcus C vaccine)." (p. A51)

For peer review only - http://bmjopen.bmj.com/site/about/guidelines.xhtml

ii 51. Rosenfield PL. The potential of transdisciplinary research for sustaining and extending linkages between the health and social sciences. Social Science & Medicine. 1992;35(11):1343-57. explains how *transdisciplinary research* can be conducted, particularly in health research: “Representatives of different disciplines are encouraged to transcend their separate conceptual, theoretical, and methodological orientations in order to develop a shared approach to the research, building on a common conceptual framework. Such a framework can be used to define and analyze the research problem and develop new approaches for health care that more closely represent the historical and present-day reality in which health problems are situated” (p. 1351).

iii Interviewers are medical students trained in recruiting participants, obtaining informed consent, conducting quantitative interviews over the telephone, and quantitative data management.

For peer review only

For peer review only - http://bmjopen.bmj.com/site/about/guidelines.xhtml

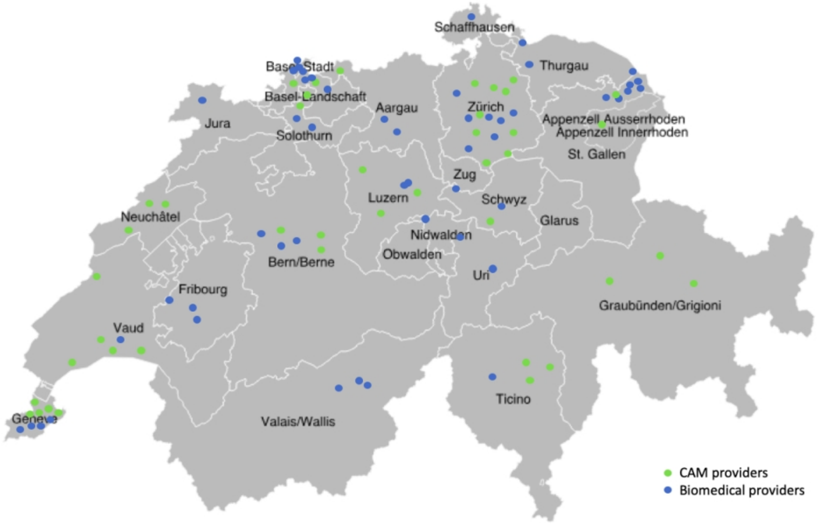

Figure 1 - Network of participating providers

503x269mm (72 x 72 DPI)

Formatting checklist

1. **Author information:** Have you got an ORCID iD (all BMJ journals publishing primarily original research now mandate ORCID iDs for all submitting authors at the time of article submission). **Yes. However, we were unable to link the authors to their ORCID iDs (listed below) through the online platform because we did not have this option.** Have you provided details of all of your co-authors? **Yes.** Is the information that you have entered into ScholarOne the same as the information on the manuscript title page? **Yes.**

Michael Deml
michael.deml@swisstph.ch
https://orcid.org/0000-0003-2224-8173

Kristen Jafflin
kristen.jafflin@swisstph.ch
https://orcid.org/0000-0003-2105-4262

Sonja Merten
sonja.merten@swisstph.ch
https://orcid.org/0000-0003-4115-106X

Benedikt Huber
Benedikt.Huber@h-fr.ch
https://orcid.org/0000-0003-2952-3879

Andrea Buhl
andrea.buhl@unibas.ch
https://orcid.org/0000-0002-7907-7902

Eleonora Frau
eleonora.frau@stud.unibas.ch
https://orcid.org/0000-0003-4040-5937

Valérie Mettraux
valerie.mettraux@stud.unibas.ch
https://orcid.org/0000-0003-4065-428X

Joanna Sonderegger
joanna.sonderegger@stud.unibas.ch
https://orcid.org/0000-0001-8003-3176

Paulina Kliem
paulina.kliem@stud.unibas.ch

For peer review only

https://orcid.org/0000-0003-3521-5880

Rachele Cattalani
rachele.cattalani@stud.unibas.ch
https://orcid.org/0000-0002-8581-7890

Daniel Krüerke
daniel.krueerke@klinik-arlesheim.ch
https://orcid.org/0000-0002-3600-0403

Constanze Pfeiffer
constanze.pfeiffer@unibas.ch
https://orcid.org/0000-0002-9487-2867

Claudine Burton-Jeangros
claudine.jeangros@unige.ch
https://orcid.org/0000-0003-2258-9728

Philip E. Tarr
philip.tarr@unibas.ch
https://orcid.org/0000-0002-1488-5407

2. **Manuscript length and formatting**: Have you provided your abstract in the correct format? **Yes/** Have you supplied any required additional information for your article type, such as key messages? **N/A** Have you checked that your manuscript doesn't exceed the requirements for word count, number of tables and/or figures, and number of references? **Yes. The manuscript exceeds the word count, and we have noted this in the cover letter to the editor.**

3. **Tables**: Are your tables in an editable format? Have you embedded them into the main word document? Have they been cited in the text? Have you provided appropriate table legends? Have you uploaded any lengthy tables as supplementary files for online publication? **N/A**

4. **Figures**: Have you uploaded figures separately from the text? **Yes. As indicated in the online platform, we have included the figure at the end of the PDF document of the manuscript.** Have they been supplied in an acceptable format and are they of sufficient quality? **Yes.** Are they suitable for black and white reproduction (unless you intend to pay any required fees for colour printing)? **No.**

For peer review only

The figure is colour dependent. We could pay additional required fees for colour printing, if necessary. Have the files been labelled appropriately? **Yes.** Have the figures been cited in the text? **Yes.** Have you provided appropriate figure legends? **YES. Lines 960-961 of the submitted PDF.**

5. **References**: Have all of the references been cited in the text? **Yes.**

6. **Supplementary files**: Have you supplied these in an acceptable format? **Yes.** Have they been cited in the main text? **N/A. The supplementary files we have included are budgetary documents, ethics approvals, and a manuscript reporting initial qualitative results that we have submitted to Social Science & Medicine for the editor to review.**

7. **Statements**: Have you included the necessary statements relating to author contributorship, competing interests and funding, data sharing, patient consent and ethical approval? **YES.**

8. **Acknowledgements:** Have you acknowledged all contributors who do not meet the criteria for authorship? **YES.** Have you acknowledged if your work has been previously presented at a conference/published as a conference abstract? **YES. This is indicated in the submitted manuscript (lines 1010 – 1022).**

9. **Suggested reviewers:** Have you suggested reviewers for your paper (if required by the journal)? **We suggest the reviewers below:**

   Eve Dubé, Eve.Dube@inspq.qc.ca
   Pierre Verger, pierre.verger@inserm.fr
   Suzanne Suggs, suzanne.suggs@usi.ch
   Alessandro Diana, Alessandro.Diana@hcuge.ch

For peer review only

10. **Research reporting checklists**: Have you either provided the appropriate statement for your study type, or explained why a checklist isn't required? **N/A**

11. **Reproducing figures:** Have you obtained permission from the copyright holder to re-use any previously published material? Has the source been acknowledged? **N/A**

For peer review only

BMJ Open

Determinants of Vaccine Hesitancy in Switzerland - Study Protocol of a Mixed Methods National Research Program

Journal:	*BMJ Open*
Manuscript ID	bmjopen-2019-032218.R1
Article Type:	Protocol
Date Submitted by the Author:	21-Aug-2019
Complete List of Authors:	Deml, Michael; Swiss TPH, Dept. Epidemiology & Public Health (EPH); University of Basel<br>Jafflin, Kristen; Swiss Tropical and Public Health Institute; University of Basel<br>Merten, Sonja; Swiss Tropical and Public Health Institute; University of Basel<br>Huber, Benedikt; HFR Fribourg Cantonal Hospital, Department of Pediatrics<br>Buhl, Andrea; Swiss Tropical and Public Health Institute; University of Basel<br>Frau, Eleonora ; University of Basel; University Department of Medicine, Kantonsspital Baselland, University of Basel<br>Mettraux, Valérie; University of Basel; University Department of Medicine, Kantonsspital Baselland, University of Basel<br>Sonderegger, Joanna; University of Basel; University Department of Medicine, Kantonsspital Baselland, University of Basel<br>Kliem, Paulina; University of Basel; University Department of Medicine, Kantonsspital Baselland, University of Basel<br>Cattalani, Rachele; University of Basel; University Department of Medicine, Kantonsspital Baselland, University of Basel<br>Krüerke, Daniel; Klinik Arlesheim AG<br>Pfeiffer, Constanze; Swiss Tropical and Public Health Institute; University of Basel<br>Burton-Jeangros, Claudine; Institute of Sociological Research, University of Geneva<br>Tarr, Philip ; University of Basel; University Department of Medicine, Kantonsspital Baselland, University of Basel
**Primary Subject Heading**:	Infectious diseases
Secondary Subject Heading:	General practice / Family practice, Epidemiology, Health services research, Patient-centred medicine, Public health
Keywords:	Vaccine hesitancy, Under-immunization, Childhood vaccinations, HPV vaccination, Complementary and alternative medicine (CAM), Patient-provider communication

Title: Determinants of Vaccine Hesitancy in Switzerland - Study Protocol of a Mixed Methods National Research Program

Authors:
Michael Deml [a,b*]
michael.deml@swisstph.ch
Kristen Jafflin [a,b*]
kristen.jafflin@swisstph.ch
Sonja Merten [a,b*]
sonja.merten@swisstph.ch
Benedikt Huber [d]
Benedikt.Huber@h-fr.ch
Andrea Buhl [a,b]
andrea.buhl@unibas.ch
Eleonora Frau [b,c]
eleonora.frau@stud.unibas.ch
Valérie Mettraux [b,c]
valerie.mettraux@stud.unibas.ch
Joanna Sonderegger [b,c]
joanna.sonderegger@stud.unibas.ch
Paulina Kliem [b,c]
paulina.kliem@stud.unibas.ch
Rachele Cattalani [b,c]
rachele.cattalani@stud.unibas.ch
Daniel Krüerke [e]
daniel.krueerke@klinik-arlesheim.ch
Constanze Pfeiffer [a,b]
constanze.pfeiffer@unibas.ch
Claudine Burton-Jeangros [f]
claudine.jeangros@unige.ch
Philip E. Tarr [b,c]
philip.tarr@unibas.ch

[a]Swiss Tropical and Public Health Institute (Swiss TPH); Socinstrasse 57; 4051 Basel, Switzerland
[b]University of Basel; Petersplatz 1; 4051 Basel, Switzerland
[c]University Department of Medicine, Kantonsspital Baselland, University of Basel; 4101 Bruderholz, Switzerland
[d]Department of Pediatrics, HFR Fribourg – Hôpital cantonal; Chemin des Pensionnats 2-6; 1708 Fribourg, Switzerland
[e]Resrach Department, Klinik Arlesheim; Pfeffingerweg 1, 4144 Arlesheim, Switzerland
[f]Institute of Sociological Research, University of Geneva; Boulevard du Pont-d'Arve 40; 1204 Geneva, Switzerland

** these authors contributed equally to the manuscript*

Corresponding author: Prof. Dr. med. Philip Tarr, University Department of Medicine, Kantonsspital Baselland, University of Basel; CH-4101 Bruderholz, Switzerland, Philip.tarr@unibas.ch, Phone +41 61 436 2212

For peer review only - http://bmjopen.bmj.com/site/about/guidelines.xhtml

Abstract

Introduction: Vaccine hesitancy is a complex public health issue referring to concerns about the safety, efficacy or need for vaccination. Relatively little is known about vaccine hesitancy in Switzerland. This ongoing study (2017-2021) focuses on biomedical and complementary and alternative medicine (CAM) providers and their patients since healthcare professionals play important roles in vaccination decision-making. This national research program seeks to assess the socio-cultural determinants of vaccine hesitancy regarding childhood and human papilloma virus (HPV) vaccines in Switzerland. We aim to provide a detailed characterization of vaccine hesitancy, including CAM and biomedical perspectives, patient-provider interactions, and sociocultural factors, to establish mediating effects of vaccine hesitancy on under-immunization, and to design an intervention to improve vaccination communication and counseling among physicians, parents, and adolescents.

Methods and analysis: Our transdisciplinary team employs a sequential exploratory mixed-methods study design. We have established a network of more than 150 medical providers across Switzerland, including more than 40 CAM practitioners. For the qualitative component, we conduct interviews with parents, youth, biomedical and CAM providers and observations of vaccination consultations and school vaccination information sessions. For the quantitative component, a sample of 1,350 parents of young children and 722 young adults (15-26 years) and their medical providers respond to questionnaires. We measure vaccine hesitancy with the Parent Attitudes about Child Vaccine 15-item survey and review vaccination certificates to assess vaccination status. We administer additional questions based on findings from qualitative research, addressing communication with medical providers, vaccine

For peer review only - http://bmjopen.bmj.com/site/about/guidelines.xhtml

information sources, and perceptions of risk control *vis-à-vis* vaccine preventable diseases. The questionnaires capture sociodemographics, political views, religion and spirituality, and moral foundations.

Ethics and dissemination: The study was approved by the local ethics committee. The results will be published in peer-reviewed journals and disseminated to healthcare professionals, researchers, and the public via conferences and public presentations.

Article Summary

Strengths and limitations of this study:

- A novelty of this research includes its large sample of CAM and biomedical providers, with consideration given to their vaccination perspectives and interactions with their patients. The study emphasis on CAM providers is important since previous studies have shown an association between CAM use and vaccine hesitancy as well as high patient demand for CAM in Switzerland.
- The sequential exploratory mixed-method study design and transdisciplinary nature of this study provide further insights into the relationships between CAM and biomedical providers and vaccine hesitancy and uptake, with the qualitative methods and results having informed the design of the quantitative questionnaire.
- Although the methodological approach to recruit patients via medical providers is advantageous because it allows for the examination of effects of provider characteristics on patient vaccination beliefs and behaviors, it limits our results because populations that do not regularly see medical providers

For peer review only

are under-represented in our data. Furthermore, the data do not provide nationally representative results.

Key words

- Vaccine hesitancy
- Under-immunization
- Childhood vaccinations
- HPV vaccination
- Complementary and alternative medicine (CAM)
- Mixed methods research
- Patient-provider communication
- Vaccination information sources

Introduction

Vaccines are ranked among the greatest public health achievements(1). Extensive evidence documents the efficacy, minimal side-effects, and cost-effectiveness of vaccines recommended by numerous national immunization programs, including that of the Swiss Federal Office of Public Health (FOPH)(2, 3). Despite public health successes of vaccines, a large number of people in Western countries express concerns about vaccine safety, efficacy or need, a behavior now referred to as *vaccine hesitancy*(4-6).

Vaccine hesitancy has gained increasing attention from the global public health community. In August 2015, the World Health Organization (WHO) referred to vaccine hesitancy as a “growing challenge for immunization programs” (7-9), responding to a 2014 report(10) from the WHO Strategic Advisory Group of Experts (SAGE) on immunization. In early 2019, WHO listed vaccine hesitancy among the top 10 threats to global health, citing a global need to address rising rates of measles and to eliminate cervical cancer by ramping up efforts to increase coverage with the human papillomavirus (HPV) vaccine(11).

While the importance of vaccine hesitancy is widely acknowledged, its determinants are incompletely understood and merit further investigation(4).

For peer review only - http://bmjopen.bmj.com/site/about/guidelines.xhtml

Researchers having conducted a systematic review on determinants of vaccine hesitancy from a global perspective found that there was "no universal algorithm" (p. 2155) and recommended context-specific research(4). Other researchers have argued in favor of cross-disciplinary research, stating, "Vaccine hesitancy and acceptance are complex, multi-faceted issues. Our understanding of them must be complex and multifaceted as well" (p. 279)(12).

When vaccine hesitancy leads to *under-immunization,* which we define as delaying or omitting some or all officially recommended vaccines, this provides an example of the underuse of a medical intervention considered to be safe and effective. Under-immunization is a public health concern because it can reduce population protection, also referred to as *herd immunity*, towards certain potentially dangerous infectious agents and increases the risk of disease outbreaks.

Studies suggest that personal, social(12, 13) and local cultural(6) networks are important determinants of vaccination decisions, in particular among those who under-vaccinate. Furthermore, people's sources of information on health and vaccination-related information can play important roles in vaccination choices, with recent research pointing to the importance of 'information overload,' 'misinformation,' and higher levels of indecision among parents, particularly given the wide availability of (mis)information on the Internet(14-18).

Additionally, parent and provider perspectives that differ from official public health recommendations need to be considered in order to understand vaccine hesitancy(12). These may include intuitive rather than analytical cognitive styles, reluctance to consider the evidence suggesting vaccine safety/efficacy, and lowered willingness to trust information delivered by public health authorities, as shown in an adherence to complementary and alternative medicine (CAM)-oriented health values

For peer review only - http://bmjopen.bmj.com/site/about/guidelines.xhtml

and preference of CAM over biomedicine(19). Studies also suggest that under-immunization and usage of CAM are determined by similar factors, such as alternative concepts of body, immunity, risk perception, anti-authoritarian orientation, and distrust of biomedical establishment and the pharmaceutical industry(6, 19). Some of these associations may in part be due to confounding factors, such as higher income and education and distrust of medical systems(20). Other sociomedical trends may also play a role in heightened patient participation in decision-making, such as postmodern medicine(21), characterized by risk culture(22), healthism(23), and patients activating more agency in healthcare decisions(24). Finally, research findings suggest limitations to the efficacy of the evidence-based strategies that have generally employed by health authorities over the past decades to increase vaccination rates(19).

A key determinant of patient vaccination decision-making seems to be the attitudes, behavior, and knowledge of their medical provider(25-27). Important factors include the amount of time providers spend discussing vaccinations, their communication styles, and the information available to providers(28-31). As an example of medical providers' influence on vaccination decisions, one study in the US found that primary care providers with many parents who had vaccine exemptions for their children were more likely to have concerns about vaccine safety and were less likely to perceive individual and community benefits of vaccinations than primary care providers treating fewer children with vaccine exemptions(25). A qualitative study into vaccination decision-making in the US highlighted the importance of trust between healthcare professionals and parents (32). Finally, the way that providers broach vaccination during consultations can have a determinative role in vaccination uptake, with one study recommending further investigation of

For peer review only

For peer review only - http://bmjopen.bmj.com/site/about/guidelines.xhtml

participatory (i.e. “What do you think about doing shots today?”) vs. presumptive (i.e. “Today, we are going to do some shots.”) approaches during patient-provider interactions(26).

The Swiss context

In Switzerland, childhood vaccinations are usually given by private practitioners, predominantly pediatricians and general internists. There is no national mandate for vaccination in non-epidemic settings in Switzerland, and no national vaccination registry. Despite this, vaccination coverage in Switzerland is high overall(33). The FOPH makes vaccination recommendations and distinguishes between basic[i] and complementary vaccinations(34). Both basic and complementary vaccines are reimbursed by basic mandatory health insurance when the official vaccination schedule is respected.

Given the absence of a national vaccination registry, the FOPH measures Swiss vaccination coverage at regular intervals(35). Vaccination programs and their implementation vary between the 26 cantons(34). For example, children from the French and Italian-speaking cantons have on average higher rates of measles vaccination coverage than in German-speaking cantons(36).

Cantonal coverage levels for HPV vaccination range from 79% (Valais) to 19% (Appenzell Innerhoden) for 2 doses of HPV vaccine. HPV coverage estimates only include 16-year-old girls, and estimates for boys are not yet available(37). The most common reasons for females not being vaccinated against HPV, according to a 2014 survey, included being too old, lack of information, fear of side effects, being against vaccination in general or against HPV vaccination only, HPV vaccination felt to be unnecessary, and simple logistical issues(38), with 7% and 6% of women not

For peer review only - http://bmjopen.bmj.com/site/about/guidelines.xhtml

vaccinated against HPV reporting that the physician or friends/family recommended against HPV vaccine, respectively. Further research has shown that living in cantons with school-based vaccination increases uptake of HPV vaccination (39, 40). However, spatial variation modeling has shown that variables such as political opinion, religion, and community opinion might be additional determinative factors in the presence of school-based vaccination programs(40). This suggests further research is needed to better understand vaccine hesitancy and school-based vaccination programs in Switzerland.

Vaccine hesitancy and under-immunization seem to be specific to certain population subgroups(34, 41, 42). For example, in Switzerland, measles cases, small epidemics(43), and under-immunization cluster around children attending anthroposophic (i.e. Rudolf Steiner, Waldorf) schools and around certain providers of CAM(41). One cross-sectional survey (N=1007) was administered for pediatric patients in an urban pediatric emergency department in German-speaking Switzerland. Researchers found that those who did not fully accept basic vaccinations were more frequently CAM-users than non-users, which researchers point to as reflecting parent wishes rather than physicians' recommendations(44). This relationship merits further investigation and discussion.

Studies in Switzerland show high rates of CAM use and favorable opinions, with 25% of Swiss survey respondents aged 15 and older of the 2007 Swiss Health Survey stating that they had used CAM in the previous year (45, 46). Other researchers assessed surveys conducted among the general population, doctors, hospitalized patients, and obstetric institutions and found that approximately 50% of the Swiss population had used CAM and that 85% of the population would like the cost of CAM to be covered by basic health insurance (47). CAM providers in

For peer review only

For peer review only - http://bmjopen.bmj.com/site/about/guidelines.xhtml

Switzerland are often physicians trained in conventional medicine who have then obtained additional CAM training(48), and basic mandatory health insurance reimburses four CAM services when provided by licensed physicians: (1) anthroposophic medicine, (2) Traditional Chinese Medicine (TCM) and acupuncture, (3) homeopathy, and (4) phytotherapy(49).

Rationale for the research project

The scientific literature points to existing research gaps, such as a lack of detailed information on vaccine hesitancy, patient-provider communication in Switzerland, vaccination information sources, and relations to the high prevalence of CAM use in Switzerland. This highlights the interest in an exploratory mixed method methods study investigating the vaccination perspectives and practices of patients and both CAM and biomedical providers. Our research focuses specifically on childhood vaccinations recommended in Switzerland (50) and on the vaccination against HPV, which is included in the Swiss FOPH recommendations for all adolescents 11-14 years of age. However, vaccination against HPV is recommended as a routine vaccination for females and as an complementary vaccination for males(50).

Objectives and aims

1) to explore vaccine hesitancy and provide a detailed characterization of its health system, patient-provider interaction, health communication, information sources, decision-making process, demographic, geographical and sociocultural correlates in Switzerland
2) to assess the socio-cultural and health system determinants of vaccine hesitancy and under-immunization with childhood and HPV vaccines in

For peer review only

Switzerland. This objective additionally aims at establishing the mediating effects of vaccine hesitancy on under-immunization.

3) to use the knowledge gained as the necessary background to design and submit a pilot intervention and to design tailored interventions to address vaccine hesitancy in Switzerland

Methods and design

Our research takes place in the setting of an ongoing Swiss national research program (NRP 74). The study protocol underwent successful peer review and is entirely funded by the Swiss National Science Foundation for 4 years (2017-2021). For further information, refer to: http://www.nfp74.ch/en/projects/out-patient-care/project-tarr. It represents a transdisciplinary (51)[ii] collaboration that views vaccine hesitancy as a complex, multi-faceted phenomenon. The study team is composed of researchers and medical practitioners with a varied range of backgrounds and training. An infectious disease specialist and internist leads the core study team, which includes sociologists, anthropologists, and public health specialists who collaborate closely with a steering committee composed of clinicians trained in biomedicine and CAM, a researcher in anthroposophic medicine, public health experts, and policy makers.

The project applies mixed methods through the use of a *sequential exploratory design* in order to study vaccine hesitancy across Switzerland, meaning that we first begin with qualitative methods in order to inform the tools utilized for the data collection through quantitative methods (52). An overview of the study design is shown in *Figure 1*. We recruit participants from the 3 major language regions of

For peer review only

For peer review only - http://bmjopen.bmj.com/site/about/guidelines.xhtml

Switzerland (German-, French-, and Italian-speaking) in order to examine vaccination decision-making throughout the country.

The study was approved by the local ethics committee (Ethikkommission Nordwest- und Zentralschweiz, EKNZ; project ID number 2017-00725), and ethics approval covers all study participants, including adolescent and adult patients, as well as biomedical and CAM providers. All study participants receive information about the study and provide written informed consent.

The qualitative and quantitative phases of the project both involve recruitment in medical providers' offices and interviewers with medical providers. For this purpose, we established a network of participating providers via the FOPH's Swiss Sentinella Surveillance Network and through direct recruitment by our research team. Our research team and advisory board includes both CAM and biomedical providers, who have helped recruit their colleagues for this project, sharing recruitment documents (i.e. invitation letters and study flyers), making telephone calls, and employing snowball sampling. Ongoing provider recruitment efforts have enabled us to develop a network of over 150 CAM and biomedical providers from 21 of the 24 cantons and all three language regions in Switzerland.

For the study's qualitative component, we conduct semi-structured in-depth interviews with parents, youth, and biomedical physicians and CAM providers. We also observe vaccination consultations with parents and providers and HPV information events in schools. For the study's quantitative component, we recruit participants from the practices of the network of CAM and biomedical providers across Switzerland and perform quantitative telephone interviews with parents of children 0 to 11 years of age and youth aged 15 to 26 years. We also interview the participating providers. The collection of information about vaccine hesitancy from

For peer review only - http://bmjopen.bmj.com/site/about/guidelines.xhtml

providers and their patients allows for a comparison of their perceptions of vaccinations.

Some providers participate in both the qualitative and quantitative research components, while most participate only in the quantitative phase. Importantly, our team had already established trusting relationships with a number of CAM physicians by the time of grant submission. These contacts have been crucial in expanding our network of CAM providers in Switzerland for the quantitative study phase.

Qualitative methods

The qualitative methods of this research allow us to gain deeper insight into parent and youth vaccination decision-making and patient-provider interactions about vaccination in general practice settings. The qualitative methods additionally further our understanding about public health gaps in vaccination implementation programs in Switzerland by paying attention to how participants discuss the Swiss health system in relation to vaccination. Finally, the qualitative methods will inform the design of the quantitative study component tools.

The transdisciplinary research team drafted interview guides and medical consultation observation guides to be used as qualitative study tools. The interview guides were written based on relevant vaccine hesitancy literature and guide researchers through semi-structured interviews with participants through the use of open-ended questions. Semi-structured interviews involve researchers asking the same questions systematically to all participants but allow the option for researchers to probe further by asking additional questions about themes emerging during the interview. The medical consultation observation guides allow researchers to

For peer review only

systematically focus upon items of interest highlighted in vaccine hesitancy literature on patient-provider interactions (28-31). Both the interview guides and the observation guides were piloted for coherence and clarity.

An important concept in qualitative research to determine sample sizes is the concept of saturation, which indicates that similar results are consistently collected during data collection and adequately address the research questions at hand. When saturation has been attained in qualitative research, researchers can reasonably expect similar results if the research process were to continue(53, 54). We expect to reach data saturation with the number of qualitative face-to-face interviews and observations mentioned below, but the qualitative nature of this approach allows us flexibility in adjusting the amount of data to be collected.

Qualitative interviews with biomedical and CAM providers

We conduct in-depth semi-structured qualitative interviews with at least 15 CAM providers and at least 15 biomedical physicians in providers' offices. Interview questions are organized by theme, including: (1) contextual information about the providers (i.e. type of training, type of medicine practiced, years of clinical experience, and descriptions of the types of patients they treat), (2) questions about interactions with their patients and typical vaccination consultations (i.e. amount of time spent discussing vaccination, communication strategies, and perceptions about communication training), and (3) views and perspectives on medicine, immunity, and the body (i.e. information sources on vaccinations, perceptions about their advantages and disadvantages, "natural acquired immunity" vs. vaccine acquired immunity, public health benefits compared to individual choice considerations, and vaccination rates and public health approaches in Switzerland). The interviews are

For peer review only

audio recorded and transcribed verbatim. If participants request transcriptions, we provide them so that they may clarify anything that they discussed.

Ethnographic observations of vaccination consultations

We observe a total of 30 vaccination consultations (15 with CAM providers, 15 with biomedical providers) with 10 different providers (5 CAM, 5 biomedical). We ask willing providers to allow us to observe consultations during which they discuss vaccination with parents for the first time. Prior to consultation observations, researchers discuss how they will be conducted, with both providers and patients having the option to not be observed. In cases of reluctance to participate, potential participants are able to ask questions about the study, and researchers provide them with written information about the study. We do not observe participants who do not wish to participate in the study. To date, providers not wishing to be observed are uncommon, and no patients have refused being observed. Providers explained these rare refusals by describing how they did not wish researchers' observations to disturb the natural flow of consultations with patients, not having enough time, or not seeing enough patients with whom they discuss vaccination for the first time on a daily basis.

Researchers take ethnographic field notes about the observation in field journals in order to document what happens during the interactions. They then fill out a medical consultation observation guide, which prompts them to record information about patients and providers, the reason for the consultation, who initiated the vaccination discussion, if vaccinations were discussed in a participatory or presumptive manner, questions asked by patients, if and how patients expressed hesitancy towards vaccination, researchers' perceptions of the patients' ability to

For peer review only - http://bmjopen.bmj.com/site/about/guidelines.xhtml

understand the vaccination discussion, the vaccination decision, the amount of time spent discussing vaccination, information source materials, and the researchers' perceptions of the mood, emotions, and communication between parents and providers. After observing the medical consultations, researchers use ethnographic field notes and observation guide as the basis to write observations into a descriptive, narrative format of each observed consultation.

Qualitative interviews with parents

We conduct in-depth qualitative interviews with 30 vaccine hesitant parents and 10 parents who vaccinate according to the FOPH vaccination schedule. Interviews are conducted in their homes or in a place of their choosing, such as a coffee shop. We purposefully sample more vaccine hesitant parents, particularly those who consult CAM providers for their children, since an important study assumption is that vaccine hesitancy, under-immunization by parents and their usage of CAM are co-determined. We ask parents questions thematically about: (1) family composition and parental roles (i.e. work, childcare, child healthcare decisions); (2) children's health, healthcare, and lifestyle (i.e. types of healthcare professionals parents consult for their children, health beliefs and practices, CAM use, preventive practices, etc.); (3) immunization status of the children with the support of vaccination booklets during the interview; (4) vaccination perspectives (views on individualized vaccination calendars and views on the public health benefits or consequences of vaccination) and (5) vaccination experiences (the vaccination decision-making process, vaccination discussions with healthcare professionals and others, and perceptions of social pressure to vaccinate or not vaccinate).

For peer review only - http://bmjopen.bmj.com/site/about/guidelines.xhtml

Qualitative research focus on HPV vaccination: Youth and parents

There is limited published data on adolescent female views on HPV vaccine(38, 55) and no data on adolescent male views on HPV vaccine in Switzerland. Therefore, we will additionally conduct qualitative in-depth interviews with approximately 40 youth (approximately 25 males and 15 females) recruited through the HPV quantitative research discussed below. Interviews take place in participants' homes or in a place of their choosing, such as a coffee shop. We purposively sample more male youth since there is no literature on male perspectives on HPV in Switzerland, and HPV vaccination recommendations are more recent for males than females. If youth provide their consent, the parents of 15 youth will be interviewed separately and in family focus group discussions (FGD) together with the youth. Themes of the interviews and FGD include: (1) questions relating to family composition and parental roles; (2) youth health, healthcare, and lifestyle (i.e. types of healthcare, professionals young adults/adolescents consult, health beliefs and practices, CAM use, preventive practices, etc.); (3) HPV immunization status; (4) HPV perspectives; (5) HPV vaccination experiences and the vaccination decision-making process. This last theme focuses particularly on discussions with parents, friends, acquaintances, significant others, potential sexual partners, information sources, schools' roles in providing vaccination information, and how or if the youth ever felt any social pressure to vaccinate or not vaccinate against HPV.

Qualitative research focus on HPV vaccination: Schools and public health officials

For peer review only - http://bmjopen.bmj.com/site/about/guidelines.xhtml

We observe 10 school information activities about HPV vaccines in 3-4 cantons. We choose cantons based on convenience in terms of access and ethical clearance to conduct ethnographic observations for research purposes. Researchers attend school-based events during which the HPV vaccination is presented to youth. In classrooms, our researchers will explain to students and instructors that we are there to observe and take notes on the content of the discussions as part of a study that examines how this type of information is communicated in schools. No identifying information will be documented or included in any dissemination of research results. Observation notes will be documented in field journals and subsequently written into a descriptive narrative format.

We purposively sample an estimated 2-3 public health officials from the FOPH, 2-3 cantonal authorities, and approximately 5 school physicians for expert interviews (N=10-15) in order to gain further information about how recommendations are made, discussed, planned, and implemented from a public health perspective. Interviews focus on perceptions of the implementation of vaccination programs from public health authority perspectives and are informed by specific questions from our qualitative inquiries.

Vaccination information sources

In order to better understand how information sources affect parent, youth and provider decision-making processes, we document and analyze sources of information that they discuss during the qualitative and quantitative interviews and medical consultation observations using both thematic analysis(56) and critical discourse analysis(57-59). Since the Internet's role in vaccine-related decision

For peer review only - http://bmjopen.bmj.com/site/about/guidelines.xhtml

making is well documented in the literature (14, 15, 60-62), we are particularly interested in parents' and youth's use of and interactions with Internet sources.

Qualitative data analysis

The primary research questions guiding the conduct of the qualitative research are: 1) how do parents and youth make vaccination decisions?; 2) how do medical providers, both CAM and biomedical, consider what vaccination recommendations to make to their patients?; 3) how do medical providers discuss vaccination with parents and youth; and 4) how are vaccination implementation programs envisaged from a public health perspective?

We use the COREQ checklist (63) as guidelines for reporting items of interest in the presentation of qualitative results. Since we work with diverse sources of qualitative data, we use the Framework Method(64) and grounded theory, particularly *constructivist grounded theory* as outlined by Charmaz (65), as guiding principles for our analysis. This allows our research team to structure an analytical framework throughout qualitative data analysis with the use of *sensitizing concepts* and to code data into relevant research themes while still being open to including categories and themes that emerge through qualitative data collection and analysis. Data are coded primarily by the interviewers, with coded segments being discussed with various members of the research team.

Sensitizing concepts are used in qualitative research in order to guide researchers to selectively focus on emerging themes and categories relevant to the research questions(66); for this research, sensitizing concepts include patient-provider interactions, provider and patient interaction with information sources,

For peer review only

health beliefs and practices, and interactions with and perceptions of the Swiss health system.

With the focus on patient-provider interactions in vaccination decision-making, the choice to research these questions by interviewing parents, youth and medical providers, and observing vaccination consultations will allow us to triangulate our qualitative data in order to account for the “multiple realities” of medical consultations(67). Finally, in order to fully appreciate the complexity of how people interact with health information in order to make vaccination choices, we will conduct both thematic analysis(56) and critical discourse analysis(57-59) of any sources of information mentioned as being important or determinative during vaccination discussions. A first manuscript containing initial results of the qualitative research phase has been submitted for publication (68).

Quantitative methods

The quantitative phase of our study seeks to explore and quantify the determinants of vaccine hesitancy and under-immunization with childhood and HPV vaccines in Switzerland. To this end, we developed questionnaires targeted at the different populations included in our study.

The Questionnaires

As childhood and HPV vaccination involve different populations and are generally viewed differently by patients, we will survey two separate populations: parents of young children and youth (and their parents, if possible). In addition, we survey providers to gain a better understanding of views on vaccination in our

For peer review only - http://bmjopen.bmj.com/site/about/guidelines.xhtml

diverse network of participating providers and the relationship between provider and patient views on vaccination.

To fully explore vaccine hesitancy and under-immunization in these diverse populations, we developed 4 separate questionnaires: (1) a childhood vaccination questionnaire to be administered to parents of young children, (2) a HPV vaccination survey for youth, (3) an HPV vaccination survey for parents, and (4) a questionnaire for providers. As Switzerland is a multilingual country, we developed versions of the questionnaire in the three main national languages, German, French and Italian. In addition, given the large number of immigrants living in Switzerland, we developed an English-version of the questionnaire for those who preferred doing the interview in English.

Parent and youth surveys include questions about basic socio-demographic factors, including household composition, work and educational background, income, migration status and language knowledge. In order to maximize comparability with other surveys and to allow us to weight samples so that results are more representative, we drew these questions from two recent representative national surveys in Switzerland: the Swiss version of the European Social Survey 2014 (ESS 2014) (69) and the 2012 Enquête Suisse de la Santé (70) for these questions.

As vaccine hesitancy is often correlated with political beliefs (71) and religious beliefs (72), we also include a series of questions on political and religious beliefs and practices drawn from the ESS 2014. We include three questions on each theme. For religion, we ask about interviewee's religious affiliation, the frequency with which they attend religious services and their perceptions of their own religiosity. For political beliefs, we ask about interviewee's level of interest in politics, which political

For peer review only - http://bmjopen.bmj.com/site/about/guidelines.xhtml

party they feel close to, and where they place themselves of a left-right political scale.

In addition, all questionnaires include the 20-item Moral Foundations Questionnaire (73) based on the finding in a recent US study(74) showing that moral foundations are associated with vaccine hesitancy. These survey items were all already extensively published and available in our survey languages and did not require translation or validation.

We measure under-immunization through examination of participants' vaccination records. We measure vaccine hesitancy using the 15-item PACV (Parent Attitudes about Childhood Vaccines) survey instrument, a validated measure of vaccine hesitancy and under-immunization(75). We use the original survey instrument for the childhood vaccination questionnaire and adapted questions to be appropriate to the target population for the parent and youth versions of the HPV questionnaire. For the provider questionnaire, we adapted the version of this instrument to measure vaccine hesitancy among childcare facility directors (76).

Based on preliminary findings in the qualitative phase, we developed new questions around several themes including (1) communication about vaccination with medical providers, (2) information sources consulted in vaccination decision-making, (3) perceptions about risk and control concerning possible exposure to vaccine-preventable diseases, and (4) the parent-provider relationship. For the HPV vaccination surveys, we also developed questions specific to this vaccine and its administration in Switzerland, including questions on knowledge of HPV vaccine, sources of HPV vaccination, and perceptions of school-based vaccination programs. In addition, we ask both parents and youth a series of questions about health, including questions about health status, CAM usage, and medical care usage. The

For peer review only - http://bmjopen.bmj.com/site/about/guidelines.xhtml

provider questionnaire also asks for details about providers' practices, particularly with regards to their practice of CAM. The questions we developed that are based upon preliminary findings from the qualitative phase are listed in *Appendix 1*.

French, German and Italian versions of the PACV15 were not available, so these questions and the new questions developed for these questionnaires were translated into French, German and Italian. Thirty-three key questions, including the PACV and questions developed based on preliminary results of the qualitative portion of the study were translated using the forward and backward method. Two independent translators first translated into the target language (German, French or Italian) and then reconciled any disparities in their translations. Next, two different, independent translators who were blinded to the original questions translated them back into English.

Once developed, we pre-tested and piloted the childhood vaccination and HPV versions of our questionnaires in all languages. For the pre-test, we recruited a convenience sample of 61 individuals to test the questionnaires in all four survey languages. We pre-tested each version of the questionnaire with 2-7 people and adjusted based on feedback. We then piloted the adjusted questionnaires using our full recruitment procedure (see below), conducting a further 56 interviews with the 3 questionnaires in the 4 survey languages. Finally, we pre-tested the provider questionnaire among providers in German, French and Italian. The questionnaire was also shared with the project's advisory board and adjusted based on their feedback.

Power analyses

For peer review only

For peer review only - http://bmjopen.bmj.com/site/about/guidelines.xhtml

Childhood vaccination survey. A conservative estimate is a proportion of 15% vaccine-hesitant parents in the practices of the participating biomedical and CAM providers combined (NB: 7% un-vaccinated and 12% children with delayed vaccination were recorded in the 2012 FOPH survey) (77). The sample size calculation aims at identifying the number of participants needed to detect a significant association of specific individual risk factors with vaccine hesitancy, which is here considered as a binary outcome. We further assume that factors potentially associated with vaccine hesitancy are prevalent in 20% of the population and that the odds ratio between vaccine hesitancy and a relevant risk factor is at least 2. In this case, to detect a statistically significant association with such a risk factor at the level of 0.05 with a power of 0.8, 675 participants are needed. Due to the recruitment in selected provider practices, a certain clustering of parent's characteristics is to be expected for parents recruited in the same practice, in contrast to a simple random sample. To account for such clustering, a design effect of 2 is conservatively assumed, leading to a total sample of 1,350 parents to be interviewed. This would equally allow detecting a 10% lower proportion of vaccination among parents who are vaccine-hesitant compared to a vaccination rate of 90% in the remaining population.

HPV vaccination survey. To investigate vaccine hesitancy and associations with under-immunization in the case of HPV vaccination, a separate sample of participants is required. Similar to the first case, we might also assume 15% vaccine hesitant participants, and we further anticipate a 30% lower rate of vaccination among vaccine hesitant persons than in the entire population. With a given prevalence of HPV vaccination of 53%, this would mean that among vaccine hesitant persons only 37% would be vaccinated. With power=0.8 and alpha=0.05 a sample of

For peer review only

593 participants would be needed. To account for clustering, assuming a design effect of 2, the final sample would include 1,186 participants. However, more likely is a higher vaccine hesitancy rate of 30%, given the relative novelty of HPV vaccines and the fact that it has been recommended in boys/adolescents only for the past 3 years. With power=0.8 and alpha=0.05 a sample of 361 participants would be needed. To account for the cluster sample, assuming a design effect of 2, the final sample would include 722 participants.

Based on the above power analyses, our study populations will include 1,350 parents of children aged 0 to 11 for the childhood vaccination study and 722 young people aged 15 to 21 years (for young women) or 15 to 26 years (for young men) for the HPV vaccination study.

As mentioned, there is regional and linguistic variation regarding *vaccination uptake* in Switzerland in the setting of different cantonal levels of school vaccination programs (39, 78) and health authority support for vaccination programs (79). On the other hand, we expect the prevalence of *vaccine hesitancy* and of its determinants to be relatively homogeneous across different geographic and language regions of Switzerland. Therefore, we do not specifically take geographic and language heterogeneity into account for the outcome *vaccine hesitancy.* Moreover, conditional on this assumption being correct, the statistical power to detect an association between vaccine hesitancy and the likelihood of obtaining HPV vaccination would not be sizably smaller in the presence of geographic or language heterogeneity in HPV vaccination rates than without such heterogeneity.

Recruitment and survey populations

For peer review only

We recruit our participants through the offices of the network of CAM and biomedical providers. As *Figure 2* shows, our provider network includes more than 150 CAM and biomedical providers working in all three language-regions in Switzerland and in almost every canton. Each dot represents a practice participating in the study: green dots represent CAM practitioners and blue dots biomedical providers. To ensure confidentiality, we place dots in the correct canton but do not place them at the exact provider location. Group practices are represented by a single dot.

Providers are CAM or biomedical providers who treat children and/or youth and regularly deal with questions regarding vaccinations and vaccine decisions. They include pediatricians, general practitioners, gynecologists, CAM physicians, and CAM providers without formal medical training working independently or in group practices.

We interview parents and young people by telephone. Each telephone interview will last approximately 30-35 minutes. Parents will be recruited from urban and rural medical practices in French, German and Italian speaking areas of Switzerland. In addition, we interview each participating provider in order to examine the relationships between provider perspectives, patient perspectives, and vaccine hesitancy. For the HPV portion of our study, we will interview one of the participating youth's parents if the youth allow us to do so.

To be eligible to participate in the study, participants fulfill the following criteria. For the childhood vaccination sub-study, parents are 18 years old or older with a child aged 0 to 11. For the quantitative HPV portion of our study, young women are between the ages of 15 and 21 (born between July 1, 1997 and July 1, 2003) and young men are between the ages of 15 and 26 (born between July 1,

For peer review only - http://bmjopen.bmj.com/site/about/guidelines.xhtml

1992 and July 1, 2003). These differences are due to the timing of HPV vaccine recommendations for girls and boys. HPV vaccine reimbursement for girls 11-14 (target age) and 15-19 (catch-up age) started January 1, 2008. HPV vaccination reimbursement for males began on July 1, 2016 in Switzerland. Given the newness of this recommendation, we enroll youth males born between July 1, 1992 and July 1, 2003 in order to gather data on all males aged 15 to 26 who have been eligible for the HPV vaccine through Swiss vaccine programs. All study participants (parents, youth, and providers) must speak one of the main Swiss national languages (French, German or Italian) or English.

We recruit all parents and youth through providers in order to be able to link the data from the parents with their provider's data for a comparison by type of provider. We use a quota sampling technique as CAM providers are likely to see fewer patients per day. Our goal is to recruit a maximum of 20 parents of young children and a maximum of 15 youth per provider based on a rigorous sampling approach in a defined period. Recruitment targets for group practices are no more than three times the target for individual providers. Thus, targets in dual practices are 40 parents and 30 youth, and for practices with 3 or more providers, targets are 60 parents and 45 youth. For every practice, the total number of eligible patients in a specific time period will be established, which allows post-hoc weighting to the known total of eligible patients in this time-period.

Given the diversity of providers and differences in our study sub-populations, we employ a variety of recruitment techniques. In large practices or practices with high patient-volume, interviewers[iii] will recruit participants directly in provider's waiting rooms. For in-office recruitment, interviewers spend 2-5 days in providers' waiting rooms, with the goal of recruiting a maximum of 20 parents and/or a

For peer review only

For peer review only - http://bmjopen.bmj.com/site/about/guidelines.xhtml

maximum of 15 youth for interviews. Interviewers contact all eligible patients to present the study and ask whether they are interested in participating. For those who agree, interviewers obtain informed consent and a copy of the vaccination certificate (if possible) in waiting rooms and then arrange to conduct a telephone interview at a later time. In large group practices, interviewers have the option to work as a team to manage particularly high patient volumes.

In smaller practices or practices with lower patient volumes for the targeted groups, we ask providers to recruit participants retrospectively by reviewing their patient log from the last 2-6 months and contacting eligible patients to see if they would be interested in participating in the study. Providers transmit a detailed record of attempted contacts and take note of refusals and interested parties. We then contact those who indicate an interest by phone to present the study, obtain informed consent and a copy of the vaccination certificate (if possible) and arrange for a telephone interview. Interviewers select potential participants randomly from the lists provided until they have the required quota of participants in each group.

Interviewers determine the most appropriate recruitment technique during a first visit with the provider. As some providers may have high volumes of one population and low volumes of another (e.g., pediatricians may see many young children but few youths 15 of age or over), we may employ different recruitment methods for the different sub-samples.

Experience to date shows that providers generally accept our preferred recruitment practice for their type of practice. However, we work with providers to find workable solutions if they are uncomfortable with or unable to accommodate our standard recruitment procedures. Although small practices, particularly for CAM providers, are often hesitant to let interviewers recruit in waiting rooms, such

For peer review only - http://bmjopen.bmj.com/site/about/guidelines.xhtml

practices rarely have sufficient patient volume to merit that approach. Very few large volume providers have been unwilling to allow interviewers to recruit in waiting rooms, however, when they are, we implement the provider recruitment practice used in smaller practices. More commonly, practices do not have the capacity to review patient logs and contact patients. In that case, as approved by the local ethics commissions, interviewers can assist medical providers and their assistance in reviewing logs and contacting patients from providers' offices.

For all recruitment techniques, we track how many people were contacted, how many agreed to participate or be contacted again, how many gave consent to interviews and how many completed interviews. This information will allow us to assess the refusal rate, the drop-out rate and the number of individuals lost-to-follow up.

Additionally, we conduct a quantitative telephone or face-to-face survey with all participating providers during the quantitative component of the study, in order to quantitatively assess if providers' vaccine perspectives are associated with those of their patients.

Quantitative data analysis

Our primary quantitative research questions are;

1) What are the determinants of vaccine hesitancy for childhood and HPV vaccinations in Switzerland?

2) What is the relationship between vaccine hesitancy and under-immunization?

We measure vaccine hesitancy based on the PACV score. As this instrument has not previously been used in Switzerland or in our survey languages, we first test

For peer review only

For peer review only - http://bmjopen.bmj.com/site/about/guidelines.xhtml

its validity for Switzerland using Mokken scale analysis to confirm the unidimensionality of the scale. Individuals with a PACV score of ≥50 are considered vaccine hesitant, and individuals with a PACV score <50 are not considered vaccine hesitant (75). We will use multilevel logistic regression to assess the influence of different variables on vaccine hesitancy. We will explore associations between vaccine hesitancy and a number of individual level characteristics, including: age, sex, household composition, place of birth, immigration status, household income, employment status and work hours, daycare usage, highest achieved level of education, political affiliations, religion, CAM usage, trust in medical providers and satisfaction with consultations, sources of information about vaccination consulted, views on parents' role in vaccine decisions, views of importance of health, views of risks of vaccine-preventable diseases, and moral foundations. In addition, our research design allows us to explore how factors at higher levels are associated with vaccine hesitancy, including how vaccine hesitancy varies by canton and language region and by provider or by various provider characteristics (CAM vs. biomedical providers), and to what extent vaccine hesitancy of the parent/youth correlates with vaccine hesitancy of the provider. Finally, although our data is not representative, we can weight results to allow us to roughly estimate the prevalence of vaccine hesitancy in Switzerland.

Study Status

This study is ongoing, with some parts of the research completed, others ongoing, and others yet to begin. For the qualitative portion of the study, we have completed qualitative interviews with biomedical and CAM providers and ethnographic observations of vaccination consultations. We have also completed qualitative

For peer review only

interviews with parents of young children. Qualitative interviews with youth and parents focusing on HPV vaccination are ongoing. We are at the site and interview recruitment stage for our qualitative research on HPV vaccination at schools and with public health officials. Finally, we are compiling a list of vaccination information sources based on responses to questions related to this subject in the quantitative questionnaires and discussions in qualitative interviews with parents and youth. We will begin analysis once this list in complete, at the end of data collection for qualitative interviews and the questionnaires. For the quantitative portion of our study, we have completed questionnaire design and testing and the power analysis to determine sample size. We are currently recruiting and interviewing parents, youth and providers for the survey, with approximately half of interviews completed. We continue to recruit providers as well.

Discussion

By using a sequential exploratory mixed methods study design(52), this study will provide rich and multi-faceted data on vaccination decision-making and vaccine hesitancy in Switzerland. In line with such a study design, the qualitative data informed the tools used for the quantitative data collection component. The results of both qualitative and quantitative components of our study will likely be complementary to one another and will allow us to answer different types of research questions. That said, any discrepancies or contradictory findings between the qualitative and quantitative components will merit further investigation, which we will highlight in dissemination of study results. Additionally, given the multi-faceted nature of vaccine hesitancy and health decision-making and the study's exploratory mixed methods study design, the results from both qualitative and quantitative components

For peer review only

For peer review only - http://bmjopen.bmj.com/site/about/guidelines.xhtml

will provide us with rich and detailed data for the Swiss context, where there is limited available data.

A particular strength of this study is its focus on perspectives of both medical providers and their patients. Studies have shown that providers remain a trusted source of information for parents and youth facing vaccine decisions, so a greater understanding of provider perspectives is important(25-32). Another major strength of this study is its inclusion of the perspectives of both CAM and biomedical providers. While a growing literature explores the association between vaccine-hesitancy and CAM usage (6, 12, 19, 20), few studies include the perspective of CAM providers and none that we know of has the extent of cooperation with CAM providers found here.

Qualitative interviews provide key insights into parents, youths and CAM and biomedical providers' perspectives on vaccination. In addition, interviews with CAM and biomedical providers shed light onto their views on vaccination, vaccine hesitant patients, and how they interact with this population in their practices. Observations of vaccine consultations in offices provide another opportunity to get a sense of the variety of approaches providers employ when broaching the subject of vaccination. The combination of qualitative interviews with numerous stakeholders in vaccine decision-making and planning, ethnographic observations of vaccination consultations and school information sessions about the HPV vaccination, and qualitative analysis of vaccination information sources allow for qualitative triangulation into the multifaceted phenomenon of vaccine hesitancy in Switzerland. Such a triangulation of qualitative data through multiple qualitative methods allows us to better understand the 'multiple realities' of medical consultations (67), in

For peer review only

addition to allowing us to explore how various actors interact with and perceive the Swiss health system in regards to vaccination.

Although data are not strictly representative of Switzerland, data from the quantitative survey will give us a sense of the overall prevalence of vaccine hesitancy in Switzerland and how this varies between language regions, cantons, and urban, suburban, and rural settings. More importantly, it allows us to explore the association of vaccine hesitancy with other factors, like CAM-usage, religion and spirituality, moral foundations, and political orientation. In addition, by pairing data from providers and their patients, we can see whether there is an association between providers' and patients' views on vaccines. The quantitative data will also give insight into the major sources of information parents and youth draw on when making vaccination decisions. Finally, the mixed methods study design allowing for the qualitative component to inform the quantitative component also allows us to triangulate both the qualitative and quantitative data gathered throughout the duration of the study so that results from both components can be compared.

Limitations

There are several limitations to the various parts of our study. A major limitation, which we also argue should be seen as a study strength, comes from our focus on recruiting participants through providers. This technique has major advantages when seeking to examine effects of provider characteristics on patient beliefs and behaviors, particularly for a topic such as vaccine hesitancy where trust between patients and providers has been shown to be a major determinant(32). We also included items in both qualitative interview guides and quantitative questionnaires in order to gauge to what extent patients select physicians they trust

For peer review only

For peer review only - http://bmjopen.bmj.com/site/about/guidelines.xhtml

or whose practices or attitudes align with their own, a patient practice which has been documented in vaccine hesitancy literature (80). These questions also allow us to assess if disagreements between patients and providers have brought patients to seek care elsewhere in more provider-driven selection processes.

The quantitative component recruitment strategy indicates that populations that do not regularly see medical providers are under-represented in our data. While universal health insurance requirements attempt to ensure that most people living in Switzerland have access to health care, certain populations are still under-represented, including recent migrants and undocumented populations. Recruiting through providers poses further questions of selection bias, as people who see doctors more often are more likely to participate in our study. This bias means that, for example, parents of infants were more apt to be recruited than parents of older children, as infants see the doctor more regularly. Selection bias problems are particularly relevant to youth populations, as most youth are healthy and thus among the least likely to regularly seek medical care. As such, youth in our study may differ in important ways from youth in Switzerland overall. In addition, as our provider network was established through personal recruitment by members of the study team and snowball sampling, the network itself is not representative and subject to selection bias. In our view, the advantages of being able to link provider and patient data outweighed these limitations.

For qualitative interviewing, interviewer effects may affect the type and quality of information we received; interviews and observations are conducted by a male medical sociologist trained in qualitative research methods, a female medical doctor trained in qualitative research methods, a female senior medical student with qualitative research training, and a female medical anthropologist trained in

For peer review only

qualitative research methods. That being said, having a variety of researchers with different backgrounds involved in the data collection and analytical process adds to the richness of the analysis through clarification of concepts during in-depth research team discussions. Another limitation arises from the way we observed vaccine discussions. Observers watch the discussion in person and take notes, but do not audio or video record discussions. The absence of such recordings limits our ability to rigorously analyze the conversations, while the physical presence of observers in the consultation may have had an effect on discussions. Furthermore, given that the data are collected in both French-speaking and German-speaking regions of Switzerland, we have a multilingual corpus which will necessitate in-depth discussions between study members during qualitative data analysis.

Furthermore, as noted above, the quantitative study will not be based on a representative sample but will instead allow comparing results between different types of providers and their patients, and between different socio-cultural regions of Switzerland. As in the qualitative component, interviewer effects may affect the type and quality of information we received. We will include data quality controls into data analysis in order to check for interviewer effects. Additionally, linguistic barriers, particularly with recent migrants to Switzerland, may prove challenging for participants to provide accurate responses to our interview questions.

Scientific relevance

Vaccine hesitancy is a complex public health issue in high-income countries, yet there is no agreed upon measure of vaccine hesitancy. Multilingual, validated instruments for measuring vaccine hesitancy are rare, making comparative study difficult. This study develops and test translations of the 15-item PACV in three new

For peer review only - http://bmjopen.bmj.com/site/about/guidelines.xhtml

languages and provides the valuable assessments of vaccine hesitancy in French-, German- and Italian-speaking contexts. Furthermore, our study design includes vaccination certificates in order to statistically examine the relationship between vaccine hesitancy and under-immunization.

Results from the study will also make a contribution to the literature on the relationship between CAM-usage and vaccine hesitancy. The combination of rich qualitative data including both biomedical and CAM perspectives and provider-linked data from patients who see CAM-practitioners will allow a more in-depth examination of this relationship than found in the literature to date.

A combination of the data collected through the quantitative and qualitative methods and their analysis will form the necessary background for the design, submission, and implementation of an appropriate intervention randomized control trial intervention designed at improving vaccine communication and counseling among physicians, parents, and adolescents in Switzerland.

Patient and public involvement

Study participants, both patients and medical providers, are involved in the study design throughout the duration of the data collection. Study tools are piloted and tested by the research team who requests patient and provider input during this process. Participants are given the opportunity to indicate any additional comments throughout data collection so that we may take their priorities, experiences, and preferences into account. Patients (parents and adolescents) and providers assist us in additional recruitment efforts as described in the methods section. All participants were informed of the amount of time their participation would entail prior to data collection. We systematically request participants' wishes to receive the results of the study by including an item on the consent form allowing them to provide us with their

For peer review only - http://bmjopen.bmj.com/site/about/guidelines.xhtml

894 contact information. We will send relevant published results to study participants and
895 invite them to public forums where we discuss our study results.

896 **Ethics and dissemination**

897 The study is conducted in compliance with this study protocol, the Swiss
898 Federal Act on Research Involving Human Beings (Human research Act, HRA), and
899 the Declaration of Helsinki. The study was approved by the local ethics committee
900 (Ethikkommission Nordwest- und Zentralschweiz, EKNZ; project ID number 2017-
901 00725); ethics approval covers all study participants, including adolescent and adult
902 patients, as well as biomedical and CAM providers. All study participants receive
903 information about the study and provide written informed consent. Importantly, and
904 as approved by the ethics committee, all questionnaire items include “do not wish to
905 answer” categories, given the personal and sensitive nature of certain questions, in
906 particular those regarding religion and political views. In accordance with the Human
907 Research Act, adolescents (defined as individuals 14 years of age or older) who are
908 capable of judgement are able to provide informed consent in writing and do not
909 need to provide informed consent in writing from a legal representative “if the
910 research project entails more than minimal risks and burdens” (HRA, Art. 23, Par. 1).
911 The EKNZ has approved of this study as entailing less than minimal risks and
912 burdens and thus does not require informed consent in writing from a legal
913 representative for adolescents. Informed consent from minor participants (all aged
914 15 years and older for this study) is obtained directly from the participants and not
915 their legal representatives.

916 Study results will be published in peer-reviewed academic journals and
917 disseminated to healthcare professionals, researchers, and the public via academic

For peer review only

conferences and public presentations. The datasets used and/or analyzed during the current study are available from the corresponding author on reasonable request.

References

1. Centers for Disease C, Prevention. Ten great public health achievements-- United States, 1900-1999. MMWR Morb Mortal Wkly Rep. 1999;48(12):241-3.

2. Maglione MA, Das L, Raaen L, Smith A, Chari R, Newberry S, et al. Safety of Vaccines Used for Routine Immunization of US Children: A Systematic Review. Pediatrics. 2014.

3. Thompson KM, Lewis Odahowski C. Systematic Review of Health Economic Analyses of Measles and Rubella Immunization Interventions. Risk Analysis. 2014;36(7):1297-314. doi: 10.111/risa.12331.

4. Larson HJ, Jarrett C, Eckersberger E, Smith DMD, Paterson P. Understanding vaccine hesitancy around vaccines and vaccination from a global perspective: A systematic review of published literature, 2007–2012. Vaccine. 2014;32(19):2150-9.

5. Omer SB, Salmon DA, Orenstein WA, deHart MP, Halsey N. Vaccine Refusal, Mandatory Immunization, and the Risks of Vaccine-Preventable Diseases. N Engl J Med. 2009;360(19):1981-8.

6. Peretti-Watel P, Larson HJ, Ward JK, Schulz WS, Verger P. Vaccine Hesitancy: Clarifying a Theoretical Framework for an Ambiguous Notion. PLoS Currents. 2015;7:ecurrents.outbreaks.6844c80ff9f5b273f34c91f71b7fc289.

7. Eskola J, Duclos P, Schuster M, MacDonald NE. How to deal with vaccine hesitancy? Vaccine. 2015;33(34):4215-7.

8. Larson HJ, Jarrett C, Schulz WS, Chaudhuri M, Zhou Y, Dube E, et al. Measuring vaccine hesitancy: The development of a survey tool. Vaccine. 2015;33(34):4165-75.

9. World Health O. WHO | Vaccine hesitancy: A growing challenge for immunization programmes. WHO. 2015.

10. WHO. Report of the SAGE Working Group on Vaccine Hesitancy. 2014.

11. WHO. Ten threats to global health in 2019 2019 [Available from: https://www.who.int/emergencies/ten-threats-to-global-health-in-2019.

12. Poland CM, Brunson EK. The need for a multi-disciplinary perspective on vaccine hesitancy and acceptance. Vaccine. 2015;33(2):277-9.

13. Brunson EK. The Impact of Social Networks on Parents' Vaccination Decisions. Pediatrics. 2013;131(5):e1397-404.

14. Betsch C, Sachse K. Dr. Jekyll or Mr. Hyde? (How) the Internet influences vaccination decisions: Recent evidence and tentative guidelines for online vaccine communication. Vaccine. 2012;30(25):3723-6.

15. Kata A. A Postmodern Pandora's Box: Anti-Vaccination Misinformation on the Internet. Vaccine. 2010;28(7):1709-16.

16. Yaqub O, Castle-Clarke S, Sevdalis N, Chataway J. Attitudes to vaccination: A critical review. Social Science & Medicine. 2014;112:1-11.

For peer review only

For peer review only - http://bmjopen.bmj.com/site/about/guidelines.xhtml

17. Wang E, Baras Y, Buttenheim AM. “Everybody just wants to do what's best for their child”: Understanding how pro-vaccine parents can support a culture of vaccine hesitancy. Vaccine. 2015;33(48):6703-9.

18. Sobo EJ, Huhn A, Sannwald A, Thurman L. Information Curation among Vaccine Cautious Parents: Web 2.0, Pinterest Thinking, and Pediatric Vaccination Choice. Medical Anthropology. 2016;35(6):529-46.

19. Browne M, Thomson P, Rockloff MJ, Pennycook G. Going against the Herd: Psychological and Cultural Factors Underlying the ‘Vaccination Confidence Gap’. PLOS ONE. 2015;10(9):e0132562.

20. Wardle J, Frawley J, Steel A, Sullivan E. Complementary medicine and childhood immunisation: A critical review. Vaccine. 2016;34(38):4484-500.

21. Gray JM. Postmodern medicine. 1999;354(9189):1550-3.

22. Beck U, Ritter M. Risk society: towards a new modernity. London: Sage Publications; 1992.

23. Greenhalgh T, Wessely S. ‘Health for me’: a sociocultural analysis of healthism in the middle classes. British Medical Bulletin. 2004;69(1):197-213.

24. Armstrong D. Actors, patients and agency: a recent history. Sociology of Health & Illness. 2014;36(2):163-74.

25. Salmon DA, Pan WKY, Omer SB, Navar AM, Orenstein W, Marcuse EK, et al. Vaccine knowledge and practices of primary care providers of exempt vs. vaccinated children. Human Vaccines. 2008;4(4):286-91.

26. Opel DJ, Heritage J, Taylor JA, Mangione-Smith R, Salas HS, DeVere V, et al. The Architecture of Provider-Parent Vaccine Discussions at Health Supervision Visits. Pediatrics. 2013;132(6):1037.

27. Verger P, Fressard L, Collange F, Gautier A, Jestin C, Launay O, et al. Vaccine Hesitancy Among General Practitioners and Its Determinants During Controversies: A National Cross-sectional Survey in France. EBioMedicine. 2015;2(8):891-7.

28. Kimmel SR, Burns IT, Zimmerman RK. Addressing immunization barriers, benefits, and risks. 2003;52(1 Suppl):S47.

29. Bryant KA, Wesley GC, Wood JA, Hines C, Marshall GS. Use of standardized patients to examine physicians’ communication strategies when addressing vaccine refusal: A pilot study. Vaccine. 2009;27(27):3616-9.

30. Opel DJ, Robinson JD, Heritage J, Korfiatis C, Taylor JA, Mangione-Smith R. Characterizing providers’ immunization communication practices during health supervision visits with vaccine-hesitant parents: A pilot study. Vaccine. 2012;30(7):1269-75.

31. Paterson P, Meurice F, Stanberry LR, Glismann S, Rosenthal SL, Larson HJ. Vaccine hesitancy and healthcare providers. Vaccine. 2016;34(52):6700-6.

32. Benin AL, Wisler-Scher DJ, Colson E, Shapiro ED, Holmboe ES. Qualitative analysis of mothers' decision-making about vaccines for infants: the importance of trust. American Academy of Pediatrics. 2006;117(5):1532-41.

33. FOPH. Durchimpfung von 2-, 8- und 16-Jährigen in der Schweiz, 2011 bis 2013. 2015(28):538-43.

34. Masserey Spicher V. The Federal Vaccination Commission in Switzerland: An officially appointed independent commission ensuring evidence-based recommendations and transparent procedures. Vaccine. 2010;28:A48-A53.

35. Bundesamt für Gesundheit BO. Durchimpfung von 2-, 8- und 16-Jährigen in der Schweiz, 2011 bis 2013. BAG Bulletin; 2015.

For peer review only

For peer review only - http://bmjopen.bmj.com/site/about/guidelines.xhtml

36. Lang P, Zimmermann H, Piller U, Steffen R, Hatz C. The Swiss National Vaccination Coverage Survey, 2005-2007. Public Health Reports (1974-). 2011;126:97-108.

37. FOPH. Couverture vaccinale des enfants âgés de 2, 8 et 16 ans en Suisse, 2014–2016. FOPH Bulletin: FOPH; 2018. Contract No.: 24.

38. Bundesamt für Gesundheit BO. Die HPV-Impfung in der Schweiz: Resultate einer nationalen Befragung im Jahr 2014. BAG Bulletin. 2015(23):445-52.

39. Riesen M, Garcia V, Low N, Althaus CL. Modeling the consequences of regional heterogeneity in human papillomavirus (HPV) vaccination uptake on transmission in Switzerland. bioRxiv. 2017:125518.

40. Riesen M, Konstantinoudis G, Lang P, Low N, Hatz C, Maeusezahl M, et al. Exploring variation in human papillomavirus vaccination uptake in Switzerland: a multilevel spatial analysis of a national vaccination coverage survey. BMJ Open. 2018;8(5):e021006.

41. Richard JL, Masserey Spicher V. Large measles epidemic in Switzerland from 2006 to 2009: consequences for the elimination of measles in Europe. Euro Surveillance. 2009;14(50):pii-19443.

42. Altpeter E, Wymann MN, Richard J-L, Mäusezahl-Feuz M. Marked increase in measles vaccination coverage among young adults in Switzerland: a campaign or cohort effect? International Journal of Public Health. 2018;63(5):589-99.

43. Bundesamt für Gesundheit BO. Neue Welle der Masernepidemie Anfang 2009: Beschreibung und Massnahmen. BAG Bulletin. 2009(27):484-91.

44. Zuzak TJ. Attitudes towards vaccination: users of complementary and alternative medicine versus non-users. Swiss Med Wkly. 2008;138.

45. Klein SD, Torchetti L, Frei-Erb M, Wolf U. Usage of Complementary Medicine in Switzerland: Results of the Swiss Health Survey 2012 and Development Since 2007. PLOS ONE. 2015;10(10):e0141985.

46. Simões-Wüst AP, Rist L, Dettling M. Self-Reported Health Characteristics and Medication Consumption by CAM Users and Nonusers: A Swiss Cross-Sectional Survey. Journal of Alternative and Complementary Medicine. 2014;20(1):40-7.

47. Wolf U, Maxion-Bergemann S, Bornhöft G, Matthiessen P, Wolf M. Use of Complementary Medicine in Switzerland. Complementary Medicine Research. 2006;13(suppl 2)(s02):4-6.

48. Hart J. Complementary and Alternative Medicine Use and Initiatives in Europe. Alternative and Complementary Therapies. 2017;24(1):32-4.

49. FOPH SFOoPH. RS 818.101 Loi fédérale du 28 septembre 2012 sur la lutte contre les maladies transmissibles de l'homme (Loi sur les épidémies, LEp) 2018 [Available from: https://www.admin.ch/opc/fr/classified-compilation/20071012/index.html.

50. FOPH. Plan de vaccination suisse 2019 [Available from: https://www.bag.admin.ch/bag/fr/home/gesund-leben/gesundheitsfoerderung-und-praevention/impfungen-prophylaxe/schweizerischer-impfplan.html.

51. Rosenfield PL. The potential of transdisciplinary research for sustaining and extending linkages between the health and social sciences. Social Science & Medicine. 1992;35(11):1343-57.

52. Creswell JW, Plano Clark VL. Designing and Conducting Mixed Methods Research. 2 ed. Los Angeles: SAGE; 2011. 443 p.

53. Morse JM. The Significance of Saturation. Qualitative Health Research. 1995;5(2):147-9.

For peer review only - http://bmjopen.bmj.com/site/about/guidelines.xhtml

54. Nelson J. Using conceptual depth criteria: addressing the challenge of reaching saturation in qualitative research. Qualitative Research. 2017;17(5):554-70.

55. Y B-D, C A, A B, D M, B L, Z T, et al. Human Papillomavirus (HPV) Vaccine in Switzerland: The reasons why some young women do not get vaccinated. Joint Annual Meeting of the Swiss Society of Paediatrics, Swiss Society of Paediatric Surgery and Swiss Society of Child and Adolescent Psychiatry and Psychotherapy; May 24-25, 2018; Lausanne, Switzerland2018.

56. Braun V, Clarke V. Using thematic analysis in psychology. Qualitative Research in Psychology. 2006;3(2):77-101.

57. Fairclough N. Critical Discourse Analysis: The Critical Study of Language. London: Longman; 1995 1995. 304 p.

58. Van Dijk TA. Principles of critical discourse analysis. 1993;4(2):249-83.

59. van Dijk TA. Critical Discourse Analysis. The Handbook of Discourse Analysis: Blackwell Publishers Ltd; 2008. p. 349-71.

60. Stahl JP, Cohen R, Denis F, Gaudelus J, Martinot A, Lery T, et al. The impact of the web and social networks on vaccination. New challenges and opportunities offered to fight against vaccine hesitancy. Médecine et Maladies Infectieuses. 2016;46(3):117-22.

61. Witteman HO, Zikmund-Fisher BJ. The defining characteristics of Web 2.0 and their potential influence in the online vaccination debate. Vaccine. 2012;30(25):3734-40.

62. Betsch C, Brewer NT, Brocard P, Davies P, Gaissmaier W, Haase N, et al. Opportunities and challenges of Web 2.0 for vaccination decisions. Vaccine. 2012;30(25):3727-33.

63. Tong A, Sainsbury P, Craig J. Consolidated criteria for reporting qualitative research (COREQ): a 32-item checklist for interviews and focus groups. International Journal for Quality in Health Care. 2007;19(6):349-57.

64. Gale N, Heath G, Cameron E, Rashid S, Redwood S. Using the framework method for the analysis of qualitative data in multi-disciplinary health research. BMC Medical Research Methodology. 2013;13(1):117.

65. Charmaz K. Constructing Grounded Theory: A Practical Guide through Qualitative Analysis. 1st ed. London, Thousand Oaks, New Delhi: SAGE Publications; 2006. 208 p.

66. Bowen GA. Grounded Theory and Sensitizing Concepts. International Journal of Qualitative Methods. 2006;5(3):12-23.

67. Barry CA. Multiple Realities in a Study of Medical Consultations. Qualitative Health Research. 2002;12(8):1093-111.

68. Deml M, Notter J, Kliem P, Buhl A, Huber BM, Pfeiffer C, et al. “We treat humans, not herds!”: A qualitative study of complementary and alternative medicine (CAM) providers’ individualized approaches to vaccination in Switzerland. Social Science & Medicine. Under Review.

69. (ESS) ESS. ESS 2014 Questionnaire Switzerland. 2014. p. 1-93.

70. (OFS) SFSO. Enquête suisse sur la santé 2012. Neuchâtel, Switzerland2014. p. 1-115.

71. Dubé E, Gagnon D, Nickels E, Jeram S, Schuster M. Mapping vaccine hesitancy--country-specific characteristics of a global phenomenon. Vaccine. 2014;32(49):6649-54.

72. Marti M, de Cola M, MacDonald NE, Dumolard L, Duclos P. Assessments of global drivers of vaccine hesitancy in 2014—Looking beyond safety concerns. PLOS ONE. 2017;12(3):e0172310.

73. Graham J, Nosek BA, Haidt J, Iyer R, Koleva S, Ditto PH. Mapping the moral domain. J Pers Soc Psychol. 2011;101(2):366-85.

For peer review only

For peer review only - http://bmjopen.bmj.com/site/about/guidelines.xhtml

74. Amin AB, Bednarczyk RA, Ray CE, Melchiori KJ, Graham J, Huntsinger JR, et al. Association of moral values with vaccine hesitancy. Nature Human Behaviour. 2017;1(12):873-80.

75. Opel DJ, Taylor JA, Zhou C, Catz S, Myaing M, Mangione-Smith R. The relationship between parent attitudes about childhood vaccines survey scores and future child immunization status: A validation study. JAMA Pediatrics. 2013;167(11):1065-71.

76. Opel DJ, Banerjee A, King P, Paul C, Glassy D, Yasuda K. Washington State Licensed Child Care Facility Directors' Perspectives on Childhood Immunization. Journal of the Pediatric Infectious Diseases Society. 2012;2(1):40-9.

77. Bundesamt für Gesundheit BO. Masernimpfung in der Schweiz im 2012: Resultate einer nationalen Bevölkerungsbefragung zum Thema Masern. BAG Bulletin. 2013(17):278-83.

78. Lang P, Sinniger P, Spaar A, Born R, Hatz C. Evolution of the HPV vaccination coverage in Switzerland, 2008-2016. Swiss Public Health Conference 2017; November 22-23, 2017; Basel, Switzerland2017.

79. Masserey Spicher V. Facteurs expliquant les différences de couverture vaccinale entre cantons en Suisse : résultats de l’étude FEVAC réalisée en 2014–2015. 2018(9):12-21.

80. Peretti-Watel P, Ward JK, Vergelys C, Bocquier A, Raude J, Verger P. ‘I Think I Made The Right Decision … I Hope I'm Not Wrong’. Vaccine hesitancy, commitment and trust among parents of young children. Sociology of Health & Illness. 2019;41(6):1192-206.

For peer review only

Figure Legends

Figure 1: Study overview

Figure 2: Network of participating providers

Author Statement

Authors’ contributions

MD co-drafted the manuscript and focused on the qualitative components. KJ co-drafted the manuscript and focused on the quantitative components. SM initiated the drafting of the manuscript and provided valuable feedback during its writing. BH assisted in establishing a network of complementary and alternative medical (CAM) practitioners and provided expertise in both pediatrics and CAM in the Swiss context. AB participated in developing the methods for the qualitative research specific to HPV. EF, VM, JS, PK, and RC coordinated quantitative data collection efforts and were instrumental in the preparation of the study’s quantitative tools and data

For peer review only - http://bmjopen.bmj.com/site/about/guidelines.xhtml

management. DK participated regularly in study advisory board meetings and provided insight into CAM perspectives and feedback about the study design. CP took a lead role in establishing the study's qualitative methodologies. CBJ provided regular study supervision and inputs about the qualitative results. PET is the principal investigator, directed the funding request, and supervised the conduct of the study in its entirety. He provided infectious disease and general medical expertise, oversaw study conception, design, data collection, analysis, and interpretation. All authors read, contributed to, and approved the final manuscript.

Funding statement

The study is funded in its entirety via the Swiss National Science Foundation, in the setting of National Research Program (NRP) 74. No funding was obtained from vaccine manufacturers or the Federal Office of Public Health.

Competing interests statement

Our research team attests that there is no conflict of interest and that our study will be conducted independently of outside influences in terms of specific intellectual, financial, and proprietary agendas.

Data Statement

The datasets used and/or analyzed during the current study are available from the corresponding author on reasonable request.

Abbreviations

For peer review only

CAM: Complementary and alternative medicine; FGD: Focus group discussion; EKNZ: Ethikkommission Nordwest- und Zentralschweiz; FOPH: Swiss Federal Office of Public Health; HPV: Human papillomavirus; Human Research Act: HRA; PACV: Parent Attitudes about Child Vaccine; SAGE: Strategic Advisory Group of Experts; TCM: Traditional Chinese Medicine; WHO: World Health Organization

Acknowledgments

The authors would like to thank Christoph Berger (University Children's Hospital Zurich), Andreas Zeller (University Institute of Primary Care Medicine Basel), Mirjam Mäusezahl (Epidemiology Section, Federal Office of Public Health, Bern), Julia Notter (Medizinische Universitätsklinik, Kantonsspital Baselland, University of Basel, Switzerland), and Christian Schindler (Senior Statistician, Swiss Tropical and Public Health Institute, University of Basel, Switzerland) for their important contributions to protocol development and for many helpful discussions. Some of the study's methods and preliminary findings have previously been presented at the conferences listed below:

- Notter J., Deml M, Huber B, Krüerke D, Jafflin K, Zeller A, Mäusezahl M, Berger C, Merten S, Burton-Jeangros C, Pfeiffer C, Tarr PE. Poster presentation at SSI (Swiss Society for Infectious Diseases) Joint Annual Meeting in Interlaken, 14 September 2018; "Complementary and alternative medical (CAM) providers' patient-centered, individualized approaches to vaccination in Switzerland"
- Tarr P. Oral presentation at Global challenges in vaccine acceptance science and programs, 24 - 26 September 2018 - Les Pensières Center for Global

For peer review only

Health, Veyrier du Lac, France; "Approaches of complementary and biomedical providers to vaccinations in Switzerland"

- Deml M. Oral presentation at Swiss Public Health Conference in Neuchâtel, 8 November 2018; "Vaccination Policy, Vaccine Uptake, and Vaccine Hesitancy in Switzerland"

Word count: 11,748

---

[i] *Basic* vaccines are defined as "essential to individual and public health, and offer a level of protection that is indispensable to people's well-being (e.g., diphtheria, tetanus, pertussis, polio, MMR, HBV, HPV)." (p. A51) *Complementary vaccines* are defined as being able to "maximize individual protection and are meant for individuals seeking protection from well-defined risks (e.g., conjugate pneumococcal vaccine and conjugate meningococcus C vaccine)." (p. A51)

[ii] Rosenfield (1992) explains how *transdisciplinary research* can be conducted, particularly in health research: "Representatives of different disciplines are encouraged to transcend their separate conceptual, theoretical, and methodological orientations in order to develop a shared approach to the research, building on a common conceptual framework. Such a framework can be used to define and analyze the research problem and develop new approaches for health care that more closely represent the historical and present-day reality in which health problems are situated" (p. 1351).

[iii] Interviewers are medical students trained in recruiting participants, obtaining informed consent, conducting quantitative interviews over the telephone, and quantitative data management.

For peer review only

For peer review only - http://bmjopen.bmj.com/site/about/guidelines.xhtml

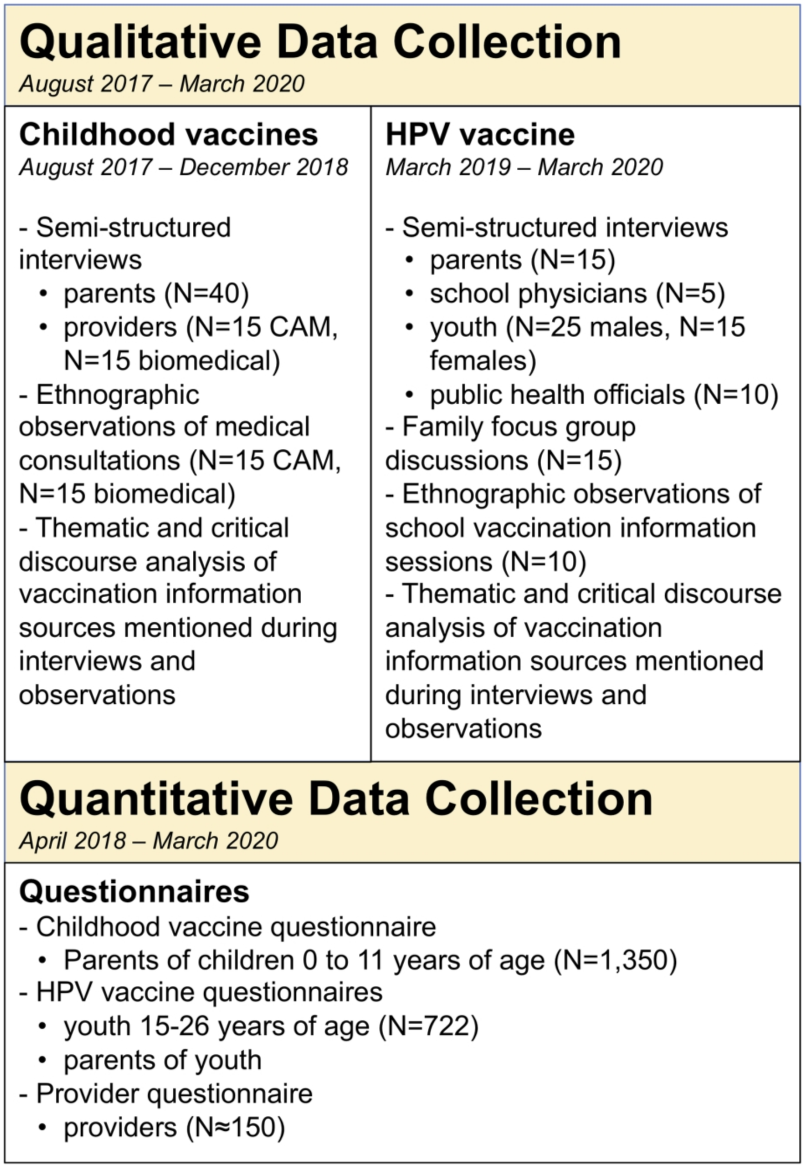

Study overview

156x212mm (300 x 300 DPI)

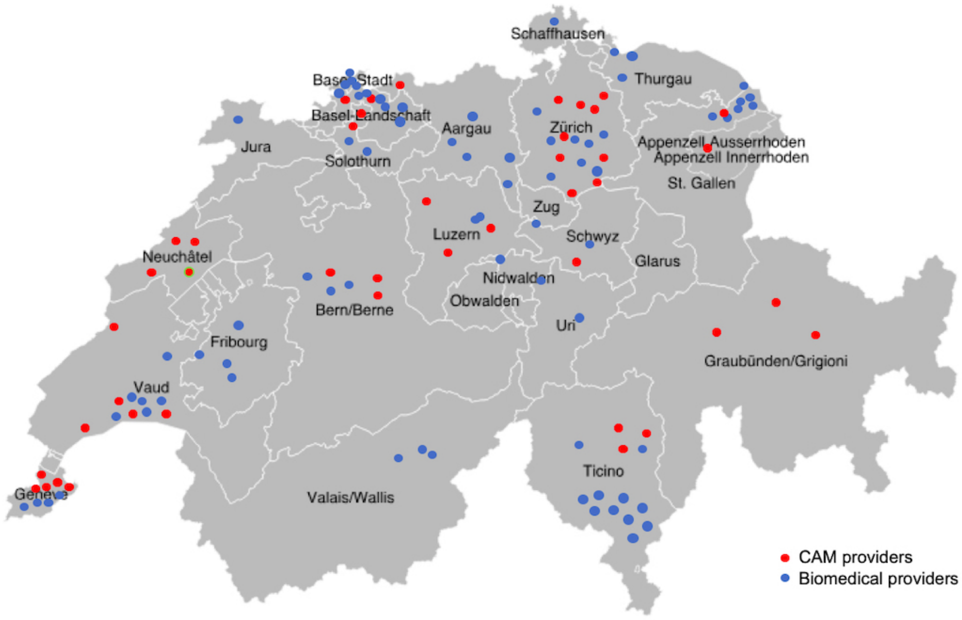

Network of participating providers

90x59mm (300 x 300 DPI)

Appendix 1

This appendix presents the questions we developed based on preliminary findings from the qualitative phase of research by theme.

For both the childhood and the HPV vaccination questionnaires, we developed questions addressing four themes.

Theme 1: Communication about vaccination with medical providers

Q1

Have you ever discussed childhood vaccination with your child`s doctor?	1. yes<br>2. no<br>3. doesn't want to disclose<br>4. doesn't know<br>5. missing	Avez-vous déjà discuté de la vaccination pour enfants avec le médecin de votre enfant ?	1. oui<br>2. non<br>3. ne veut pas divulguer<br>4. ne sait pas<br>5. missing
Haben Sie je eine Impfung für Kinder mit dem Arzt/ der Ärztin Ihres Kindes besprochen?	1. ja<br>2. nein<br>3. möchte die Frage nicht beantworten<br>4. weiss nicht<br>5. keine Antwort	Le è già capitato di discutere della vaccinazione infantile con il medico suo/a figlio/a?	1. si<br>2. no<br>3. non vuole rivelare<br>4. non sa<br>5. mancante
Asked in:	✓ Childhood vaccinations		

Have you ever discussed HPV vaccination with your teen`s doctor?	1. yes<br>2. no<br>3. doesn't want to disclose<br>4. doesn't know<br>5. missing	Avez-vous déjà discuté de la vaccination HPV avec le médecin de votre enfant?	1. oui<br>2. non<br>3. ne veut pas divulguer<br>4. ne sait pas<br>5. missing
Haben Sie je eine HPV Impfung mit dem Arzt/ der Ärztin Ihres Kindes besprochen?	1. ja<br>2. nein<br>3. möchte die Frage nicht beantworten<br>4. weiss nicht<br>5. keine Antwort	Le è già capitato di discutere della vaccinazione contro l`HPV con il medico di suo/a figlio/a?	1. si<br>2. no<br>3. non vuole rivelare<br>4. non sa<br>5. mancante
Asked in:	✓ HPV parent		

If yes of Q1, ask Q2-5

Q2

For peer review only - http://bmjopen.bmj.com/site/about/guidelines.xhtml

How strongly does your child`s doctor recommend vaccinating your child with all the recommended vaccines?<br><br>READ OUT ANSWER OPTIONS	1. Supports all recommended vaccines<br>2. Supports most recommended vaccines<br>3. Supports some recommended vaccines<br>4. Doesn't support any recommended vaccines<br>5. doesn't want to disclose<br>6. doesn't know<br>7. missing	Comment est-ce que vous caractériseriez le niveau de soutien au médecin de votre enfant votre enfant pour les vaccins? Est-il/elle…<br><br>LIRE TOUTES LES REPONSES POSSIBLES	1. Soutient toutes les vaccins recommandés<br>2. Soutient la plupart des vaccins recommandés<br>3. Soutient quelques-uns des vaccins recommandés<br>4. Ne soutient aucun vaccin recommandé<br>5. ne veut pas divulguer<br>6. ne sait pas<br>7. missing
Wie würden Sie das Niveau der Unterstützung/ die Unterstützung für Impfungen vom Arzt/ von der Ärztin Ihres Kindes einschätzen? Ist er/sie...<br><br>ANTWORTMÖGLICHKEITEN VORLESEN	1. Unterstützt alle empfohlenen Impfungen<br>2. Unterstützt die meisten empfohlenen Impfungen<br>3. Unterstützt ein paar der empfohlenen Impfungen<br>4. Unterstützt gar keine Impfungen<br>5. möchte die Frage nicht beantworten<br>6. weiss nicht<br>7. keine Antwort	Come qualificherebbe il livello di sostegno del medico di suo/a figlio/a alla vaccinazione?<br><br>LEGGERE LE RISPOSTE POSSIBILI	1. Sostiene tutti i vaccini raccomandati<br>2. Sostiene la maggior parte dei vaccini raccomandati<br>3. Sostiene alcuni dei vaccini raccomandati<br>4. Non sostiene alcun vaccino raccomandato<br>5. non vuole rivelare<br>6. non sa<br>7. mancante

Asked in:	✓ Childhood vaccinations

How strongly does your teen`s doctor recommend vaccinating your	1. Supports all recommended vaccines	Comment est-ce que vous caractériseriez le niveau de soutien	1. Soutient toutes les vaccins recommandés

For peer review only

teenager with all the recommended vaccines?<br><br>READ OUT ANSWER OPTIONS	2. Supports most recommended vaccines<br>3. Supports some recommended vaccines<br>4. Doesn't support any recommended vaccines<br>5. doesn't want to disclose<br>6. doesn't know<br>7. missing	au médecin de votre enfant votre enfant pour les vaccins? Est-il/elle…<br><br>LIRE TOUTES LES REPONSES POSSIBLES	2. Soutient la plupart des vaccins recommandés<br>3. Soutient quelques-uns des vaccins recommandés<br>4. Ne soutient aucun vaccin recommandé<br>5. ne veut pas divulguer<br>6. ne sait pas<br>7. missing
Wie würden Sie das Niveau der Unterstützung/ die Unterstützung für Impfungen vom Arzt/ von der Ärztin Ihres Kindes einschätzen? Ist er/sie...<br><br>ANTWORTMÖGLICHKEITEN VORLESEN	1. Unterstützt alle empfohlenen Impfungen<br>2. Unterstützt die meisten empfohlenen Impfungen<br>3. Unterstützt ein paar der empfohlenen Impfungen<br>4. Unterstützt gar keine Impfungen<br>5. möchte die Frage nicht beantworten<br>6. weiss nicht<br>7. keine Antwort	Come qualificherebbe il livello di sostegno del medico di suo/a figlio/a alla vaccinazione?<br><br>LEGGERE LE RISPOSTE POSSIBILI	1. Sostiene tutti i vaccini raccomandati<br>2. Sostiene la maggior parte dei vaccini raccomandati<br>3. Sostiene alcuni dei vaccini raccomandati<br>4. Non sostiene alcun vaccino raccomandato<br>5. non vuole rivelare<br>6. non sa<br>7. mancante

Asked in:	✓ HPV parent

Q3

How important is following the recommended vaccination	1. Very important<br>2. Somewhat important<br>3. Not very important	Quelle importance a-t-il de suivre exactement le plan de	1. Très important<br>2. Relativement important<br>3. Peu important

For peer review only

schedule for your child`s doctor?<br><br>READ OUT ANSWER OPTIONS	4. Not important at all<br>5. doesn't want to disclose<br>6. doesn't know<br>7. missing	vaccination pour le médecin de votre enfant?<br><br>LIRE TOUTES LES REPONSES POSSIBLES	4. Pas du tout important<br>5. ne veut pas divulguer<br>6. ne sait pas<br>7. missing
Wie wichtig ist es für den Arzt/ die Ärztin Ihres Kindes, den Impfplan genau einzuhalten?<br><br>ANTWORTMÖGLICHKEITEN VORLESEN	1. Sehr wichtig<br>2. Eher wichtig<br>3. Eher unwichtig<br>4. Gar nicht wichtig<br>5. möchte die Frage nicht beantworten<br>6. weiss nicht<br>7. keine Antwort	Quanto ritiene sia importante per il medico di suo/a figlio/a di seguire esattamente il programma di vaccinazione?<br><br>LEGGERE LE RISPOSTE POSSIBILI	1. Molto importante<br>2. Abbastanza importante<br>3. Non molto importante<br>4. Per niente importante<br>5. non vuole rivelare<br>6. non sa<br>7. mancante

Asked in:	✓ Childhood vaccinations

How important is following the recommended vaccination schedule for your child`s doctor?<br><br>READ OUT ANSWER OPTIONS	1. Very important<br>2. Somewhat important<br>3. Not very important<br>4. Not important at all<br>5. doesn't want to disclose<br>6. doesn't know<br>7. missing	Quelle importance a-t-il de suivre exactement le plan de vaccination pour le médecin de votre enfant?<br><br>LIRE TOUTES LES REPONSES POSSIBLES	1. Très important<br>2. Relativement important<br>3. Peu important<br>4. Pas du tout important<br>5. ne veut pas divulguer<br>6. ne sait pas<br>7. missing
Wie wichtig ist es für den Arzt/ die Ärztin Ihres Kindes, den Impfplan genau einzuhalten?	1. Sehr wichtig<br>2. Eher wichtig<br>3. Eher unwichtig<br>4. Gar nicht wichtig<br>5. möchte die Frage nicht beantworten<br>6. weiss nicht<br>7. keine Antwort	Quanto ritiene sia importante per il medico di suo/a figlio/a di seguire esattamente il programma di vaccinazione?	1. Molto importante<br>2. Abbastanza importante<br>3. Non molto importante<br>4. Per niente importante

For peer review only

ANTWORTMÖGLICHKEITEN VORLESEN		LEGGERE LE RISPOSTE POSSIBILI	5. non vuole rivelare<br>6. non sa<br>7. mancante
Asked in:	✓ HPV parent		

Q4

How much do you trust the information your child`s doctor gave you?<br><br>READ OUT ANSWER OPTIONS	1. Completely trust<br>2. Somewhat trust<br>3. Neither trust nor distrust<br>4. Somewhat distrust<br>5. Don't trust at all<br>6. doesn't want to disclose<br>7. doesn't know<br>8. missing	A quel point faites-vous confiance aux informations que au médecin de votre enfant vous a données?<br><br>LIRE TOUTES LES REPONSES POSSIBLES	1. Fait entièrement confiance<br>2. Fait un peu confiance<br>3. Ne fait ni confiance, ni ne fait pas confiance<br>4. Ne fait pas vraiment confiance<br>5. Ne fait pas du tout confiance<br>6. ne veut pas divulguer<br>7. ne sait pas<br>8. missing
Wie sehr vertrauen Sie der Information die der Arzt/ die Ärztin Ihres KindesIhnen gegeben hat?<br><br>ANTWORTMÖGLICHKEITEN VORLESEN	1. Ich habe sehr grosses Vertrauen<br>2. Ich habe einiges Vertrauen<br>3. Weder Vertrauen noch Misstrauen<br>4. Ich habe etwas Misstrauen<br>5. Ich habe gar kein Vertrauen<br>6. möchte die Frage nicht beantworten<br>7. weiss nicht<br>8. keine Antwort	Quanto si fida delle informazioni che il medico di suo/a figlio/a le ha dato?<br><br>LEGGERE LE RISPOSTE POSSIBILI	1. Si fida completamente<br>2. Si fida in parte<br>3. Né si fida né diffida<br>4. Diffida in parte<br>5. Diffida completamente<br>6. non vuole rivelare<br>7. non sa<br>8. mancante
Asked in:	✓ Childhood vaccinations		

For peer review only

How much do you trust the information your teen`s doctor gave you?<br><br>READ OUT ANSWER OPTIONS	1. Completely trust<br>2. Somewhat trust<br>3. Neither trust nor distrust<br>4. Somewhat distrust<br>5. Don't trust at all<br>6. doesn't want to disclose<br>7. doesn't know<br>8. missing	A quel point faites-vous confiance aux informations que le médecin de votre enfant vous a données?<br><br>LIRE TOUTES LES REPONSES POSSIBLES	1. Fait entièrement confiance<br>2. Fait un peu confiance<br>3. Ne fait ni confiance, ni ne fait pas confiance<br>4. Ne fait pas vraiment confiance<br>5. Ne fait pas du tout confiance<br>6. ne veut pas divulguer<br>7. ne sait pas<br>8. missing
Wie sehr vertrauen Sie der Information die der Arzt/ die Ärztin Ihres Kindes Ihnen gegeben hat?<br><br>ANTWORTMÖGLICHKEITEN VORLESEN	1. Ich habe sehr grosses Vertrauen<br>2. Ich habe einiges Vertrauen<br>3. Weder Vertrauen noch Misstrauen<br>4. Ich habe etwas Misstrauen<br>5. Ich habe gar kein Vertrauen<br>6. möchte die Frage nicht beantworten<br>7. weiss nicht<br>8. keine Antwort	Quanto si fida delle informazioni che il medico di suo figlio/a le ha dato?<br><br>LEGGERE LE RISPOSTE POSSIBILI	1. Si fida completamente<br>2. Si fida in parte<br>3. Né si fida né diffida<br>4. Diffida in parte<br>5. Diffida completamente<br>6. non vuole rivelare<br>7. non sa<br>8. mancante

Asked in:	✓ HPV parent

Q5

How satisfied were you with your discussions about vaccines with your child`s doctor?	1. Not at all satisfied<br>2. Somewhat satisfied<br>3. Neither satisfied nor unsatisfied<br>4. Satisfied<br>5. Very satisfied<br>6. doesn't want to disclose<br>7. doesn't know	A quel point avez-vous été satisfait.e des discussions sur les vaccins avec le médecin de votre enfant?	1. Pas du tout satisfait.e<br>2. Un peu satisfait.e<br>3. Ni satisfait.e, ni insatisfait.e<br>4. Satisfait.e<br>5. Très satisfait.e<br>6. Ne veut pas divulguer

For peer review only

READ OUT ANSWER OPTIONS	8. missing	LIRE TOUTES LES REPONSES POSSIBLES	7. ne sait pas<br>8. missing
Wie zufrieden sind Sie mit den Diskussionen über Impfungen mit dem Arzt/ der Ärztin Ihres Kindes?<br><br>ANTWORTMÖGLICHKEITEN VORLESEN	1. überhaupt nicht zufrieden<br>2. einigermassen zufrieden<br>3. weder zufrieden noch unzufrieden<br>4. zufrieden<br>5. sehr zufrieden<br>6. möchte die Frage nicht beantworten<br>7. weiss nicht<br>8. keine Antwort	Quanto è soddisfatto/a delle discussioni che ha avuto sui vaccini con il medico di suo/a figlio/a?<br><br>LEGGERE LE RISPOSTE POSSIBILI	1. Per niente soddisfatto<br>2. In parte soddisfatto<br>3. Né soddisfatto né insoddisfatto<br>4. Soddisfatto<br>5. Molto soddisfatto<br>6. non vuole rivelare<br>7. non sa<br>8. mancante

Asked in:	✓ Childhood vaccinations

How satisfied were you with your discussions about vaccines with your teen`s doctor?<br><br>READ OUT ANSWER OPTIONS	9. Not at all satisfied<br>10. Somewhat satisfied<br>11. Neither satisfied nor unsatisfied<br>12. Satisfied<br>13. Very satisfied<br>14. doesn't want to disclose<br>15. doesn't know<br>16. missing	A quel point avez-vous été satisfait.e des discussions sur les vaccins avec le médecin de votre enfant?<br><br>LIRE TOUTES LES REPONSES POSSIBLES	9. Pas du tout satisfait.e<br>10. Un peu satisfait.e<br>11. Ni satisfait.e, ni insatisfait.e<br>12. Satisfait.e<br>13. Très satisfait.e<br>14. Ne veut pas divulguer<br>15. ne sait pas<br>16. missing
Wie zufrieden sind Sie mit den Diskussionen über Impfungen mit dem Arzt/ der Ärztin Ihres Kindes?<br><br>ANTWORTMÖGLICHKEITEN VORLESEN	9. überhaupt nicht zufrieden<br>10. einigermassen zufrieden<br>11. weder zufrieden noch unzufrieden<br>12. zufrieden<br>13. sehr zufrieden<br>14. möchte die Frage nicht beantworten<br>15. weiss nicht<br>16. keine Antwort	Quanto è soddisfatto/a delle discussioni che ha avuto sui vaccini con il medico di suo figlio/a?<br><br>LEGGERE LE RISPOSTE POSSIBILI	9. Per niente soddisfatto<br>10. In parte soddisfatto<br>11. Né soddisfatto né insoddisfatto<br>12. Soddisfatto<br>13. Molto soddisfatto<br>14. non vuole rivelare<br>15. non sa<br>16. mancante

For peer review only

Asked in:	✓ HPV parent

Q6

Please indicate how much you agree with the following statements:	Veuillez indiquer à quel point vous êtes d'accord ou en désaccord avec les énoncés suivants:
Bitte geben Sie an, wie sehr Sie den folgenden Aussagen zustimmen:	Per favore mi indichi quanto è d'accordo con le seguenti affermazioni:
Asked in:	✓ Childhood vaccinations<br>✓ HPV parent<br>✓ HPV adolescent

Q7

I am able to ask my child`s doctor questions about vaccination.<br><br>READ OUT ANSWER OPTIONS	1. Completely agree<br>2. Somewhat agree<br>3. Neither agree nor disagree<br>4. Somewhat disagree<br>5. Completely disagree<br>6. doesn't want to disclose<br>7. doesn't know<br>8. missing	Je suis capable de poser des questions sur la vaccination au médecin de mon enfant.<br><br>LIRE TOUTES LES REPONSES POSSIBLES	1. Tout à fait d'accord<br>2. Un peu d'accord<br>3. Ni en accord, ni en désaccord<br>4. Un peu en désacord<br>5. Pas du tout d'accord<br>6. ne veut pas divulguer<br>7. ne sait pas<br>8. missing
Ich kann dem Arzt/der Ärztin meines Kindes Fragen zu Impfungen stellen.<br><br>ANTWORTMÖGLICHKEITEN VORLESEN	1. stimme stark zu<br>2. stimme einigermassen zu<br>3. weder noch<br>4. stimme eher nicht zu<br>5. stimme überhaupt nicht zu<br>6. möchte die Frage nicht beantworten<br>7. weiss nicht<br>8. keine Antwort	Sono capace di porre delle domande riguardanti le vaccinazioni al medico di mio/a figlio/a.<br><br>LEGGERE LE RISPOSTE POSSIBILI	1. Completamente d'accordo<br>2. In parte d'accordo<br>3. Né d'accordo né in disaccordo<br>4. In parte in disaccordo<br>5. Completamente in disaccordo<br>6. non vuole rivelare

For peer review only

			7. non sa<br>8. mancante
Asked in:	✓ Childhood vaccinations		

I am able to ask my teen`s doctor questions about vaccination.<br><br>READ OUT ANSWER OPTIONS	1. Completely agree<br>2. Somewhat agree<br>3. Neither agree nor disagree<br>4. Somewhat disagree<br>5. Completely disagree<br>6. doesn't want to disclose<br>7. doesn't know<br>8. missing	Je suis capable de poser des questions sur la vaccination au médecin de mon adolescent.<br><br>LIRE TOUTES LES REPONSES POSSIBLES	1. Tout à fait d'accord<br>2. Un peu d'accord<br>3. Ni en accord, ni en désaccord<br>4. Un peu en désacord<br>5. Pas du tout d'accord<br>6. ne veut pas divulguer<br>7. ne sait pas<br>8. missing
Ich kann dem Arzt/der Ärztin meines Kindes Fragen zu Impfungen stellen.<br><br>ANTWORTMÖGLICHKEITEN VORLESEN	1. stimme stark zu<br>2. stimme einigermassen zu<br>3. weder noch<br>4. stimme eher nicht zu<br>5. stimme überhaupt nicht zu<br>6. möchte die Frage nicht beantworten<br>7. weiss nicht<br>8. keine Antwort	Sono capace di porre delle domande riguardanti le vaccinazioni al medico di mio/a figlio/a.<br><br>LEGGERE LE RISPOSTE POSSIBILI	1. Completamente d'accordo<br>2. In parte d'accordo<br>3. Né d'accordo né in disaccordo<br>4. In parte in disaccordo<br>5. Completamente in disaccordo<br>6. non vuole rivelare<br>7. non sa<br>8. mancante
Asked in:	✓ HPV parent		

I am able to ask my doctor questions about vaccination.	1. Completely agree<br>2. Somewhat agree<br>3. Neither agree nor disagree<br>4. Somewhat disagree	Je suis capable de poser des questions sur la vaccination à mon médecin.	1. Tout à fait d'accord<br>2. Un peu d'accord<br>3. Ni en accord, ni en désaccord

For peer review only

READ OUT ANSWER OPTIONS	5. Completely disagree<br>6. doesn't want to disclose<br>7. doesn't know<br>8. missing	LIRE TOUTES LES REPONSES POSSIBLES	4. Un peu en désacord<br>5. Pas du tout d'accord<br>6. ne veut pas divulguer<br>7. ne sait pas<br>8. missing
Ich kann meinem Arzt/meiner Ärztin Fragen zu Impfungen stellen.<br><br>ANTWORTMÖGLICHKEITEN VORLESEN	1. stimme stark zu<br>2. stimme einigermassen zu<br>3. weder noch<br>4. stimme eher nicht zu<br>5. stimme überhaupt nicht zu<br>6. möchte die Frage nicht beantworten<br>7. weiss nicht<br>8. keine Antwort	Sono capace di porre delle domande riguardanti le vaccinazioni al medico di mio/a figlio/a.<br><br>LEGGERE LE RISPOSTE POSSIBILI	1. Completamente d'accordo<br>2. In parte d'accordo<br>3. Né d'accordo né in disaccordo<br>4. In parte in disaccordo<br>5. Completamente in disaccordo<br>6. non vuole rivelare<br>7. non sa<br>8. mancante
Asked in:	✓ HPV adolescent		

Q8

My child`s doctor takes the time needed to discuss my concerns about vaccination with me.<br><br>READ OUT ANSWER OPTIONS	1. Completely agree<br>2. Somewhat agree<br>3. Neither agree nor disagree<br>4. Somewhat disagree<br>5. Completely disagree<br>6. doesn't want to disclose<br>7. doesn't know<br>8. missing	Le médecin de mon enfant prend le temps nécessaire pour discuter de mes questions et inquiétudes sur la vaccnation.<br><br>LIRE TOUTES LES REPONSES POSSIBLES	1. Tout à fait d'accord<br>2. Un peu d'accord<br>3. Ni en accord, ni en désaccord<br>4. Un peu en désacord<br>5. Pas du tout d'accord<br>6. ne veut pas divulguer<br>7. ne sait pas<br>8. missing
Der Arzt/ die Ärztin meines Kindes nimmt sich die nötige Zeit um	1. stimme stark zu<br>2. stimme einigermassen zu	Il medico di mio/a figlio/a consacra il tempo necessario per discutere delle	1. Completamente d'accordo

For peer review only

meine Fragen und Sorgen bezüglich Impfungen mit mir zu besprechen.<br><br>ANTWORTMÖGLICHKEITEN VORLESEN	3. weder noch<br>4. stimme eher nicht zu<br>5. stimme überhaupt nicht zu<br>6. möchte die Frage nicht beantworten<br>7. weiss nicht<br>8. keine Antwort	mie preoccupazioni riguardanti le vaccinazioni.<br><br>LEGGERE LE RISPOSTE POSSIBILI	2. In parte d'accordo<br>3. Né d'accordo né in disaccordo<br>4. In parte in disaccordo<br>5. Completamente in disaccordo<br>6. non vuole rivelare<br>7. non sa<br>8. mancante
Asked in:	✓ Childhood vaccinations		

My teen`s doctor takes the time needed to discuss my concerns about vaccination with me.<br><br>READ OUT ANSWER OPTIONS	1. Completely agree<br>2. Somewhat agree<br>3. Neither agree nor disagree<br>4. Somewhat disagree<br>5. Completely disagree<br>6. doesn't want to disclose<br>7. doesn't know<br>8. missing	Le médecin de mon enfant prend le temps nécessaire pour discuter de mes questions et inquiétudes sur la vaccnation.<br><br>LIRE TOUTES LES REPONSES POSSIBLES	1. Tout à fait d'accord<br>2. Un peu d'accord<br>3. Ni en accord, ni en désaccord<br>4. Un peu en désacord<br>5. Pas du tout d'accord<br>6. ne veut pas divulguer<br>7. ne sait pas<br>8. missing
Der Arzt/ die Ärztin meines Kindes nimmt sich die nötige Zeit um meine Fragen und Sorgen bezüglich Impfungen mit mir zu besprechen.<br><br>ANTWORTMÖGLICHKEITEN VORLESEN	1. stimme stark zu<br>2. stimme einigermassen zu<br>3. weder noch<br>4. stimme eher nicht zu<br>5. stimme überhaupt nicht zu<br>6. möchte die Frage nicht beantworten<br>7. weiss nicht<br>8. keine Antwort	Il medico di mio/a figlio/a consacra il tempo necessario per discutere delle mie preoccupazioni riguardanti le vaccinazioni.<br><br>LEGGERE LE RISPOSTE POSSIBILI	1. Completamente d'accordo<br>2. In parte d'accordo<br>3. Né d'accordo né in disaccordo<br>4. In parte in disaccordo<br>5. Completamente in disaccordo<br>6. non vuole rivelare<br>7. non sa

For peer review only

			8. mancante
Asked in:	✓ HPV parent		

Q9

My child`s doctor takes my concerns about vaccination seriously.<br><br>READ OUT ANSWER OPTIONS	1. Completely agree<br>2. Somewhat agree<br>3. Neither agree nor disagree<br>4. Somewhat disagree<br>5. Completely disagree<br>6. doesn't want to disclose<br>7. doesn't know<br>8. missing	Le médecin de mon enfant prend mes inquiétudes sur la vaccination au sérieux.<br><br>LIRE TOUTES LES REPONSES POSSIBLES	1. Tout à fait d'accord<br>2. Un peu d'accord<br>3. Ni en accord, ni en désaccord<br>4. Un peu en désacord<br>5. Pas du tout d'accord<br>6. ne veut pas divulguer<br>7. ne sait pas<br>8. missing
Der Arzt/ die Ärztin meines Kindes nimmt meine Sorgen bezüglich Impfungen ernst.<br><br>ANTWORTMÖGLICHKEITEN VORLESEN	1. stimme stark zu<br>2. stimme einigermassen zu<br>3. weder noch<br>4. stimme eher nicht zu<br>5. stimme überhaupt nicht zu<br>6. möchte die Frage nicht beantworten<br>7. weiss nicht<br>8. keine Antwort	Il medico di mio/a figlio/a prende sul serio le mie preoccupazioni riguardanti le vaccinazioni.<br><br>LEGGERE LE RISPOSTE POSSIBILI	1. Completamente d'accordo<br>2. In parte d'accordo<br>3. Né d'accordo né in disaccordo<br>4. In parte in disaccordo<br>5. Completamente in disaccordo<br>6. non vuole rivelare<br>7. non sa<br>8. mancante
Asked in:	✓ Childhood vaccinations		

My teen`s doctor takes my concerns about vaccination seriously.	1. Completely agree<br>2. Somewhat agree<br>3. Neither agree nor disagree<br>4. Somewhat disagree	Le médecin de mon enfant prend mes inquiétudes sur la vaccination au sérieux.	1. Tout à fait d'accord<br>2. Un peu d'accord<br>3. Ni en accord, ni en désaccord

For peer review only

READ OUT ANSWER OPTIONS	5. Completely disagree<br>6. doesn't want to disclose<br>7. doesn't know<br>8. missing	LIRE TOUTES LES REPONSES POSSIBLES	4. Un peu en désacord<br>5. Pas du tout d'accord<br>6. ne veut pas divulguer<br>7. ne sait pas<br>8. missing
Der Arzt/ die Ärztin meines Kindes nimmt meine Sorgen bezüglich Impfungen ernst.<br><br>ANTWORTMÖGLICHKEITEN VORLESEN	1. stimme stark zu<br>2. stimme einigermassen zu<br>3. weder noch<br>4. stimme eher nicht zu<br>5. stimme überhaupt nicht zu<br>6. möchte die Frage nicht beantworten<br>7. weiss nicht<br>8. keine Antwort	Il medico di mio/a figlio/a prende sul serio le mie preoccupazioni riguardanti le vaccinazioni.<br><br>LEGGERE LE RISPOSTE POSSIBILI	1. Completamente d'accordo<br>2. In parte d'accordo<br>3. Né d'accordo né in disaccordo<br>4. In parte in disaccordo<br>5. Completamente in disaccordo<br>6. non vuole rivelare<br>7. non sa<br>8. mancante
Asked in:	✓ HPV parent		

Q10

My child`s doctor`s views on vaccination are similar to my own.<br><br>READ OUT ANSWER OPTIONS	1. Completely agree<br>2. Somewhat agree<br>3. Neither agree nor disagree<br>4. Somewhat disagree<br>5. Completely disagree<br>6. doesn't want to disclose<br>7. doesn't know<br>8. missing	Les points de vue du médecin de mon enfant au sujet de la vaccination sont similaires aux miens.<br><br>LIRE TOUTES LES REPONSES POSSIBLES	1. Tout à fait d'accord<br>2. Un peu d'accord<br>3. Ni en accord, ni en désaccord<br>4. Un peu en désacord<br>5. Pas du tout d'accord<br>6. ne veut pas divulguer<br>7. ne sait pas<br>8. missing
Die Ansichten des Arztes/ der Ärztin meines Kindes	1. stimme stark zu<br>2. stimme einigermassen zu	Il punto di vista del medico di mio/a figlio/a sulla	1. Completamente d'accordo

For peer review only

entsprechen meinen eigenen.<br><br>ANTWORTMÖGLICHKEITEN VORLESEN	3. weder noch<br>4. stimme eher nicht zu<br>5. stimme überhaupt nicht zu<br>6. möchte die Frage nicht beantworten<br>7. weiss nicht<br>8. keine Antwort	vaccinazione è simile al mio.<br><br>LEGGERE LE RISPOSTE POSSIBILI	2. In parte d'accordo<br>3. Né d'accordo né in disaccordo<br>4. In parte in disaccordo<br>5. Completamente in disaccordo<br>6. non vuole rivelare<br>7. non sa<br>8. mancante
Asked in:	✓ Childhood vaccinations		

My teen`s doctor`s views on vaccination are similar to my own.<br><br>READ OUT ANSWER OPTIONS	1. Completely agree<br>2. Somewhat agree<br>3. Neither agree nor disagree<br>4. Somewhat disagree<br>5. Completely disagree<br>6. doesn't want to disclose<br>7. doesn't know<br>8. missing	Les points de vue du médecin de mon enfant au sujet de la vaccination sont similaires aux miens.<br><br>LIRE TOUTES LES REPONSES POSSIBLES	1. Tout à fait d'accord<br>2. Un peu d'accord<br>3. Ni en accord, ni en désaccord<br>4. Un peu en désacord<br>5. Pas du tout d'accord<br>6. ne veut pas divulguer<br>7. ne sait pas<br>8. missing
Die Ansichten des Arztes/ der Ärztin meines Kindes entsprechen meinen eigenen.<br><br>ANTWORTMÖGLICHKEITEN VORLESEN	1. stimme stark zu<br>2. stimme einigermassen zu<br>3. weder noch<br>4. stimme eher nicht zu<br>5. stimme überhaupt nicht zu<br>6. möchte die Frage nicht beantworten<br>7. weiss nicht<br>8. keine Antwort	Il punto di vista del medico di mio/a figlio/a sulla vaccinazione è simile al mio.<br><br>LEGGERE LE RISPOSTE POSSIBILI	1. Completamente d'accordo<br>2. In parte d'accordo<br>3. Né d'accordo né in disaccordo<br>4. In parte in disaccordo<br>5. Completamente in disaccordo<br>6. non vuole rivelare

For peer review only

			7. non sa<br>8. mancante
Asked in:	✓ HPV parent		

Q11

Have you discussed vaccination for your child with any other doctor?	1. Yes<br>2. No<br>3. doesn't want to disclose<br>4. doesn't know<br>5. missing	Avez-vous déjà discuté de la vaccination pour votre enfant avec d`autres médecins?	1. Oui<br>2. Non<br>3. ne veut pas divulguer<br>4. ne sait pas<br>5. missing
Haben Sie je die Impfungen Impfung für Ihr Kind mit einem anderen Arzt/ einer anderen Ärztin besprochen?	1. ja<br>2. nein<br>3. möchte die Frage nicht beantworten<br>4. weiss nicht<br>5. keine Antwort	Le è mai capitato di discutere della vaccinazione infantile per suo/a figlio/a con un altro medico?	1. Si<br>2. No<br>3. non vuole rivelare<br>4. non sa<br>5. mancante
Asked in:	✓ Childhood vaccinations		

Have you discussed HPV vaccination for your teen with any other doctor?	1. Yes<br>2. No<br>3. doesn't want to disclose<br>4. doesn't know<br>5. missing	Avez-vous déjà discuté de la vaccination HPV pour votre enfant avec d`autres médecins?	1. Oui<br>2. Non<br>3. ne veut pas divulguer<br>4. ne sait pas<br>5. missing
Haben Sie je die die HPV Impfung Impfung für Ihr Kind mit einem anderen Arzt/ einer anderen Ärztin besprochen?	1. ja<br>2. nein<br>3. möchte die Frage nicht beantworten<br>4. weiss nicht<br>5. keine Antwort	Le è mai capitato di discutere della vaccinazione contro l`HPV per suo/a figlio/a con un altro medico?	1. Si<br>2. No<br>3. non vuole rivelare<br>4. non sa<br>5. mancante
Asked in:	✓ HPV parent		

If Q11 = 1, ask Q12-14

Q12

For peer review only

What led you to consult another doctor?<br><br>READ OUT ANSWER OPTIONS	1. Second opinion<br>2. moved<br>3. former provider stopped working<br>4. disagreement with provider<br>5. Other:_______<br>6. doesn't want to disclose<br>7. doesn't know<br>8. missing	Pourquoi est-ce que vous avez consulté un autre médecin?<br><br>LIRE TOUTES LES REPONSES POSSIBLES	1. Deuxième opinion<br>2. a changé de résidence<br>3. l'ancien professionnel de la santé a arrêté de travailler<br>4. Différence d'opinion ou désaccord avec le professionnel de la santé<br>5. Autre:<br>6. ne veut pas divulguer<br>7. ne sait pas<br>8. missing
Was hat Sie dazu bewogen, einen anderen Arzt/ eine andere Ärztin aufzusuchen ?<br><br>ANTWORTMÖGLICHKEITEN VORLESEN	1. Zweitmeinung<br>2. Umgezogen<br>3. Früherer Arzt/Ärztin praktiziert nicht mehr<br>4. Uneinigkeit mit Arzt/Ärztin<br>5. Anderes:_____<br>6. möchte die Frage nicht beantworten<br>7. weiss nicht<br>8. keine Antwort	Che cosa l'ha portata a consultare un altro medico?<br><br>LEGGERE LE RISPOSTE POSSIBILI	1. Secondo parere<br>2. spostato<br>3. Il precedente medico/operatore della salute ha smesso di lavorare<br>4. Disaccordo con il medico/operatore della salute<br>5. Altro:_____<br>6. non vuole rivelare<br>7. non sa<br>8. mancante
Asked in:	✓ Childhood vaccinations<br>✓ HPV parent		

Q13

How satisfied were you with your	1. Not at all satisfied	A quel point étiez-vous satisfait.e de	1. Pas du tout satisfait.e

discussions about vaccines with that doctor?<br><br>READ OUT ANSWER OPTIONS	2. Somewhat satisfied<br>3. Neither satisfied nor unsatisfied<br>4. Satisfied<br>5. Very satisfied<br>6. doesn't want to disclose<br>7. doesn't know<br>8. missing	vos discussions sur les vaccins avec ce médecin?<br><br>LIRE TOUTES LES REPONSES POSSIBLES	2. Un peu satisfait.e<br>3. Ni satisfait.e, ni insatisfait.e<br>4. Satisfait.e<br>5. Très satisfait.e<br>6. Ne veut pas divulguer<br>7. ne sait pas<br>8. missing
Wie zufrieden waren Sie mit der Besprechung über Impfungen mit diesem Arzt/ dieser Ärztin?<br><br>ANTWORTMÖGLICHKEITEN VORLESEN	1. überhaupt nicht zufrieden<br>2. einigermassen zufrieden<br>3. weder zufrieden noch unzufrieden<br>4. zufrieden<br>5. sehr zufrieden<br>6. möchte die Frage nicht beantworten<br>7. weiss nicht<br>8. keine Antwort	Quanto è soddisfatto/a delle discussioni che ha avuto sul vaccino sulla vaccinazione infantile con quest`altro medico?<br><br>LEGGERE LE RISPOSTE POSSIBILI	1. überhaupt nicht zufrieden<br>2. einigermassen zufrieden<br>3. weder zufrieden noch unzufrieden<br>4. zufrieden<br>5. sehr zufrieden<br>6. möchte die Frage nicht beantworten<br>7. weiss nicht<br>8. keine Antwort
Asked in:		✓ Childhood vaccinations	

How satisfied were you with your discussions about HPV vaccines with that doctor?<br><br>READ OUT ANSWER OPTIONS	1. Not at all satisfied<br>2. Somewhat satisfied<br>3. Neither satisfied nor unsatisfied<br>4. Satisfied<br>5. Very satisfied<br>6. doesn't want to disclose<br>7. doesn't know<br>8. missing	A quel point étiez-vous satisfait.e de vos discussions sur les vaccins HPV avec ce médecin?<br><br>LIRE TOUTES LES REPONSES POSSIBLES	1. Pas du tout satisfait.e<br>2. Un peu satisfait.e<br>3. Ni satisfait.e, ni insatisfait.e<br>4. Satisfait.e<br>5. Très satisfait.e<br>6. Ne veut pas divulguer<br>7. ne sait pas<br>8. missing
Wie zufrieden waren Sie mit der	1. überhaupt nicht zufrieden	Quanto è soddisfatto/a delle	1. überhaupt nicht zufrieden

For peer review only

Besprechung über HPV Impfungen mit diesem Arzt/ dieser Ärztin?<br><br>ANTWORTMÖGLICHKEITEN VORLESEN	2. einigermassen zufrieden<br>3. weder zufrieden noch unzufrieden<br>4. zufrieden<br>5. sehr zufrieden<br>6. möchte die Frage nicht beantworten<br>7. weiss nicht<br>8. keine Antwort	discussioni che ha avuto sul vaccino sul vaccino contro l`HPV con quest`altro medico?<br><br>LEGGERE LE RISPOSTE POSSIBILI	2. einigermassen zufrieden<br>3. weder zufrieden noch unzufrieden<br>4. zufrieden<br>5. sehr zufrieden<br>6. möchte die Frage nicht beantworten<br>7. weiss nicht<br>8. keine Antwort
Asked in:		✓ HPV parent	

Q14

How much do you trust the information that doctor gave you about vaccines?<br><br>READ OUT ANSWER OPTIONS	1. Completely trust<br>2. Somewhat trust<br>3. Neither trust nor distrust<br>4. Somewhat distrust<br>5. Don't trust at all<br>6. doesn't want to disclose<br>7. doesn't know<br>8. missing	A quel point faites-vous confiance aux informations au sujet des vaccins que ce médecin vous a données?<br><br>LIRE TOUTES LES REPONSES POSSIBLES	1. Fait entièrement confiance<br>2. Fait un peu confiance<br>3. Ne fait ni confiance, ni ne fait pas confiance<br>4. Ne fait pas vraiment confiance<br>5. ne fait pas du tout confiance<br>6. ne veut pas divulguer<br>7. ne sait pas<br>8. missing
Wie sehr haben Sie der Information zu Impfungenvertraut, die dieser Arzt/ diese Ärztin Ihnen gegeben hat?	1. Ich habe grosses Vertrauen<br>2. Ich habe etwas Vertrauen<br>3. Weder Vertrauen noch Misstrauen<br>4. Ich habe etwas Misstrauen	Quanto si fida delle informazioni che questo medico le ha dato?<br><br>LEGGERE LE RISPOSTE POSSIBILI	1. Si fida completamente<br>2. Si fida in parte<br>3. Né si fida né diffida<br>4. Diffida in parte

For peer review only

ANTWORTMÖGLICHKEITEN VORLESEN	5. Ich habe gar kein Vertrauen<br>6. möchte die Frage nicht beantworten<br>7. weiss nicht<br>8. keine Antwort		5. Diffida completamente<br>6. non vuole rivelare<br>7. non sa<br>8. mancante
Asked in:	✓ Childhood vaccinations<br>✓ HPV parent		

Theme 2: Information sources consulted in vaccination decision-making

Q1

What are your most trusted information sources on vaccination?<br><br>MULTIPLE OPTIONS, DO NOT READ OUT LOUD	1. no information/no source<br>2. Family<br>3. my child's doctor<br>4. other health professional<br>5. Friends and acquaintances<br>6. Public health authorities<br>7. TV<br>8. Internet searches<br>9. Social media (such as Facebook, Instagram and Twitter)<br>10. Print media (such as books, magazines and newspapers)<br>11. Other:__________<br>12. doesn't want to disclose<br>13. doesn't know<br>14. missing	Quelles sont vos sources d'informations les plus fiables sur la vaccination?<br><br>PLUSIEURS OPTIONS, NE PAS LIRE TOUTES LES REPONSES POSSIBLES	1. aucune information/ aucune source<br>2. famille<br>3. le médecin de mon enfant<br>4. un autre profesionnel de la santé<br>5. Des ami.e.s ou connaissances<br>6. Les autorités de la santé publique (p. ex. L'Office fédéral de la santé publique)<br>7. La télévision<br>8. Des recherches sur Internet<br>9. Les réseaux sociaux (tels que Facebook, Instagram et Twitter)<br>10. la presse écrite (p.

For peer review only

			ex. livres, magazines et journaux)<br>11. Autre:__________<br>12. ne veut pas divulguer<br>13. ne sait pas<br>14. missing
Was sind Ihre zuverlässigsten Informationsquellen zur Impfung?<br><br>VERSCHIEDENE OPTIONEN- NICHT LAUT VORLESEN	1. Keine Informationsquelle<br>2. Familie<br>3. Arzt/Ärztin meines Kindes<br>4. Andere Gesundheitsfachperson<br>5. Freunde und Bekannte<br>6. Gesundheitsbehörden<br>7. TV<br>8. Internet-Recherchen<br>9. Soziale Medien (wie z.B. Facebook, Instagram, Twitter)<br>10. Print Medien (wie z.B. Zeitungen, Broschüren)<br>11. Anderes:__________<br>12. möchte die Frage nicht beantworten<br>13. weiss nicht<br>14. keine Antwort	Quali sono le fonti più affidabili di informazioni sulla vaccinazione?<br><br>NON LEGGERE LE RISPOSTE POSSIBILI	1. nessuna informazione/ nessuna fonte<br>2. Famiglia<br>3. il medico di mio figlio<br>4. altro operatore della salute<br>5. Amici e conoscenti<br>6. Autorità della salute pubblica<br>7. TV<br>8. Ricerche internet<br>9. Social media (come Facebook, Instagram e Twitter)<br>10. Media stampati (come libri, riviste o giornali)<br>11. Altro:__________<br>12. non vuole rivelare<br>13. non sa<br>14. mancante

Asked in:	✓ Childhood vaccinations<br>✓ HPV parent<br>✓ HPV adolescent

If Q1 = 7, ask Q2

Q2

Which TV programs?<br><br>DO NOT READ OUT UNLESS NECESSARY		Quels programmes télévisés?<br><br>DEMANDEZ A HAUTE VOIX UNIQUEMENT SI NECESSAIRE	
Welche Fernsehprogramme?<br><br>NICHT LAUT VORLESEN, WENN SIE NICHT ERFORDERLICH SIND		Quali programmi televisivi?<br><br>NON LEGGERE A MENO CHE NECESSARIO	

Asked in:	✓ Childhood vaccination<br>✓ HPV parent<br>✓ HPV adolescent

If Q1 = 8, ask Q3

Q3

Which websites?<br><br>DO NOT READ OUT UNLESS NECESSARY		Quels sites Web?<br><br>DEMANDEZ A HAUTE VOIX UNIQUEMENT SI NECESSAIRE	
Welches Websites?<br><br>NICHT LAUT VORLESEN, WENN SIE NICHT ERFORDERLICH SIND		Quali siti web?<br><br>NON LEGGERE A MENO CHE NECESSARIO	

For peer review only

Asked in:	✓ Childhood vaccination<br>✓ HPV parent<br>✓ HPV adolescent

If Q1 = 9, ask Q4

Q4

What social media?<br><br>DO NOT READ OUT UNLESS NECESSARY		Quels reseaux sociaux?<br><br>DEMANDEZ A HAUTE VOIX UNIQUEMENT SI NECESSAIRE	
Welche Sozial Medien?<br><br>NICHT LAUT VORLESEN, WENN SIE NICHT ERFORDERLICH SIND		Quali Social Media?<br><br>NON LEGGERE A MENO CHE NECESSARIO	

Asked in:	✓ Childhood vaccination<br>✓ HPV parent<br>✓ HPV adolescent

If Q1 = 10, ask Q5

Q5

What print media?<br><br>DO NOT READ OUT UNLESS NECESSARY		Quelles presses écrites?<br><br>DEMANDEZ A HAUTE VOIX UNIQUEMENT SI NECESSAIRE	
Welche Print Medien?		Quali media stampati?	

For peer review only

NICHT LAUT VORLESEN, WENN SIE NICHT ERFORDERLICH SIND		NON LEGGERE A MENO CHE NECESSARIO	

Asked in:	✓ Childhood vaccination<br>✓ HPV parent<br>✓ HPV adolescent

If Q8 = 11, ask Q6

Q6

What other sources?<br><br>DO NOT READ OUT UNLESS NECESSARY		Quelles autres sources?<br><br>DEMANDEZ A HAUTE VOIX UNIQUEMENT SI NECESSAIRE	
Welche anderen Quellen?<br><br>NICHT LAUT VORLESEN, WENN SIE NICHT ERFORDERLICH SIND		Quali altre fonti?<br><br>NON LEGGERE A MENO CHE NECESSARIO	

Asked in:	✓ Childhood vaccination<br>✓ HPV parent<br>✓ HPV adolescent

Q7

Did you apply the information you received when making decisions about vaccination (for your child)?	1. Yes<br>2. No<br>3. doesn't want to disclose<br>4. doesn't know<br>5. missing	Avez-vous considéré ces informations en prenant votre décision de vaccination (pour votre enfant?)	1. Oui<br>2. Non<br>3. ne veut pas divulguer<br>4. ne sait pas<br>5. missing

For peer review only

Hatten die erhaltenen Informationen Einfluss auf die Entscheidungen, welche sie über die Impfung (Ihres Kindes) gefällt haben?	1. ja<br>2. nein<br>3. möchte die Frage nicht beantworten<br>4. weiss nicht<br>5. keine Antwort	Ha considerato le informazioni che ha ricevuto quando si è trattato di decidere se vaccinare (suo/a figlio/a)?	1. Si<br>2. No<br>3. non vuole rivelare<br>4. non sa<br>5. mancante
Asked in:		✓ Childhood vaccinations<br>✓ HPV parent<br>✓ HPV adolescent	

Theme 3: Perceptions about risk and control concerning possible exposure to vaccine-preventable diseases

Q1

Please indicate how much you agree with the following statements:	Veuillez indiquer à quel point vous êtes d'accord ou en désaccord avec les énoncés suivants:
Bitte geben Sie an wie sehr Sie den folgenden Aussagen zustimmen:	Per favore indichi quanto è d'accordo con le seguenti affermazioni:
Asked in:	✓ Childhood vaccinations

Q2

I can always prevent my child from being infected with vaccine-preventable diseases by other means than vaccination.<br><br>READ OUT ANSWER OPTIONS	1. strongly agree<br>2. agree<br>3. neither agree nor disagree<br>4. disagree<br>5. Strongly disagree<br>6. doesn't want to disclose<br>7. doesn't know<br>8. missing	Je peux toujours prévenir que mon enfant soit infecté par des maladies pouvant être prévenues par un vaccin par autres moyens que de la vaccination.<br><br>LIRE TOUTES LES REPONSES POSSIBLES	1. Tout à fait d'accord<br>2. D'accord<br>3. Ni en accord, ni en désaccord<br>4. pas d'accord<br>5. Pas du tout d'accord<br>6. ne veut pas divulguer<br>7. ne sait pas<br>8. missing
Ich kann jederzeit verhindern, dass mein Kind sich mit	1. stimme stark zu<br>2. stimme zu<br>3. weder noch	Posso sempre prevenire che mio/a figlio/a si	1. Fortemente d'accordo<br>2. d'accordo

Krankheiten ansteckt, vor denen Impfungen schützen würden.<br><br>ANTWORTMÖGLICHKEITEN VORLESEN	4. stimme nicht zu<br>5. stimme gar nicht zu<br>6. möchte die Frage nicht beantworten<br>7. weiss nicht<br>8. keine Antwort	infetti con malattie prevenibili tramite vaccino.<br><br>LEGGERE LE RISPOSTE POSSIBILI	3. né d'accordo né in disaccordo<br>4. in disaccordo<br>5. Fortemente in disaccordo<br>6. non vuole rivelare<br>7. non sa<br>8. mancante
Asked in:	✓ Childhood vaccinations		

Q3

Vaccine-preventable diseases can be easily cured in Switzerland.<br><br>READ OUT ANSWER OPTIONS	1. strongly agree<br>2. agree<br>3. neither agree nor disagree<br>4. disagree<br>5. Strongly disagree<br>6. doesn't want to disclose<br>7. doesn't know<br>8. missing	En Suisse, on peut facilement guérir d'une maladie pouvant être prévenue par un vaccin.<br><br>LIRE TOUTES LES REPONSES POSSIBLES	1. Tout à fait d'accord<br>2. D'accord<br>3. Ni en accord, ni en désaccord<br>4. pas d'accord<br>5. Pas du tout d'accord<br>6. ne veut pas divulguer<br>7. ne sait pas<br>8. missing
Krankheiten, vor denen Impfungen schützen würden, können in der Schweiz einfach geheilt werden.<br><br>ANTWORTMÖGLICHKEITEN VORLESEN	1. stimme stark zu<br>2. stimme zu<br>3. weder noch<br>4. stimme nicht zu<br>5. stimme gar nicht zu<br>6. möchte die Frage nicht beantworten<br>7. weiss nicht<br>8. keine Antwort	In Svizzera, le malattie prevenibili tramite vaccino possono essere facilmente curate.<br><br>LEGGERE LE RISPOSTE POSSIBILI	1. Fortemente d'accordo<br>2. d'accordo<br>3. né d'accordo né in disaccordo<br>4. in disaccordo<br>5. Fortemente in disaccordo<br>6. non vuole rivelare<br>7. non sa<br>8. mancante
Asked in:	✓ Childhood vaccinations		

Q4

Vaccines can cause serious	1. strongly agree<br>2. agree	Les vaccins peuvent causer des problèmes	1. Tout à fait d'accord<br>2. D'accord

For peer review only

long-term harm to health.<br><br>READ OUT ANSWER OPTIONS	3. neither agree nor disagree<br>4. disagree<br>5. Strongly disagree<br>6. doesn't want to disclose<br>7. doesn't know<br>8. missing	sévères de santé à long-terme.<br><br>LIRE TOUTES LES REPONSES POSSIBLES	3. Ni en accord, ni en désaccord<br>4. pas d'accord<br>5. Pas du tout d'accord<br>6. ne veut pas divulguer<br>7. ne sait pas<br>8. missing
Impfungen können ernsthafte Langzeitschäden auslösen.<br><br>ANTWORTMÖGLICHKEITEN VORLESEN	1. stimme stark zu<br>2. stimme zu<br>3. weder noch<br>4. stimme nicht zu<br>5. stimme gar nicht zu<br>6. möchte die Frage nicht beantworten<br>7. weiss nicht<br>8. keine Antwort	I vaccini possono avere conseguenze gravi a lungo termine sulla salute.<br><br>LEGGERE LE RISPOSTE POSSIBILI	1. Fortemente d'accordo<br>2. d'accordo<br>3. né d'accordo né in disaccordo<br>4. in disaccordo<br>5. Fortemente in disaccordo<br>6. non vuole rivelare<br>7. non sa<br>8. mancante
Asked in:		✓ Childhood vaccinations	

Q5

Vaccination is unnatural, so it is best to vaccinate as little as possible.<br><br>READ OUT ANSWER OPTIONS	1. strongly agree<br>2. agree<br>3. neither agree nor disagree<br>4. disagree<br>5. Strongly disagree<br>6. doesn't want to disclose<br>7. doesn't know<br>8. missing	La vaccination n'est pas naturelle, donc, il est préférable de vacciner le moins possible.<br><br>LIRE TOUTES LES REPONSES POSSIBLES	1. Tout à fait d'accord<br>2. D'accord<br>3. Ni en accord, ni en désaccord<br>4. pas d'accord<br>5. Pas du tout d'accord<br>6. ne veut pas divulguer<br>7. ne sait pas<br>8. missing
Impfen ist unnatürlich, deshalb impfe ich so wenig wie möglich.	1. stimme stark zu<br>2. stimme zu<br>3. weder noch<br>4. stimme nicht zu<br>5. stimme gar nicht zu<br>6. möchte die Frage nicht beantworten	La vaccinazione è innaturale, dunque è meglio vaccinare il meno possibile.	1. Fortemente d'accordo<br>2. d'accordo<br>3. né d'accordo né in disaccordo<br>4. in disaccordo<br>5. Fortemente in disaccordo

For peer review only

ANTWORTMÖGLICHKEITEN VORLESEN	7. weiss nicht<br>8. keine Antwort	LEGGERE LE RISPOSTE POSSIBILI	6. non vuole rivelare<br>7. non sa<br>8. mancante
Asked in:		✓ Childhood vaccinations	

Q6

How likely do you think it is that your child will be exposed to vaccine-preventable diseases in your home?<br><br>READ OUT ANSWER OPTIONS	1. Very likely<br>2. Somewhat likely<br>3. Not sure<br>4. Somewhat unlikely<br>5. Very unlikely<br>6. doesn't want to disclose<br>7. doesn't know<br>8. missing	Selon vous, quelle est la probabilité que votre enfant soit exposé aux maladies pouvant être prévenues par un vaccin au sein de votre ménage?<br><br>LIRE TOUTES LES REPONSES POSSIBLES	1. Très probable<br>2. Un peu probable<br>3. Pas sûr.e<br>4. Un peu improbable<br>5. Très improbable<br>6. ne veut pas divulguer<br>7. ne sait pas<br>8. missing
Für wie wahrscheinlich halten Sie es, dass Ihr Kind bei Ihnen zuhause Krankheiten ausgesetzt sein wird, vor denen Impfungen schützen würden ?<br><br>ANTWORTMÖGLICHKEITEN VORLESEN	1. sehr wahrscheinlich<br>2. eher wahrscheinlich<br>3. unsicher<br>4. eher unwahrscheinlich<br>5. sehr unwahrscheinlich<br>6. möchte die Frage nicht beantworten<br>7. weiss nicht<br>8. keine Antwort	Quanto pensa sia probabile che suo/a figlio/a sia esposto/a a malattie prevenibili tramite vaccino in casa sua?<br><br>LEGGERE LE RISPOSTE POSSIBILI	1. Molto probabile<br>2. Parzialmente probabile<br>3. Non sicuro<br>4. Parzialmente improbabile<br>5. Molto improbabile<br>6. non vuole rivelare<br>7. non vuole rivelare<br>8. mancante
Asked in:		✓ Childhood vaccinations	

Q7

How likely do you think it is that your child will be exposed to	1. Very likely<br>2. Somewhat likely<br>3. Not sure	Selon vous, quelle est la probabilité que votre enfant soit exposé aux	1. Très probable<br>2. Un peu probable<br>3. Pas sûr.e

vaccine-preventable diseases in your community?<br><br>READ OUT ANSWER OPTIONS	4. Somewhat unlikely<br>5. Very unlikely<br>6. doesn't want to disclose<br>7. doesn't know<br>8. missing	maladies pouvant être prévenues par un vaccin au sein de votre communauté?<br><br>LIRE TOUTES LES REPONSES POSSIBLES	4. Un peu improbable<br>5. Très improbable<br>6. ne veut pas divulguer<br>7. ne sait pas<br>8. missing
Für wie wahrscheinlich halten Sie es dass Ihr Kind in Ihrem sozialen Umfeld Krankheiten ausgesetzt sein wird, vor denen Impfungen schützen würden ?<br><br>ANTWORTMÖGLICHKEITEN VORLESEN	1. sehr wahrscheinlich<br>2. eher wahrscheinlich<br>3. unsicher<br>4. eher unwahrscheinlich<br>5. sehr unwahrscheinlich<br>6. möchte die Frage nicht beantworten<br>7. weiss nicht<br>8. keine Antwort	Quanto pensa sia probabile che suo/a figlio/a sia esposto/a a malattie prevenibili tramite vaccino nella sua comunità?<br><br>LEGGERE LE RISPOSTE POSSIBILI	1. Molto probabile<br>2. Parzialmente probabile<br>3. Non sicuro<br>4. Parzialmente improbabile<br>5. Molto improbabile<br>6. non vuole rivelare<br>7. non vuole rivelare<br>8. mancante

Asked in:	✓ Childhood vaccinations

Q8

About how many of your family members with children do you think have vaccinated their children?<br><br>READ OUT ANSWER OPTIONS	1. Almost all<br>2. about three-quarters<br>3. about half<br>4. about a quarter<br>5. almost none<br>6. doesn't want to disclose<br>7. doesn't know<br>8. missing	D'après vous, à peu près combien des membres de votre famille avec des enfants ont fait vacciner leurs enfants?<br><br>LIRE TOUTES LES REPONSES POSSIBLES	1. Presque tous<br>2. Environ trois quarts<br>3. Environ la moitié<br>4. Environ un quart<br>5. Presqu'aucun<br>6. ne veut pas divulguer<br>7. ne sait pas<br>8. missing
Was denken Sie, Wie viele Ihrer Familienmitglieder	1. fast alle<br>2. etwa drei Viertel	Quanti membri della sua famiglia con figli pensa	1. Quasi tutto<br>2. circa tre quarti<br>3. circa la metà

mit Kindern haben ihre Kinder geimpft?<br><br>ANTWORTMÖGLICHKEITEN VORLESEN	3. etwa die Hälfte<br>4. etwa ein Viertel<br>5. fast niemand<br>6. möchte die Frage nicht beantworten<br>7. weiss nicht<br>8. keine Antwort	abbiano vaccinato i loro figli?<br><br>LEGGERE LE RISPOSTE POSSIBILI	4. circa un quarto<br>5. quasi nessuno<br>6. non vuole rivelare<br>7. non sa<br>8. mancante
Asked in:		✓ Childhood vaccinations	

Q9

About how many of your friends with children do you think have vaccinated their children?<br><br>READ OUT ANSWER OPTIONS	1. Almost all<br>2. about three-quarters<br>3. about half<br>4. about a quarter<br>5. almost none<br>6. doesn't want to disclose<br>7. doesn't know<br>8. missing	D'après vous, à peu près combien de vos amis ont fait vacciner leurs enfants?<br><br>LIRE TOUTES LES REPONSES POSSIBLES	1. Presque tous<br>2. Environ trois quarts<br>3. Environ la moitié<br>4. Environ un quart<br>5. Presqu'aucun<br>6. ne veut pas divulguer<br>7. ne sait pas<br>8. missing
Was denken Sie, Wie viele Ihrer Freunde mit Kindern haben Ihre Kinder geimpft?<br><br>ANTWORTMÖGLICHKEITEN VORLESEN	1. fast alle<br>2. etwa drei Viertel<br>3. etwa die Hälfte<br>4. etwa ein Viertel<br>5. fast niemand<br>6. möchte die Frage nicht beantworten<br>7. weiss nicht<br>8. keine Antwort	Quanti dei suoi amici con figli pensa abbiano vaccinato i loro figli?<br><br>LEGGERE LE RISPOSTE POSSIBILI	1. Quasi tutto<br>2. circa tre quarti<br>3. circa la metà<br>4. circa un quarto<br>5. quasi nessuno<br>6. non vuole rivelare<br>7. non sa<br>8. mancante
Asked in:		✓ Childhood vaccinations	

Q10

About how many of the children in your community do you think are vaccinated?	1. Almost all<br>2. about three-quarters<br>3. about half<br>4. about a quarter<br>5. almost none	D'après vous, a peu près combien des enfants au sein de votre	1. Presque tous<br>2. Environ trois quarts<br>3. Environ la moitié<br>4. Environ un quart

For peer review only

READ OUT ANSWER OPTIONS	6. doesn't want to disclose<br>7. doesn't know<br>8. missing	communauté sont vaccinés?<br><br>LIRE TOUTES LES REPONSES POSSIBLES	5. Presqu'aucun<br>6. ne veut pas divulguer<br>7. ne sait pas<br>8. missing
Was denken Sie, Wie viele Kinder in Ihrem sozialen Umfeld sind geimpft?<br><br>ANTWORTMÖGLICHKEITEN VORLESEN	1. fast alle<br>2. etwa drei Viertel<br>3. etwa die Hälfte<br>4. etwa ein Viertel<br>5. fast niemand<br>6. möchte die Frage nicht beantworten<br>7. weiss nicht<br>8. keine Antwort	Quanti bambini della sua comunità pensa siano vaccinati?<br><br>LEGGERE LE RISPOSTE POSSIBILI	1. Quasi tutto<br>2. circa tre quarti<br>3. circa la metà<br>4. circa un quarto<br>5. quasi nessuno<br>6. non vuole rivelare<br>7. non sa<br>8. mancante
Asked in:		✓ Childhood vaccinations	

Theme 3: HPV specific questions

Q1

Now I want to ask you some questions about the HPV vaccine.	J'aimerais maintenant vous poser quelques questions sur le vaccin HPV.
Jetzt würde ich Ihnen gerne einige Fragen zur HPV Impfung stellen.	Ora vorrei porle alcune domande riguardo il vaccino contro l'HPV.
Asked in:	✓ HPV parent<br>✓ HPV adolescent

Q2

Have you heard of the HPV vaccine?	1. yes<br>2. no<br>3. doesn't want to disclose<br>4. doesn't know<br>5. missing	Avez-vous déjà entendu parler du vaccin HPV?	1. oui<br>2. non<br>3. ne veut pas divulguer<br>4. ne sait pas<br>5. missing
Haben Sie schon von der HPV Impfung gehört?	1. ja<br>2. nein<br>3. möchte die Frage nicht beantworten<br>4. weiss nicht<br>5. keine Antwort	Ha già sentito parlare del vaccino contro l'HPV?	1. si<br>2. no<br>3. non vuole rivelare<br>4. non sa<br>5. mancante

For peer review only

Asked in:	✓ HPV parent<br>✓ HPV adolescent

If Q2 = 1, then ask Q3

Q3

What is it for?<br><br>PROBE:<br>ANYTHING ELSE?		Pour quelles raisons ce vaccin est-il administré?<br><br>PROBER: D'autres choses?	
Wozu ist sie gut?<br><br>VERSUCHE:<br>NOCH ZU ETWAS ANDEREM		A cosa serve?<br><br>RILANCIO:<br>QUALCOS'ALTRO?	

Asked in:	✓ HPV parent<br>✓ HPV adolescent

If Q2 = 2, then ask Q4

Q4

Have you heard of the cervical cancer vaccine?	1. yes<br>2. no<br>3. doesn't want to disclose<br>4. doesn't know<br>5. missing	Avez-vous déjà entendu parler du vaccin contre les cancers du col de l'utérus?	1. oui<br>2. non<br>3. ne veut pas divulguer<br>4. ne sait pas<br>5. missing
Haben Sie schon von der Impfung gegen Gebärmutterhalskrebs gehört?	1. ja<br>2. nein<br>3. möchte die Frage nicht beantworten<br>4. weiss nicht<br>5. keine Antwort	Ha già sentito parlare del vaccino contro il cancro della cervice uterina?	1. si<br>2. no<br>3. non vuole rivelare<br>4. non sa<br>5. mancante

Asked in:	✓ HPV parent<br>✓ HPV adolescent

If Q2 or Q4 = 1, ask Q5-7

For peer review only

Q5

Has your teen received HPV vaccine?	1. Yes<br>2. no<br>3. doesn't want to disclose<br>4. doesn't know<br>5. missing	Votre enfant, a-t-il.elle reçu le vaccin HPV?	1. oui<br>2. non<br>3. ne veut pas divulguer<br>4. ne sait pas<br>5. missing
Hat Ihr Kind als Jugendliche/r die HPV Impfung erhalten?	1. ja<br>2. nein<br>3. möchte die Frage nicht beantworten<br>4. weiss nicht<br>5. keine Antwort	Suo/a figlio/a ha ricevuto il vaccino contro l`HPV?	1. si<br>2. no<br>3. non vuole rivelare<br>4. non sa<br>5. mancante
Asked in:	✓ HPV parent		

For peer review only

Have you received HPV vaccine?	1. yes<br>2. no<br>3. doesn't want to disclose<br>4. doesn't know<br>5. missing	Avez-vous reçu le vaccin HPV?	1. oui<br>2. non<br>3. ne veut pas divulguer<br>4. ne sait pas<br>5. missing
Haben Sie die HPV Impfung erhalten?	1. ja<br>2. nein<br>3. möchte die Frage nicht beantworten<br>4. weiss nicht<br>5. keine Antwort	Ha ricevuto il vaccino contro l`HPV?	1. si<br>2. no<br>3. non vuole rivelare<br>4. non sa<br>5. mancante
Asked in:	✓ HPV adolescent		

If Q5 = 1, ask Q6

Q6

Where did your teen receive HPV vaccine?		Où est-ce que votre enfant a reçu le vaccin HPV?	
Wo hat Ihr Kind als Jugendliche/r die HPV Impfung bekommmen?		Dove ha ricevuto suo/a figlio/a il vaccino contro l`HPV?	
Asked in:	✓ HPV parent		

Where did you receive HPV vaccine?		Où est-ce que vous avez reçu le vaccin HPV?	
Wo haben Sie die HPV Impfung bekommmen?		Dove ha ricevuto 0 il vaccino contro l`HPV?	
Asked in:	✓ HPV adolescent		

Q7

Where have you heard about HPV vaccination?		Comment avez-vous entendu parler du vaccin HPV?	
Wo haben Sie von der HPV Impfung gehört?		Dove ha sentito parlare della vaccinazione HPV?	
Asked in:	✓ HPV parent<br>✓ HPV adolescent		

Q8

Was the HPV vaccine recommended by your teen`s school?	1. yes<br>2. no<br>3. doesn't want to disclose<br>4. doesn't know<br>5. missing	Est-ce que l'école de votre enfant a recommandé le vaccin HPV?	1. oui<br>2. non<br>3. ne veut pas divulguer<br>4. ne sait pas<br>5. missing
Wurde die HPV Impfung in der Schule Ihres Kindes empfohlen?	1. ja<br>2. nein<br>3. möchte die Frage nicht beantworten<br>4. weiss nicht<br>5. keine Antwort	Il vaccino contro l'HPV per suo/a figlio/a le è stato raccomandato dalla scuola?	1. si<br>2. no<br>3. non vuole rivelare<br>4. non sa<br>5. mancante
Asked in:	✓ HPV parent		

Was the HPV vaccine recommended by your school?	1. yes<br>2. no<br>3. doesn't want to disclose<br>4. doesn't know<br>5. missing	Est-ce que votre école a recommandé le vaccin HPV?	1. oui<br>2. non<br>3. ne veut pas divulguer<br>4. ne sait pas<br>5. missing

For peer review only

Wurde die HPV Impfung in Ihrer Schule empfohlen?	1. ja<br>2. nein<br>3. möchte die Frage nicht beantworten<br>4. weiss nicht<br>5. keine Antwort	Il vaccino contro l'HPV le è stato raccomandato dalla scuola?	1. si<br>2. no<br>3. non vuole rivelare<br>4. non sa<br>5. mancante
Asked in:	✓ HPV adolescent		

Q9

How do you feel about offering the HPV vaccine at school?	1. Very supportive<br>2. Somewhat supportive<br>3. Not sure<br>4. Somewhat unsupportive<br>5. Very unsupportive<br>6. doesn't want to disclose<br>7. doesn't know<br>8. missing	Quel est votre niveau de soutien pour l'offert de la vaccination HPV à l'école?	1. Très favorable<br>2. Un peu favorable<br>3. Pas sûr.e<br>4. Un peu défavorable<br>5. Très défavorable<br>6. ne veut pas divulguer<br>7. ne sait pas<br>8. missing
Wie finden sie es, dass die HPV Impfung über die Schule angeboten wird ?	1. sehr unterstützens wert<br>2. unterstützens wert<br>3. nicht sicher<br>4. nicht sehr unterstützens wert<br>5. nicht unterstützens wert<br>6. möchte die Frage nicht beantworten<br>7. weiss nicht<br>8. keine Antwort	Cosa ne pensa del fatto che il vaccino contro l'HPV venga proposto nelle scuole?	1. fortemente d'accordo<br>2. d'accordo<br>3. non sicuro/a<br>4. in disaccordo<br>5. fortemente in disaccordo<br>6. non vuole rivelare<br>7. non vuole rivelare<br>8. mancante
Asked in:	✓ HPV parent<br>✓ HPV adolescent		

Q10

Who did you consult with when deciding		Qui est-ce que vous avez consulté en	

For peer review only

whether or not to get the HPV vaccine for your teen?		décidant si vous alliez vacciner Votre enfant a-t-il.elle avec le vaccin HPV?	
Wen haben Sie konsultiert um zu entscheiden, ob Sie für Ihr Kind eine HPV Impfung machen lassen sollten?		Con chi si è consultato/a quando si è trattato di decidere se somministrare il vaccino contro l`HPV a suo/a figlio/a?	

Asked in:	✓ HPV parent

Who did you consult with when deciding whether or not to get the HPV vaccine?		Qui est-ce que vous avez consulté en décidant si vous alliez vous vacciner avec le vaccin HPV?	
Wen haben Sie konsultiert um zu entscheiden, ob Sie für haben Sie eine HPV Impfung machen lassen sollten?		Con chi si è consultato/a quando si è trattato di decidere se ricevere il vaccino contro l`HPV?	

Asked in:	✓ HPV adolescent

For peer review only

Theme 4: Parent-Provider Relationship

Q1

Do you agree or disagree with the following statements:	Veuillez indiquer à quel point vous êtes d'accord ou en désaccord avec les énoncés suivants:
Stimmen Sie den folgenden Aussagen zu?	È d'accordo o in disaccordo con le seguenti affermazioni:
Asked in:	✓ Childhood vaccinations

Q2

It is my responsibility as a parent to actively research health decisions for my child.<br><br>READ OUT ANSWER OPTIONS	1. strongly agree<br>2. agree<br>3. neither agree nor disagree<br>4. disagree<br>5. Strongly disagree<br>6. doesn't want to disclose<br>7. doesn't know<br>8. missing	En tant que parent, c'est ma responsabilité de me renseigner sur les décisions de santé pour mon enfant de manière active.<br><br>LIRE TOUTES LES REPONSES POSSIBLES	1. Tout à fait d'accord<br>2. D'accord<br>3. Ni en accord, ni en désaccord<br>4. pas d'accord<br>5. Pas du tout d'accord<br>6. ne veut pas divulguer<br>7. ne sait pas<br>8. missing
Es ist meine Verantwortung als Elternteil mich aktiv über gesundheitliche Entscheidungen für mein Kind zu informieren.<br><br>ANTWORTMÖGLICHKEITEN VORLESEN	1. stimme stark zu<br>2. stimme zu<br>3. weder noch<br>4. stimme nicht zu<br>5. stimme gar nicht zu<br>6. möchte die Frage nicht beantworten<br>7. weiss nicht<br>8. keine Antwort	È mia responsabilità di genitore informarmi attivamente sulle decisioni che riguardano la salute di mio/a figlio/a.<br><br>LEGGERE LE RISPOSTE POSSIBILI	1. Fortemente d'accordo<br>2. d'accordo<br>3. né d'accordo né in disaccordo<br>4. in disaccordo<br>5. Fortemente in disaccordo<br>6. non vuole rivelare<br>7. non sa<br>8. mancante

Asked in:	✓ Childhood vaccinations

Q3

I took an active role in choosing my child's doctor.<br><br>READ OUT ANSWER OPTIONS	1. strongly agree<br>2. agree<br>3. neither agree nor disagree<br>4. disagree<br>5. Strongly disagree<br>6. doesn't want to disclose<br>7. doesn't know<br>8. missing	J'ai joué un rôle actif en choisissant le médecin de mon enfant.<br><br>LIRE TOUTES LES REPONSES POSSIBLES	1. Tout à fait d'accord<br>2. D'accord<br>3. Ni en accord, ni en désaccord<br>4. pas d'accord<br>5. Pas du tout d'accord<br>6. ne veut pas divulguer<br>7. ne sait pas<br>8. missing

For peer review only

Ich habe eine aktive Rolle dabei gespielt, den Arzt/die Ärztin für mein Kind auszusuchen.<br><br>ANTWORTMÖGLIC HKEITEN VORLESEN	1. stimme stark zu<br>2. stimme zu<br>3. weder noch<br>4. stimme nicht zu<br>5. stimme gar nicht zu<br>6. möchte die Frage nicht beantworten<br>7. weiss nicht<br>8. keine Antwort	Ho assunto un ruolo attivo quando si è trattato di scegliere il medico di mio/a figlio/a<br><br>LEGGERE LE RISPOSTE POSSIBILI	1. Fortemente d'accordo<br>2. d'accordo<br>3. né d'accordo né in disaccordo<br>4. in disaccordo<br>5. Fortemente in disaccordo<br>6. non vuole rivelare<br>7. non sa<br>8. mancante

Asked in:	✓ Childhood vaccinations

Q4

I chose a doctor for my child who shares my views on health.<br><br>READ OUT ANSWER OPTIONS	1. strongly agree<br>2. agree<br>3. neither agree nor disagree<br>4. disagree<br>5. Strongly disagree<br>6. doesn't want to disclose<br>7. doesn't know<br>8. missing	J'ai choisi un médecin qui partage mes perspectives sur la santé.<br><br>LIRE TOUTES LES REPONSES POSSIBLES	1. Tout à fait d'accord<br>2. D'accord<br>3. Ni en accord, ni en désaccord<br>4. pas d'accord<br>5. Pas du tout d'accord<br>6. ne veut pas divulguer<br>7. ne sait pas<br>8. missing
Ich habe einen Arzt/ eine Ärztin ausgesucht, die meine Ansichten zum Thema Gesundheit teilt.<br><br>ANTWORTMÖGLIC HKEITEN VORLESEN	1. stimme stark zu<br>2. stimme zu<br>3. weder noch<br>4. stimme nicht zu<br>5. stimme gar nicht zu<br>6. möchte die Frage nicht beantworten<br>7. weiss nicht<br>8. keine Antwort	Ho scelto un medico per mio/a figlio/a che condivide le mie stesse opinioni sulla salute<br><br>LEGGERE LE RISPOSTE POSSIBILI	1. Fortemente d'accordo<br>2. d'accordo<br>3. né d'accordo né in disaccordo<br>4. in disaccordo<br>5. Fortemente in disaccordo<br>6. non vuole rivelare<br>7. non sa<br>8. mancante

Asked in:	✓ Childhood vaccinations

Q5

For peer review only

If I disagree or am uncertain about the advice of a nurse or a doctor, I am comfortable saying so.<br><br>READ OUT ANSWER OPTIONS	1. strongly agree<br>2. agree<br>3. neither agree nor disagree<br>4. disagree<br>5. Strongly disagree<br>6. doesn't want to disclose<br>7. doesn't know<br>8. missing	Si je ne suis pas d'accord ou ne suis pas sûr.e concernant les conseils d'une infirmière ou d'un médecin, je suis suffisamment à l'aise pour le dire.<br><br>LIRE TOUTES LES REPONSES POSSIBLES	1. Tout à fait d'accord<br>2. D'accord<br>3. Ni en accord, ni en désaccord<br>4. pas d'accord<br>5. Pas du tout d'accord<br>6. ne veut pas divulguer<br>7. ne sait pas<br>8. missing
Wenn ich mit dem Rat eines Arztes oder einer Krankenschwester nicht einverstanden bin, oder diesbezüglich unsicher bin, traue ich mich das zu sagen.<br><br>ANTWORTMÖGLICHKEITEN VORLESEN	1. stimme stark zu<br>2. stimme zu<br>3. weder noch<br>4. stimme nicht zu<br>5. stimme gar nicht zu<br>6. möchte die Frage nicht beantworten<br>7. weiss nicht<br>8. keine Antwort	Se non sono d'accordo o se ho dei dubbi riguardo ai consigli di un infermiere/a o di un medico, non mi pongo problemi a dirlo.<br><br>LEGGERE LE RISPOSTE POSSIBILI	1. Fortemente d'accordo<br>2. d'accordo<br>3. né d'accordo né in disaccordo<br>4. in disaccordo<br>5. Fortemente in disaccordo<br>6. non vuole rivelare<br>7. non sa<br>8. mancante

Asked in:	✓ Childhood vaccinations

Q6

How many of the recommended well-child visits from birth until now has your child completed?<br><br>READ OUT ANSWER OPTIONS	1. All recommended visits<br>2. Some but maybe not all recommended visits<br>3. None of them<br>4. doesn't want to disclose<br>5. doesn't know<br>6. missing	Dès sa naissance et jusqu'à présent, à combien de contrôles de santé recommandés routines votre enfant est-il allé?	1. Tous les contrôles recommandés<br>2. Quelques-uns mais pas tous les contrôles recommandés<br>3. Aucun des contrôles

For peer review only

		LIRE TOUTES LES REPONSES POSSIBLES	recommandés<br>4. Ne veut pas divulguer<br>5. ne sait pas<br>6. missing
Wie viele der empfohlenen Vorsorgeuntersuchungen hat Ihr Kind von Geburt an bis jetzt erhalten?<br><br>ANTWORTMÖGLICHKEITEN VORLESEN	1. alle empfohlenen Vorsorgeuntersuchungen<br>2. Einige aber vielleicht nicht alle empfohlenen Vorsorgeuntersuchungen<br>3. Keine der empfohlenen Vorsorgeuntersuchungen<br>4. möchte die Frage nicht beantworten<br>5. weiss nicht<br>6. keine Antwort	Quante delle visite mediche periodiche raccomandate per suo/a figlio/a ha effettuato dalla nascita fino ad oggi?<br><br>LEGGERE LE RISPOSTE POSSIBILI	1. Tutte le visite raccomandate<br>2. Alcune ma forse non tutte le visite raccomandate<br>3. Nessuna di queste<br>4. non vuole rivelare<br>5. non sa<br>6. mancante

Asked in:	✓ Childhood vaccinations

Q7

Did you take your child to the doctor for a child development checkup between the ages of 11 and 14?	1. Yes<br>2. No<br>3. doesn't want to disclose<br>4. doesn't know<br>5. missing	Avez-vous amené votre enfant chez le médecin pour un contrôle de santé routine entre ses 11 et 14 ans?<br><br>IL S'AGIT ICI D'UN CONTROLE NORMAL ET NON PAS UNE VISITE D'URGENCE OU POUR UNE MALADIE	1. Oui<br>2. Non<br>3. ne veut pas divulguer<br>4. ne sait pas<br>5. missing

For peer review only

Haben Sie Ihr Kind im Alter zwischen 11 und 14 für eine Vorsorgeuntersuchung zum Arzt gebracht?	1. ja<br>2. nein<br>3. möchte die Frage nicht beantworten<br>4. weiss nicht<br>5. keine Antwort	Ha portato suo/a figlio/a ad effettuare una visita di controllo medico tra gli 11 e i 14 anni?	1. Si<br>2. No<br>3. non vuole rivelare<br>4. non sa<br>5. mancante

Asked in:	✓ HPV parent

Q8

When [name of target child] was an infant (0-2 years old), which of the following childcare options did you use? Please indicate all that apply.<br><br>READ OUT ANSWER OPTIONS	1. I (or my partner) stayed home with him/her.<br>2. Other family cared for him/her.<br>3. A nanny cared for him/her in my home.<br>4. He/she attended a small, home-based day care.<br>5. He/she attended private day care.<br>6. He/she attended public day care.<br>7. other<br>8. doesn't want to disclose<br>9. doesn't know<br>10. missing	Quand [name of target child] était un bébé (entre 0 et 2 ans), auxquels des services de garde d'enfance suivants avez-vous eu recours?<br><br>LIRE TOUTES LES REPONSES POSSIBLES	1. Soit moi, soit mon/ma partenaire l'a gardé à la maison.<br>2. Un autre membre de la famille l'a gardé.<br>3. Une nounou ou une nourrice l'a gardé chez nous.<br>4. Il/elle allait chez une maman de jour<br>5. Il/elle allait en garderie privée.<br>6. Il/elle allait en garderie publique<br>7. Autre<br>8. ne veut pas divulguer<br>9. ne sait pas<br>10. missing
Als [name of target child] ein Kleinkind war (0-2 Jahre alt), wurde es regelmässig von jemand anderem betreut ?	1. Ich blieb zuhause /mein(e) Partner(in) blieb zuhause<br>2. Jemand anderes aus der Familie schaute zu ihr/ihm	Quando [name of target child] era un neonato (da 0 a due anni d'età), quali delle seguenti opzioni di cura infantile ha utilizzato?	1. Io (o il mio partner) siamo stati a casa con lui/lei<br>2. Altra famiglia si è occupata di lui/lei<br>3. Una tata si è occupata di

ANTWORTMÖGLIC HKEITEN VORLESEN	3. Eine Nanny schaute zu ihm/ihr<br>4. Er/sie besuchte eine Tagesmutter<br>5. Er/sie war in der privaten Kinderkrippe/Spielgruppe<br>6. Er/sie war in der öffentlichen Kinderkrippe/Spielgruppe<br>7. anderes<br>8. möchte die Frage nicht beantworten<br>9. weiss nicht<br>10. keine Antwort	LEGGERE LE RISPOSTE POSSIBILI	lui/lei a casa mia<br>4. Ha frequentato una famiglia diurna<br>5. Lui/lei ha frequentato l'asilo nido privato<br>6. Lui/lei ha frequentato l'asilo nido pubblico<br>7. altro<br>8. non vuole rivelare<br>9. non sa<br>10. mancante

Asked in:	✓ Childhood vaccinations

If Q8 = 4, 5 or 6, ask Q9

Q9

And how old was he/she when he/she started day care?		Et quel âge avait-il/elle quand il/elle est entré.e en garderie? [i.e. entré.e en collectivité en dehors de la maison]	
Und wie alt war er/sie als er/sie in die Krippe/Spielgruppe kam?		Che età aveva quando ha incominciato l'asilo?	

Asked in:	✓ Childhood vaccinations

Theme 6: Provider questions

Q1

For the purposes of our study, we are interested in providers who practice conventional medicine, also referred to as allopathic or biomedical medicine, and providers who practice complementary and/or alternative medicine.		Pour cette étude, nous nous intéressons aux médecins et aux praticiens qui pratiquent la médecine conventionnelle, également appelée la médecine allopathique ou biomédicale, et aux médecins et praticiens qui pratiquent la médecine complémentaire et/ou alternative.	
Im Rahmen dieser Studie sind wir sowohl an Ärztinnen und Ärzten interessiert, die Schulmedizin praktizieren, wie auch an Praktikern und Ärztinnen und Ärzten, die Komplementär- oder Alternativmedizin ausüben.		Per gli obbiettivi del nostro studio, siamo interessati ai professionisti che praticano medicina convenzionale, anche riferita alla medicina allopatica e biomedicina, e professionisti che praticano medicina complementare e/o alternativa.	

Asked in:	✓ Provider

For peer review only

Q2

In order to obtain more background about you and your practices, could you please tell me if you are a licensed medical doctor in Switzerland?	1. Yes<br>2. No<br>3. missing	Pour que nous puissions avoir plus d'informations sur vous et vos pratiques, pourriez-vous m'indiquer si vous êtes un médecin autorisé à pratiquer la	1. Oui<br>2. Non<br>3. pas de réponse

		médecine en Suisse?	
Um etwas mehr über Sie und Ihre Praxis zu erfahren, möchten wir wissen, ob Sie ein in der Schweiz anerkannter Arzt/eine anerkannte Ärztin sind?	1. Ja<br>2. Nein<br>3. keine Antwort	Potrebbe per cortesia dirmi se è un medico con licenza Svizzera?	1. Sì<br>2. No<br>3. mancante

Asked in:	✓ Provider

Q3

Have you undertaken any additional specialist training in any discipline of complementary and/or alternative medicine?	1. Yes<br>2. No<br>3. missing	Avez-vous effectué une formation supplémentaire pour obtenir une spécialisation dans une discipline de la médecine complémentaire et/ou alternative ?	1. Oui<br>2. Non<br>3. pas de réponse
Haben Sie zusätzliche spezielle Weiterbildungen in einem komplementärmedizinischen oder alternativen Bereich gemacht?	1. Ja<br>2. Nein<br>3. keine Antwort	Ha intrapreso una formazione supplementare in una qualsiasi disciplina di medicina complementare e/o alternativa?	1. Sì<br>2. No<br>3. mancante

Asked in:	✓ Provider

If Q3 = 1, ask Q4

Q4

Which ones?	1. Anthroposophic medicine	Lesquelles?	1. la médecine anthroposophique

For peer review only - http://bmjopen.bmj.com/site/about/guidelines.xhtml

READ ITEM BY ITEM, CHECK IF YES FOR EACH.	2. Traditional Chinese Medicine / Acupuncture<br>3. Homeopathic medicine<br>4. Phytotherapy (i.e. plant-based/herbal remedies)<br>5. other(s)<br>6. no answer	LIRE CHAQUE REPONSE INDIVIDUELLE, COCHER SI OUI	2. l'acupuncture et pharmacothérapie chinoise<br>3. l'homéopathie<br>4. la phytothérapie<br>5. d'autres(s)<br>6. pas de réponse
Welche?<br>ANTWORTMÖGLICHKEITEN VORLESEN, ANKREUZEN FALLS JA	1. Anthroposophische Medizin<br>2. Traditionelle Chinesische Medizin / Akupunktur<br>3. Homöopathie<br>4. Phytotherapie (pflanzliche Arzneien)<br>5. Anderes:<br>6. Keine Antwort	Quali?<br>LEGGERE LE RISPOSTE POSSIBILI	1. Medicina antroposofica<br>2. Medicina tradizionale cinese / agopuntura<br>3. Medicina omeopatica<br>4. Fitoterapia (ovvero medicina a base di piante / erbe)<br>5. Altro/i<br>6. mancante

Asked in:	✓ Provider

If Q4 = 5, ask Q5

Q5

Please specify:		Précisez s.v.p	
Bitte präzisieren:		Per favore specifichi:	

Asked in:	✓ Provider

If Q2=2, ask Q6

Q6

Ok. Thank you. Do you provide any complementary	1. Yes<br>2. No	D'accord. Merci pour vos	1. Oui<br>2. Non

For peer review only

or alternative medicines to your patients?.	3. missing	réponses. Est-ce que vous employez de la médecine complémentaire et/ou alternative avec vos patients ?	3. pas de réponse
Vielen Dank. Bieten Sie ihren Patienten/Patientinnen irgendwelche komplementärmedizinischen oder alternativen Therapien an?	1. Ja<br>2. Nein<br>3. keine Antwort	Ok. Thank you. Do you provide any complementary or alternative medicines to your patients?	1. Sì<br>2. No<br>3. mancante

Asked in:	✓ Provider

If Q6 = 1, ask Q7

Q7

Which ones?<br><br>READ ITEM BY ITEM, CHECK IF YES FOR EACH.	1. Anthroposophic medicine<br>2. Traditional Chinese Medicine / Acupuncture<br>3. Homeopathic medicine<br>4. Phytotherapy (i.e. plant-based/herbal remedies)<br>5. other(s)<br>6. no answer	Lesquelles?<br><br>LIRE CHAQUE REPONSE INDIVIDUELLE, COCHER SI OUI	1. la médecine anthroposophique<br>2. l'acupuncture et pharmacothérapie chinoise<br>3. l'homéopathie<br>4. la phytothérapie<br>5. d'autres(s)<br>6. pas de réponse
Welche?<br><br>ANTWORTMÖGLICHKEITEN VORLESEN, ANKREUZEN FALLS JA	1. Anthroposophische Medizin<br>2. Traditionelle Chinesische Medizin / Akupunktur<br>3. Homöopathie	Quali?<br><br>LEGGERE LE RISPOSTE POSSIBILI	1. Medicina antroposofica<br>2. Medicina tradizionale cinese / agopuntura<br>3. Medicina omeopatica

For peer review only

	4. Phytotherapie (pflanzliche Arzneien)<br>5. Anderes:<br>6. Keine Antwort		4. Fitoterapia (ovvero medicina a base di piante / erbe)<br>5. Altro/i<br>6. mancante

Asked in:	✓ Provider

If Q7 = 5, ask Q8

Q8

Please specify:		Précisez s.v.p	
Bitte präzisieren:		Per favore specifichi:	

Asked in:	✓ Provider

If Q1 = 2, ask Q9

Q9

What type of complementary and/or alternative therapies do you provide to your patients?<br><br>READ ITEM BY ITEM, CHECK IF YES FOR EACH.	1. Anthroposophic medicine<br>2. Traditional Chinese Medicine / Acupuncture<br>3. Homeopathic medicine<br>4. Phytotherapy (i.e. plant-based/herbal remedies)<br>5. other(s)<br>6. no answer	Quel type de thérapies complémentaires et/ou alternatives fournissez-vous à vos patients ?<br><br>LIRE CHAQUE REPONSE INDIVIDUELLE, COCHER SI OUI	1. la médecine anthroposophique<br>2. l'acupuncture et pharmacothérapie chinoise<br>3. l'homéopathie<br>4. la phytothérapie<br>5. d'autres(s)<br>6. pas de réponse
Welche komplementären und/oder alternativen Therapien bieten Sie ihren Patienten und Patientinnen an?	1. Anthroposophische Medizin<br>2. Traditionelle Chinesische Medizin / Akupunktur<br>3. Homöopathie	Che tipo di terapie complementari e/o alternative	1. Medicina antroposofica<br>2. Medicina tradizionale cinese / agopuntura

For peer review only

ANTWORTMÖGLICHKEITEN VORLESEN, ANKREUZEN FALLS JA	4. Phytotherapie (pflanzliche Arzneien)<br>5. Anderes:<br>6. Keine Antwort	offre ai suoi pazienti?<br><br>LEGGERE LE RISPOSTE POSSIBILI	3. Medicina omeopatica<br>4. Fitoterapia (ovvero medicina a base di piante / erbe)<br>5. Altro/i<br>6. mancante

Asked in:	✓ Provider

If Q9 = 5, ask Q10

Q10

Please specify:		Précisez s.v.p	
Bitte präzisieren:		Per favore specifichi:	

Asked in:	✓ Provider

For peer review only

Formatting checklist

1. **Author information:** Have you got an ORCID iD (all BMJ journals publishing primarily original research now mandate ORCID iDs for all submitting authors at the time of article submission). **Yes. However, we were unable to link the authors to their ORCID iDs (listed below) through the online platform because we did not have this option.** Have you provided details of all of your co-authors? **Yes.** Is the information that you have entered into ScholarOne the same as the information on the manuscript title page? **Yes.**

Michael Deml
michael.deml@swisstph.ch
https://orcid.org/0000-0003-2224-8173

Kristen Jafflin
kristen.jafflin@swisstph.ch
https://orcid.org/0000-0003-2105-4262

Sonja Merten
sonja.merten@swisstph.ch
https://orcid.org/0000-0003-4115-106X

Benedikt Huber
Benedikt.Huber@h-fr.ch
https://orcid.org/0000-0003-2952-3879

Andrea Buhl
andrea.buhl@unibas.ch
https://orcid.org/0000-0002-7907-7902

Eleonora Frau
eleonora.frau@stud.unibas.ch
https://orcid.org/0000-0003-4040-5937

Valérie Mettraux
valerie.mettraux@stud.unibas.ch
https://orcid.org/0000-0003-4065-428X

Joanna Sonderegger
joanna.sonderegger@stud.unibas.ch
https://orcid.org/0000-0001-8003-3176

Paulina Kliem
paulina.kliem@stud.unibas.ch

For peer review only

https://orcid.org/0000-0003-3521-5880

Rachele Cattalani
rachele.cattalani@stud.unibas.ch
https://orcid.org/0000-0002-8581-7890

Daniel Krüerke
daniel.krueerke@klinik-arlesheim.ch
https://orcid.org/0000-0002-3600-0403

Constanze Pfeiffer
constanze.pfeiffer@unibas.ch
https://orcid.org/0000-0002-9487-2867

Claudine Burton-Jeangros
claudine.jeangros@unige.ch
https://orcid.org/0000-0003-2258-9728

Philip E. Tarr
philip.tarr@unibas.ch
https://orcid.org/0000-0002-1488-5407

2. **Manuscript length and formatting**: Have you provided your abstract in the correct format? **Yes/** Have you supplied any required additional information for your article type, such as key messages? **N/A** Have you checked that your manuscript doesn't exceed the requirements for word count, number of tables and/or figures, and number of references? **Yes. The manuscript exceeds the word count, and we have noted this in the cover letter to the editor.**

3. **Tables**: Are your tables in an editable format? Have you embedded them into the main word document? Have they been cited in the text? Have you provided appropriate table legends? Have you uploaded any lengthy tables as supplementary files for online publication? **N/A**

4. **Figures**: Have you uploaded figures separately from the text? **Yes. As indicated in the online platform, we have included the figure at the end of the PDF document of the manuscript.** Have they been supplied in an acceptable format and are they of sufficient quality? **Yes.** Are they suitable for black and white reproduction (unless you intend to pay any required fees for colour printing)? **No.**

For peer review only - http://bmjopen.bmj.com/site/about/guidelines.xhtml

The figure is colour dependent. We could pay additional required fees for colour printing, if necessary. Have the files been labelled appropriately? **Yes.** Have the figures been cited in the text? **Yes.** Have you provided appropriate figure legends? **YES. Lines 960-961 of the submitted PDF.**

5. **References**: Have all of the references been cited in the text? **Yes.**

6. **Supplementary files**: Have you supplied these in an acceptable format? **Yes.** Have they been cited in the main text? **N/A. The supplementary files we have included are budgetary documents, ethics approvals, and a manuscript reporting initial qualitative results that we have submitted to Social Science & Medicine for the editor to review.**

7. **Statements**: Have you included the necessary statements relating to author contributorship, competing interests and funding, data sharing, patient consent and ethical approval? **YES.**

8. **Acknowledgements:** Have you acknowledged all contributors who do not meet the criteria for authorship? **YES.** Have you acknowledged if your work has been previously presented at a conference/published as a conference abstract? **YES. This is indicated in the submitted manuscript (lines 1010 – 1022).**

9. **Suggested reviewers:** Have you suggested reviewers for your paper (if required by the journal)? **We suggest the reviewers below:**

Eve Dubé, Eve.Dube@inspq.qc.ca
Pierre Verger, pierre.verger@inserm.fr
Suzanne Suggs, suzanne.suggs@usi.ch
Alessandro Diana, Alessandro.Diana@hcuge.ch

For peer review only

10. **Research reporting checklists**: Have you either provided the appropriate statement for your study type, or explained why a checklist isn't required? **N/A**

11. **Reproducing figures:** Have you obtained permission from the copyright holder to re-use any previously published material? Has the source been acknowledged? **N/A**

For peer review only
